# Patterns of expansion and expression divergence in the plant polygalacturonase gene family

**DOI:** 10.1186/gb-2006-7-9-r87

**Published:** 2006-09-29

**Authors:** Joonyup Kim, Shin-Han Shiu, Sharon Thoma, Wen-Hsiung Li, Sara E Patterson

**Affiliations:** 1 Department of Horticulture, Cellular and Molecular Biology Program, University of Wisconsin-Madison, Madison, WI 53706, USA; 2 Department of Plant Biology, Michigan State University, East Lansing, MI 48824, USA; 3 Department of Zoology, University of Wisconsin-Madison, Madison, WI 53706, USA; 4 Department of Ecology and Evolution, University of Chicago, Chicago, IL 60637, USA

## Abstract

**Background:**

Polygalacturonases (PGs) belong to a large gene family in plants and are believed to be responsible for various cell separation processes. PG activities have been shown to be associated with a wide range of plant developmental programs such as seed germination, organ abscission, pod and anther dehiscence, pollen grain maturation, fruit softening and decay, xylem cell formation, and pollen tube growth, thus illustrating divergent roles for members of this gene family. A close look at phylogenetic relationships among *Arabidopsis *and rice PGs accompanied by analysis of expression data provides an opportunity to address key questions on the evolution and functions of duplicate genes.

**Results:**

We found that both tandem and whole-genome duplications contribute significantly to the expansion of this gene family but are associated with substantial gene losses. In addition, there are at least 21 PGs in the common ancestor of *Arabidopsis *and rice. We have also determined the relationships between *Arabidopsis *and rice PGs and their expression patterns in *Arabidopsis *to provide insights into the functional divergence between members of this gene family. By evaluating expression in five *Arabidopsis *tissues and during five stages of abscission, we found overlapping but distinct expression patterns for most of the different PGs.

**Conclusion:**

Expression data suggest specialized roles or subfunctionalization for each PG gene member. PGs derived from whole genome duplication tend to have more similar expression patterns than those derived from tandem duplications. Our findings suggest that PG duplicates underwent rapid expression divergence and that the mechanisms of duplication affect the divergence rate.

## Abstract 2

Analysis of Arabidopsis and rice polygalacturonases suggests that polygalacturonases duplicates underwent rapid expression divergence and that the mechanisms of duplication affect the divergence rate.

## Background

The functions and regulation of cell wall hydrolytic enzymes have intrigued plant scientists for decades. These enzymes cleave the bonds between the polymers that make up the cell wall, and include polygalacturonases (PGs), beta-1, 4-endoglucanases, pectate lyases, pectin methylesterases, and xyloglucan endo-transglycosylases [1]. As a consequence of their action, cell wall extensibility and cell-cell adhesion can be altered leading to cell wall loosening that results in cell elongation, sloughing of cells at the root tip, fruit softening, and fruit decay [2-4]. Cell separation processes also contribute to important agricultural traits such as pollen dehiscence and abscission of organs including leaves, floral parts, and fruits [5-7]. In addition, these enzymes are hypothesized to be involved in general housekeeping functions in plants [8].

Among these hydrolytic enzymes, the PGs belong to one of the largest hydrolase families [9,10]. PG activities have been shown to be associated with a wide range of plant developmental programs such as seed germination, organ abscission, pod and anther dehiscence, pollen grain maturation, xylem cell formation, and pollen tube growth [5,11-13]. Over-expression of a PG in apple (*Malus domestica*) has resulted in alterations in leaf morphology and premature leaf shedding [14]. Interestingly, the functions of PGs are not restricted to the control of cell growth and development as they are also reported to be associated with wound responses [15] and host-parasite interactions [16]. These findings illustrate the divergent and important roles of PGs in plants.

PGs have been identified in various plants including *Arabidopsis*, pea and tomato [5,17]. In both tomato and *Arabidopsis *it has been determined that many PGs are located within tandem clusters [9,18]. In addition to tandem duplication, the *Arabidopsis *genome contains large blocks of related regions derived from whole genome duplication events [17,19,20]. In this study, we conducted a comparative analysis of PGs from *Arabidopsis *and rice to address several key questions on the evolution and function of this gene family. We compared the PGs from *Arabidopsis *and rice to determine the pattern of expansion and the extent of PG losses prior and subsequent to the divergence between these two species. To uncover the mechanisms that contributed to the expansion of this gene family, we examined the distribution of PGs on *Arabidopsis *chromosomes in conjunction with the large-scale duplicated blocks. Torki *et al*. [9] have suggested that a group of related PGs tend to be expressed in the flowers and flower buds, while PGs expressed in vegetative tissues belong to other groups. The implication is that the diverse functions of PGs may be a consequence of differential expression. This expression divergence and/or subfunctionalization most likely contribute to the retention of PG duplicates [21,22]. To evaluate the degree of spatial expression divergence between PGs, we conducted RT-PCR analysis on all 66 *Arabidopsis *PG genes in five non-overlapping tissue types. To supplement the RT-PCR expression data, we also examined expression tags generated from other large-scale sequencing projects. Finally, we analyzed expression at five stages of floral organ abscission to assess the degree of temporal expression divergence among members of this gene family.

## Results and discussion

### Expansion of the PG family in *Arabidopsis *and rice

**Figure 1:**
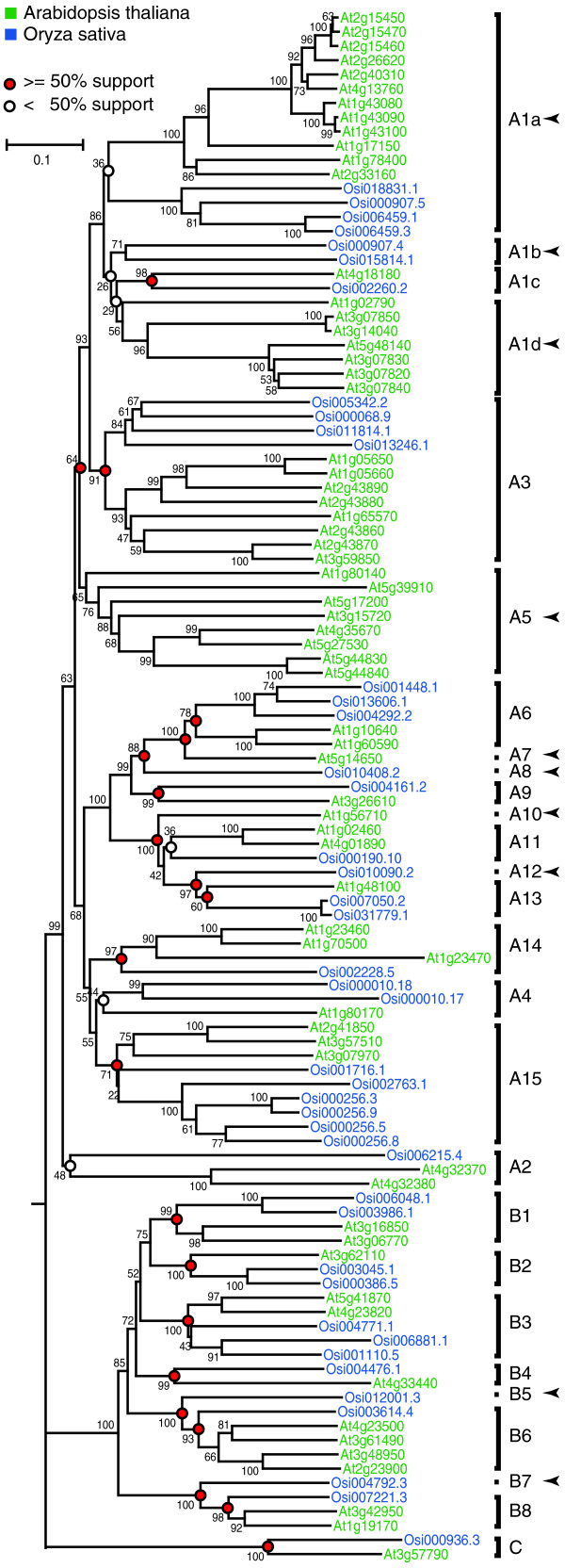
The phylogeny of *Arabidopsis *and rice PGs. The amino acid sequences for the glycosyl hydrolase 28 family motif were aligned. The phylogeny was generated using neighbor-joining algorithm with 1,000 bootstrap replicates. Sequences are color-coded according to the key. The plant PGs are classified into three major groups and multiple clades. The clades were defined by identifying nodes representing speciation events (circles, see Results section for criteria). For these nodes, red circles indicate that the bootstrap support for the subtending branches is higher than 50% and indicate the criteria for least number of common ancestral PGs between rice and *Arabidopsis*. The nodes are labeled with white circles if the bootstrap support is less than 50%. Arrowheads indicate clades that contain only sequences for one of the two plants.

To investigate the relationships among PGs and the extent of lineage-specific expansion in rice and *Arabidopsis*, we identified PGs from the GenBank polypeptide records and the genomes of *Arabidopsis *and rice (*Oryza sativa *subsp. *indica*). All PGs identified contain GH28 domains that are approximately 340 amino acids long and encompass approximately 75% of the average PG coding sequence (for lists of genes used in this analysis, see Figure 1 and Additional data files 1,2 and 8). According to the phylogenetic relationships of bacterial, fungal, metazoan, and plant PGs (Additional data file 3), we found that the 66 *Arabidopsis *and 59 rice PGs fall into three distinct groups (Figure 1, groups A, B, and C). Sixteen of the rice PGs contain more than one glycosyl hydrolase 28 (GH28) domain and were regarded as mis-annotated tandem repeats. It should be noted that the rice PGs were derived from the shotgun sequencing of the *O. indica *genome that was estimated to be 95% complete [23]. We identified the nodes that lead to *Arabidopsis*-specific and rice-specific clades and predict that these represent the divergence point between these two species. We have designated the clades defined by such nodes as AO (*Arabidopsis*-*Oryza*) orthologous groups. For example, in the A3 clade there exists one *Arabidopsis *subclade and one rice subclade, and we predict that only one ancestral A3 sequence was present before the divergence between *Arabidopsis *and rice. However, gene losses could have occurred and therefore some PGs may be present in the *Arabidopsis*-rice common ancestor but later lost in either *Arabidopsis *or rice (Figure 1, arrowheads). Therefore, *Arabidopsis *(A, indicating loss(es) in rice) and rice (O, indicating loss(es) in *Arabidopsis*) clades were also identified based on their sister group relationships to the AO clades. Since the clades that we defined are most likely orthologous groups (Figure 1, red circles), the number of clades reflects that there were at least 21 ancestral PGs before the *Arabidopsis*-rice split. Further expansion of this gene family occurred after the split as suggested by the duplication events in the lineage-specific branches that reside within each clade. It should be noted that some clades such as the A1 clade were not defined based on the AO clade-based criteria because the nodes within had relatively low bootstrap supports (<50%). If we assumed these less well-supported nodes are correct, there are 27 ancestral PGs.

### Duplication mechanisms accounting for the PG family expansion

**Figure 2:**
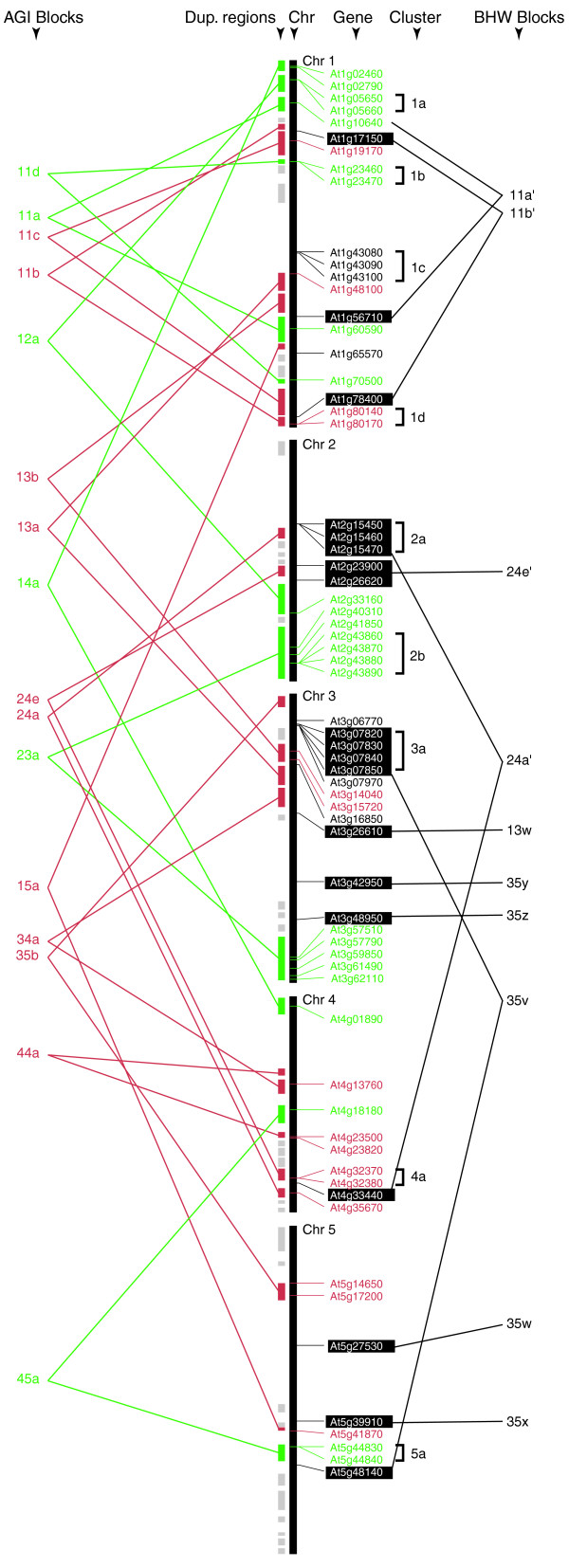
Mechanisms of *Arabidopsis *PG family expansion. The locations of *Arabidopsis *PGs are indicated on the *Arabidopsis *chromosomes. The tandem clusters are also indicated. They are color-coded based on the following scheme: PGs found in both duplicated regions of a block pair (green); PGs found in only one duplicated region of a block pair (red); and no PG is located in these blocks (gray). PGs covered by AGI blocks are either red or green, while PGs covered by BHW but not AGI blocks are with white text and black-boxed background. If PGs are found in both duplicated regions of a block, the gene names are linked. In addition, these gene names are italicized if they belong to the same clade. PGs that are not found in either AGI or BHW blocks are shown in black text. Tandem duplications are indicated by cluster designation. BHW block names were modified from the original designations of Blanc *et al*. [20]. BHW block names with a prime indicate that they overlap with AGI blocks of the same names. The reference for the block names can be found in Additional data file 2.

Examination of the distribution of the *Arabidopsis *PGs on all five chromosomes indicates a non-random distribution of many PGs (Figure 2). More than one third of the *Arabidopsis *PGs (24 of 66) have at least one related sequence within ten predicted genes, and these 24 genes fall into nine clusters that range from two to four genes per cluster (Figure 2, column cluster). In most cases, these physically associated PGs are from the same clades; however, there are five exceptions including genes in clusters 1d, 2b and 3a (Figure 2). In these cases, some members within the cluster are not closest relatives. Besides these 24 tandem repeated sequences, all remaining PGs are at least 100 genes apart. This bimodal distribution of PG physical distances and relationships between closely linked genes suggests that the 24 closely linked PGs are derived from tandem duplications.

In addition to tandem duplications, it has been shown that the *Arabidopsis *genome is the product of several rounds of polyploidization or whole-genome duplications [17,19,20]. To determine the contribution of these large-scale duplications, we mapped *Arabidopsis *PGs to the duplicated blocks established in two independent studies. The first dataset from the Arabidopsis Genome Initiative [17] contains 31 blocks (AGI blocks), and forty *Arabidopsis *PGs fall in 16 of the AGI blocks (Figure 2, indicated in red and green). Blocks from the second dataset from Blanc *et al*. [20] are designated as BHW (after Blanc, Hokamp, Wolfe) blocks, and 19 PGs were found in 10 BHW blocks (Figure 2, shaded). The AGI and BHW blocks were identified using different approaches and their combined use increases the coverage of duplicated regions. As a result, nearly 90% (59 out of 66) of *Arabidopsis *PGs are covered in the 26 AGI and BHW blocks.

**Figure 3:**
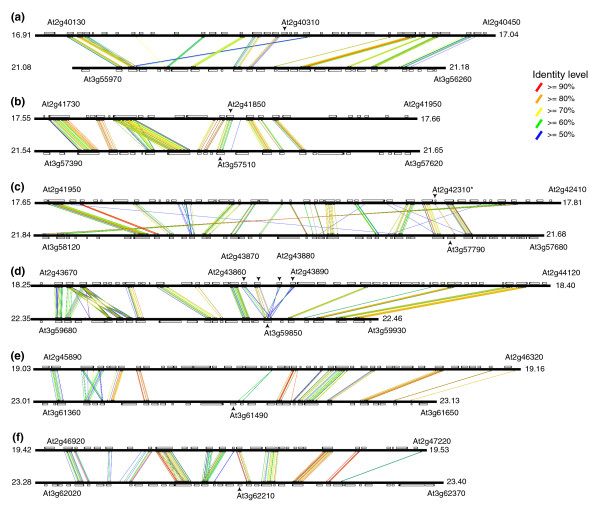
Collinearity of PGs in AGI block 23a. After locating areas with similarities in the block 23a (see also Additional data file 4), six distinct PG-containing regions were defined. **(a) **At2g40310 does not have PG in the collinear region. **(b) **At2g41850 and At3g57510 are located in collinear regions. **(c) **The 3' end of At3g57790 is highly similar to At2g42310*, a truncated PG that is likely a pseudogene. **(d) **A tandem of four PGs (At2g43860, At2g43870, At2g43880, At2g43890) is located in the collinear region with At3g59850. **(e) **At3g61490 does not have any PG in the corresponding collinear region. **(f) **At3g62210 does not have any PG in the collinear region. For each region pair, the solid black bars are the chromosomes (top: chromosome 2, bottom: chromosome 3) flanked by the starting and ending positions in Mb. The annotated genes are drawn to scale in a rectangular box on the chromosome and in each box the thicker black line indicates the 3' position of the gene. The names are only shown for PGs and the starting and ending genes in each block pair. The areas that are at least 30 amino acids long with at least 50% identity are linked by colored lines based on their identity levels (see key).

Within these 26 duplicated blocks, 29 PGs are found in both duplicated regions of ten block pairs. To investigate the origin of PGs in these ten block pairs, we conducted similarity searches between regions of each pair to determine if PGs mapped to the corresponding duplicated regions, and if their neighboring genes were arranged collinearly (Figure 3; see also (Additional data file 4) for all comparisons). Sixteen PGs in five of these block pairs are clearly located in such collinear regions, indicating that they were derived from large-scale duplication of their associated blocks. For example, AGI block 23a contains nine PGs in six corresponding duplicated regions that show extensive collinearity (Figure 3). In Figure 3b, At2g41850 and At3g57510 are flanked by paralogous genes that are arranged collinearly, indicating that they were products of a block duplication. This is also true for a tandem cluster of four PGs and a PG singleton shown in Figure 3d. Interestingly, At3g57790 corresponds to At2g43210, a potential pseudogene lacking the signal peptide and the bulk of the PG catalytic domain (Figure 3c). We also observed that there are 23 duplicated block pairs with asymmetrical distribution (Additional data file 4). Among them, 16 block pairs have PGs on only one of the blocks (Figure 2 and (Additional data file 4)): ten for AGI and six for BHW blocks. For the remaining seven block pairs, the PGs are found on both blocks but are not arranged in a collinear fashion. Taken together, these findings clearly indicate that many members of the PG family are derived from large-scale duplication events. However, quite a few of them were not retained.

### PG expression in *Arabidopsis *tissues

**Figure 4:**
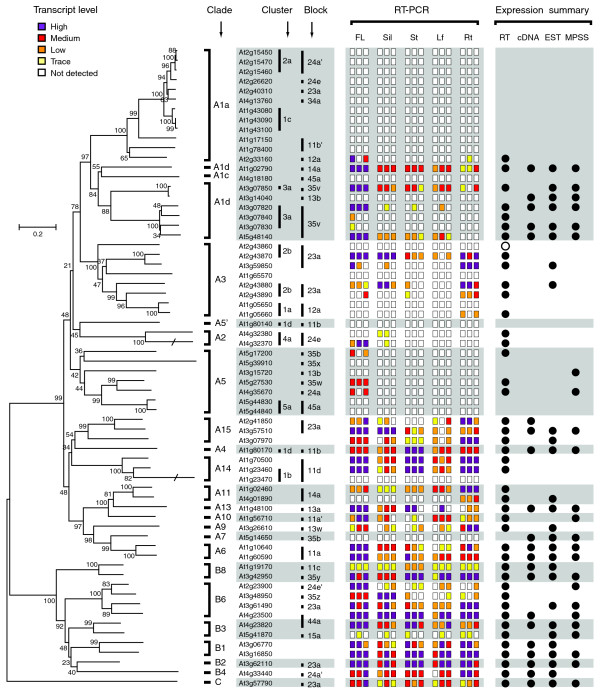
The phylogeny and expression patterns of *Arabidopsis *PGs. The phylogeny was generated using all *Arabidopsis *PGs with *Erwinia peh1 *as the outgroup. The clade classification, cluster and block designation are also shown. The levels of transcripts are classified into five categories as shown in the key. The tissue source abbreviations are as follows: Fl, flower; Si, silique; St, stem; Lf, rosette and cauline leaf; Rt, root; gDNA, genomic DNA. For each gene, three colored rectangles represent the level of RT-PCR products from three independent biological replications for each tissue type. On the right, the solid black circles indicate the presence of the four different expression tags. RT-PCR data are from this study and a solid circle represents repeatable expression from one or more of the six tissue types analyzed including expression in At2g43860 from suspension cultures. Open circles represent expression that was only detected in one of the RT-PCR reactions yet verified by sequencing. cDNAs, ESTs and MPSS tags were obtained from SIGnAL, GenBank, and the *Arabidopsis *MPSS project websites, respectively. Branches that were shortened are intersected with a solidus (/).

**Figure 5:**
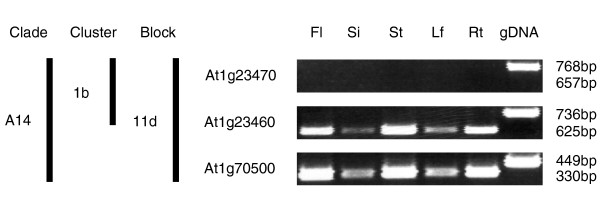
RT-PCR of PGs in five tissue types. The competitive RT-PCR, using both cDNA and gDNA templates, is demonstrated. The expression pattern of PGs in the clade A14 is variable except At1g23470, which has no detectable expression in all five tissue types. RT-PCR product sizes are indicated to the right of the figure. Tissue source abbreviations are as in Figure 4.

The size of the plant PG family and the patterns of PG duplication in *Arabidopsis *indicate that the PG family expanded in both *Arabidopsis *and rice after their divergence. The continuous expansion of this gene family raises an intriguing question on the mechanisms of duplicate retention and their functions in plants. Since retention may be due to functional divergence between duplicate copies, it is possible that PG functional divergence can be, in part, attributed to expression divergence. To evaluate the degree of expression divergence between PG duplicates, we analyzed the expression of all 66 *Arabidopsis *PGs in five tissue types (flowers, siliques, inflorescence stems, rosette and cauline leaves, and roots) with RT-PCR (Figure 4 and Additional data file 5). PCR reactions were repeated at least three times for each gene in each tissue type, and all primers were tested using genomic DNA as a positive control (see Figure 5). In addition, PCR products of 40 of the 43 PGs were sequenced to verify their identity. We found that 23 PGs did not have detectable RT-PCR products in any of the five tissue types tested. We further tested the expression of these 23 PGs in a T87 suspension culture cell line that had been previously shown to have >60% genes expressed [24]. Only one PG (At2g43860) was detected. To rule out the possibility of faulty primer designs, a second primer set was designed for each of these 23 PGs, but none led to detectable products.

To complement the RT-PCR approach, we also examined the expression tags that were publicly available including full-length cDNAs, expressed sequence tags (ESTs), and massive parallel signature sequencing (MPSS) tags (Additional data file 6). The presence of RT-PCR products or other expression tags is shown in Figure 4 (far right-hand panel). Among these four different expression measures, the RT-PCR approach detects the highest number of PGs. In the 43 PGs with RT-PCR products, other expression tags support only 30 of them. In addition, only three PGs have cDNA, ESTs, and/or MPSS but not RT-PCR products. These findings indicate that RT-PCR is the most sensitive approach with a relatively low false-negative rate. For further analyses, we consider a PG expressed if two out of three of the RT-PCR reactions had detectable products (42) or if its expression is supported by the presence of either cDNA or EST (three). Based on these criteria, 45 PGs had detectable expression (Figure 4). Approximately 50% of these expressed PGs are found in all five tissues and 20% have relatively higher level of expression in more than one tissue. In addition, more than 50% of expressed PGs have high level of expression in floral tissues, 40% in root tissue, 16% in stem and 12% in silique. Only nine PGs (approximately 20%) are found in only one tissue type (Figure 4). These findings indicate that most PGs have rather wide expression patterns and the expression level seems to be generally higher in floral tissues. The complexity of expression patterns represented in Figure 4 emphasizes the need for additional interpretation, and is the basis for the statistical analyses described below for the expression data.

### Effects of duplication mechanisms on gene expression

**Figure 6:**
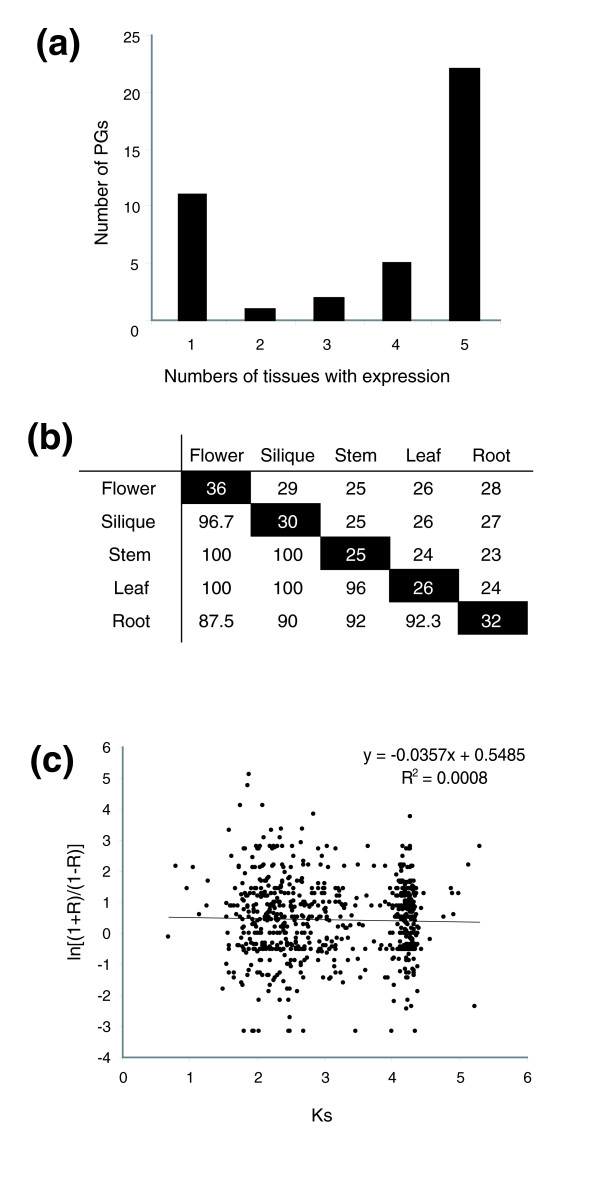
Expression of PGs shared among tissues and the correlation between expression patterns and the *Ks*. **(a) **Overlapping expression of PGs - the majority of expressed PGs are found in all five tissues tested. **(b) **Pairwise comparisons of tissues with PGs - the numbers in black boxes represent the number of PGs expressed in indicated tissues. The numbers in the upper-right half are the number of PGs expressed in both tissues specified in the top row and in the leftmost column. The numbers in the lower-left half are the percent overlap between two tissues. **(c) **The relationships between the *Ks *and transformed correlations in expression patterns - the *Ks *values were determined for all PG pairs. The correlations between expression patterns were calculated for all PG pairs and transformed as described in the Materials and methods. The formulae for the best fit and the correlation coefficient determined by linear regression are shown on the top right corner.

While it was anticipated that more closely related genes would tend to have similar expression patterns, we did not find significant correlation between the synonymous substitution rate (*Ks*) and the expression profile (Figure 6). In addition, to evaluate the relationships between *Ks *and expression correlation using all PG pairs, we also reached the same conclusion after partitioning the data as within clade (*r *= -0.119, *p *= 0.39), between clade (*r *= 0.002, *p *= 0.58), or reciprocal best matches (*r *= -0.4389, *p *= 0.12). This finding indicates that expression patterns have diverged quickly after PG duplications. In particular, significantly fewer PGs in tandem clusters were expressed when compared with those not in clusters (Table 1; Fisher's exact test; *p *= 0.0326). In several cases, the tandem duplicated regions have one relatively highly expressed gene while the rest have either low expression levels or no RT-PCR products. For example, in the 1b tandem cluster of clade A14, At1g23460 is highly expressed while At1g23470 does not have any detectable expression. Curiously, we found that related PGs found in duplicated blocks tend to have similar expression patterns at the tissue level. For example, in block 11d clade A14, At1g23460 and At1g70500 have nearly identical expression profiles (Figure 4). We selected 18 PG pairs that were derived from tandem or large-scale block duplication to compare their expression divergence. Among nine pairs in large-scale duplicated blocks, the expression pattern is significantly different in only one pair (Table 2). Among the nine pairs derived from tandem duplications, the *t*-test could only be conducted for four pairs because several of the tandem duplicates had no detectable expression. In addition to two pairs with significant differences (*p *< 0.05), three pairs with only one of the tandem duplicates expressed are also classified as pairs showing expression divergence. Therefore, excluding two pairs with no expression for both duplicates, five out of seven tandem pairs have divergent expression. Significantly fewer PG pairs derived from tandem duplications have similar expression patterns compared with those derived from large-scale duplications (Fisher's exact test; *p *< 0.01). Therefore, tandemly duplicated PGs have higher levels of expression divergence compared with PGs derived from large-scale duplications. These findings suggest that duplication mechanisms contribute to divergence of expression patterns differently.

### Developmentally regulated expression divergence among PGs expressed in abscission zone

**Figure 7:**
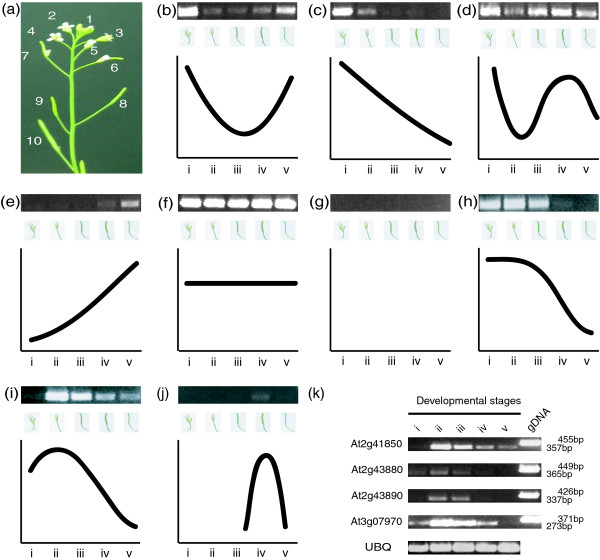
RT-PCR on floral organ abscission zones representing five unique stages of development. Expression of 43 PGs is examined in the abscission zones at five different stages of floral organ abscission as determined by position on the inflorescence, where position one represents anthesis and larger numbers are progressively older flowers **(a)**. Five developmental stages were examined with the RT-PCR; *i *(position1/2) and *ii *(position 4/5), pre-abscission, *iii *(position 7/8), during abscission, *iv *(position 0/11), and *v *(position 13/14) post-abscission. Expression during the abscission process is classified into nine different unique patterns shown in (b) to (j); the gene names are provided in Additional data file 7. PGs specifically up-regulated during the abscission process are shown with RT-PCR products (k).

So far, our expression analyses were performed in five widely different tissues. To further expand our understanding of PG expression, we took a close look at 43 of the expressed PGs in the abscission zones of flowers and developing siliques at five developmental stages during floral organ abscission (Figure 7a). During the abscission process there are discrete stages when cell wall loosening and cell wall dissolution occurs, thus providing an excellent biological system to look at more subtle changes in the regulation of cell separation. And indeed, this analysis allowed us to discern differences in expression between PGs that had been initially regarded as similar due to limitations in resolution (Figure 7). For example, at the tissue level, At1g23460 and At1g70500, from block 11d clade A14 were regarded as having nearly identical expression profiles. However, when we examined five stages of abscission, these genes have distinct profiles (Figure 7c and 7e, Additional data file 7).

We determined that there are nine unique patterns of expression for the PGs during the five stages of abscission that are shown in Figure 7 and Additional data file 7. Eight PGs display high levels of expression at anthesis, low levels during the events of cell separation, and high levels post abscission as depicted in Figure 7b. These genes are all from independent clades except two sets: At1g19170 and At3g42950 (B8), and At2g23900 and At3g48950 (B6). In Figure 7c, 7 PGs show initial high expression at anthesis that decreases steadily during abscission, while in Figure 7d, PG expression (At1g02460, At1g56710, and At3g61490) initially decreases right before abscission and then increases after the loss of floral organs or during what is described as post abscission repair. In Figure 7e, two PGs (At1g23460 and At1g10640) have very low or undetectable expression during anthesis that goes up continually during abscission. Other patterns include ten PGs with constitutive expression (Figure 7f), and six PGs with no expression (Figure 7g). Last, we observed three patterns of expression that correlated with unique changes during the process of abscission (Figure 7h,i,j). In Figure 7h, high levels of gene expression correlate with cell wall loosening or the earliest steps of abscission, while in Figure 7i highest levels of gene expression correlate with cell separation or loss of floral organs. In Figure 7j, it is only at around positions 10 and 11 that we observe detectable gene expression, and this correlates with predicted stages of cell repair [25].

Taken together, expression divergence between PGs that show no difference at the tissue level were revealed when we examined PG expression at different developmental stages of abscission, thus indicating duplication mechanisms contribute to divergence of expression differently. Our findings also provide candidate PGs important for different abscission stages. More importantly, the expression divergence between duplicate genes in general appears to be under-estimated in expression studies due to the limitations in resolution.

## Conclusion

### PG family expansion history

PGs fall into several taxon-specific clades where eubacterial, fungal, and plant PGs organize into different clusters [10]. We have hypothesized that there were approximately 21 PGs present in the immediate common ancestor of *Arabidopsis *and rice, and when additional monocots and dicots are sequenced, we will be able to have a more accurate estimate of the ancestral family size. Since *Arabidopsis *and rice diverged more than 150 million years ago (MYA), gene conversion events that occurred soon after divergence of these two lineages will be much rarer than those that occurred in a lineage-specific fashion.

By examining the physical locations of *Arabidopsis *PGs and their relationships to the proposed large-scale duplication patterns, we found that tandem duplications and large-scale duplications were two of the major factors responsible for the expansion of the PG family in *Arabidopsis*. This is similar to other gene families such as the NBS-LRR [26] and the RLK/Pelle gene family [27]. Among duplicates in the same tandem cluster, nearly all belong to the same PG clades or are close relatives of each other. The only exception is At1g80140 and At1g80170 in cluster 1d, suggesting that they are tandem duplicates that formed before the *Arabidopsis*-rice split. Most of the PGs (59) are located within 26 duplicated block pairs (Table 1). However, the comparison of gene contents between duplicated blocks in each pair indicates that 22 PGs are distributed asymmetrically in ten of these duplicated block pairs, thus suggesting gene losses. The rest of the duplicated block pairs contain PGs in both duplicated regions. Since only 13 of these PGs are collinear, our findings suggest that large-scale duplications did contribute to some expansion of the PG family but gene losses occurred frequently. Members of each PG pair (either one-to-one or one-to-many) located in collinear regions are from the same clade. Since a clade is defined as the PG ancestral unit right before the divergence between *Arabidopsis *and rice, the blocks harboring these PGs would be duplicated after the split between these two plants. Blanc *et al*. [20] assigned duplicated gene pairs to blocks and used synonymous substitution rates to establish the block age. We found that 17 PGs were in 'recent' blocks that duplicated after the split between the *Arabidopsis *and rice lineages (Additional data file 4). This correlation is consistent with our interpretation based on a phylogenetic approach.

In the cases where PGs were present in only one of the collinear regions, it is likely that the absence of PGs was due to gene losses, and almost 80% of the PGs generated by large-scale duplications could have been lost in *Arabidopsis*. These findings are consistent with the high duplicate loss rate in the *Arabidopsis *genome [28,29]. In addition, the collinear regions flanking PGs are generally larger than the corresponding regions without PGs (considering the numbers of genes or physical distances between the two genes flanking the PGs that were collinear), thus suggesting that the deletion of chromosome regions contributes to PG loss. Another explanation for the asymmetrical distribution of PGs in blocks is that they were inserted *de novo *through an alternative mechanism such as retro-transposition; however, this is unlikely, as all of the plant PGs have multiple introns.

### Divergence of expression pattern after duplications

Although a large number of PG duplicates were lost, there is a net gain in the PG family size after the split between *Arabidopsis *and rice, and thus, the immediate question is how were these duplicates retained? The fate of duplicated genes varies and depends on the selection constraints [21,22]. Since one third of the *Arabidopsis *PGs do not have any evidence of expression, these genes could be pseudogenes. However, some of them have diverged substantially from their closest relatives with large synonymous substitution rates and have most likely persisted beyond the time frame of pseudogenization in *Arabidopsis *proposed to be a million years [30]. Meanwhile, PGs without evidence of expression may be present in tissues not sampled or induced under untested conditions. A closer look at other developmental events involving cell wall degradation, cell separation or cell wall loosening may provide additional insights.

There is mounting evidence that retention of duplicated genes may be due to acquisition of novel functions, partitioning of original functions, or both. The contribution of differential expression in retaining duplicated genes has been hypothesized more than 25 years ago [31,32]. More recently, Force *et al*. [33] proposed the DDC (Duplication/Degeneration/Complementation) model predicting that genes sharing overlapping but distinct expression patterns will be retained due to the partitioning of ancestral expression profiles. In our study, we found that two thirds of the *Arabidopsis *PGs are expressed and almost three quarters of these expressed PGs are detected in at least three tissues. If the AtGenExpress microarray data for *Arabidopsis *is considered [34], five additional PGs are likely expressed using a stringent intensity cutoff (data not shown). Among the PGs that are expressed rather ubiquitously, related PGs in general have overlapping but distinct expression profiles, consistent with the prediction of the DDC model, although it is possible that some expression differences are due to gain of expression rather than loss. In any case, divergent expression among closely related PGs is evident in the different developmental stages of abscission. It has also been reported more recently that duplicated genes tend to have more similar expression patterns when the *Ks *is relatively small [35,36]. However, in the PG family, the more recent duplicates do not necessarily have more similar expression patterns. The expression correlation breaks down even more when we examine the expression profiles of PGs in different developmental stages of the abscission process. This lack of correlation may be attributed to relatively long divergence time (large *Ks *value) between PG duplicates and the lack of statistical power, because a much smaller number of genes are examined compared with an analysis of the whole genome. In addition, we suggest that the mechanism of gene duplication appears to contribute differently to expression divergence. The number of expressed PGs is significantly lower if they are located in tandem repeats. On the other hand, PGs with similar tissue expression patterns tend to be localized to corresponding large-scale duplicated blocks. One possible mechanism for this difference in expression pattern conservation may be the fact that tandem duplication may or may not allow the duplication of whole promoter regions and coding sequences. On the other hand, large-scale duplication involves the duplication of multiple genes together with their promoter and/or enhancer elements. Thus, tandem duplications will result in faster expression divergence than large-scale duplications, and that large-scale duplications ultimately lead to "fine tuning" of gene expression. Another potential explanation for the differences in expression may be due to differences in gene silencing. Homology-dependent gene silencing is a common phenomenon in plants [37]. Since the average sequence divergence between tandem repeats is smaller than that of large-scale duplications (data not shown), one might also argue that tandemly duplicated genes tend to be silenced at a higher frequency.

Functional studies have established that plant PGs are involved in diverse roles including plant growth and development, wounding responses, and plant-microbe interactions [4]. Although the PG family members have substantial overlap in tissue-level expression even between distantly related members, when we analyzed distinct developmental stages of abscission we were able to discern unique patterns of expression. These findings suggest that although even if there may be functional overlap between PGs, substantial expression divergence contributed to their retention and probably their functions. Given the number of PGs and the complexity of plant tissues and cell types, it is likely that PGs expressed in the same tissues have subtle differences in their temporal or spatial profiles. This is consistent with the PG expression patterns in different developmental stages of abscission. Alternatively, these seemingly co-expressed PGs may have also diverged at the biochemical levels, such as their catalytic properties. In this study, we used genome sequence information combined with gene expression to provide a framework to unravel the complexity of gene family function. By careful analysis we have been able to take a family of 66 genes and identify four members (Figure 7i) that have unique changes just as cell wall loosening and cell wall dissolution is predicted to occur; thus presenting a small subset of genes for further studies on abscission. Additional analyses in the temporal and spatial patterns of expression in other tissues, their biochemical properties, and in the biological functions of these genes will lead to novel insights regarding functional divergence and conservation in this gene family.

## Materials and methods

### Sequence selection, alignment, and phylogenetic analysis

Representative PGs were the sequences in the seed alignment of glycosyl hydrolase family 28 (GH28) from Pfam database [38]. The representative set was used as query sequences to conduct BLAST searches [39] against polypeptide sequences of *A. thaliana *for candidate PGs from Munich Information Center for Protein Sequences (MIPS) [40]. All sequences with E values less than one were regarded as candidate PGs and further analyzed with the Pfam HMM models from GenBank polypeptide sequences; The PGs of *O. sativa *subsp. *indica *were identified from predicted coding sequences obtained from Dr. W. Karlowski in MIPS *Oryza sativa *Database (MosDB) [41] with a similar procedure outlined above. The rice PG sequences appeared highly redundant, and thus almost 30% of the entries that were more than 99% identical at the nucleotide level were eliminated from further analysis. For a list of PGs, including redundant entries, see Additional data files 1 and 8. The protein sequences of PGs identified were aligned against the Pfam GH28 seed alignments using the profile alignment function of ClustalW [42]. The GH28 domain sequence alignments of rice and *Arabidopsis *PGs analyzed can be found in Additional data file 8. The phylogeny of all PGs identified was generated with MEGA2 [43] using the neighbor-joining algorithm [44] with 1,000 bootstrap replicates. Poisson correction for multiple substitutions was used. Sequence gaps were treated as missing characters. Both the *Arabidopsis*-rice and *Arabidopsis*-only trees were rooted with *Erwinia peh1*.

### Mapping chromosome location and duplicated blocks

Two large-scale duplication datasets were used. The first is based on the analysis of the Arabidopsis Genome Initiative [17] that was provided by Heiko Schoof and MIPS/Institute of Bioinformatics, Germany. The correspondence between block names given in this study and those in the original analysis, and the starting and ending gene names for these blocks are given in Additional data file 2. The second is based on Blanc *et al*. [20] and is available from [45]. The collinearity of blocks that contain PGs in corresponding duplicated regions was determined using tBLASTn. For these blocks, the nucleotide sequences of one of the duplicated regions were used as query to search against a translated database built from the nucleotide sequence of the other region. To increase the number of High Scoring Pairs recovered, the query sequences were split into 5 kb windows. The matching areas (at least 50 amino acids long and 60% identical) of blocks that contain PGs in the corresponding duplicated regions are shown in Additional data file 4. After identifying the collinear regions surrounding PGs, we took at least 100 kb regions surrounding PGs and their corresponding duplication regions, regardless of the presence of PGs, and repeated the BLAST analysis splitting query sequences into 1 kb windows. Matching areas were defined as similar regions at least 30 amino acids long.

### Plant materials and growth

*Arabidopsis *ecotype Columbia (COL) was used for this study and plants grown as described by Patterson and Bleecker [25]. T87 suspension-cultured cell lines were derived from COL ecotype [46,47] and provided by Sebastian Bednarek (University of Wisconsin, Madison, WI, USA). The abscission zones of developing flowers and siliques were collected by removing the primary inflorescence from the plant, and then trimming each individual sample within 0.75 mm +/- 0.25 of the floral abscission zone on both sides. Trimmed samples were immediately frozen in liquid nitrogen and stored at -80°C until further analysis.

### Nucleic acid isolation and quantification

Plant tissue was frozen in liquid nitrogen, ground and added to TES-Lysis (50 mM Tris pH 8, 5 mM EDTA, 50 mM NaCl, 1% (w/v) SDS, 1% w/v sarkosyl) followed by extraction with a phenol:chloroform:isoamyl alcohol mix (25:24:1). Samples were centrifuged for 5 minutes at (12,000 *g*) and the resulting aqueous phase was extracted twice with chloroform:isoamyl alcohol (24:1). Nucleic acids were precipitated at 4°C with isopropanol and 10 M NH_4_OAc (one-third volume) and resuspended in TE. One-half volume of 6.0 M LiCl was added to the sample, incubated at 4°C for 4 hours, and then centrifuged 15 minutes at 12,000 *g*. DNA (supernatant fraction) was precipitated by adding 10 M NH_4_OAc (1/3 volume) and ethanol, and RNA (pellet) was washed with ethanol and resuspended in DEPC-treated H20 (1 μg/μl). DNA and RNA yields were quantified using a Smart Spec 3000 Biorad (Hercules, CA, USA). Nucleic acid quality was assessed by gel electrophoresis.

### RT-PCR analysis

A quantity of 1 μg of each RNA sample was used to prepare cDNA by modifying standard procedures [48]. First strand synthesis was carried out using 500 μg/ml of an 18 mer oligo dT primer (IDT, Coralville, IA, USA). Resulting cDNAs were diluted 1:2 and 1 μl was added as template for a standard 20 μl PCR reaction. For each gene, primers were designed that flanked an intron in the genomic DNA similar to that described by Wang *et al*. [48]. Since the mRNA and genomic copy of a gene share identical primer sites, they had comparable amplification efficiencies in the PCR reaction and were distinguishable by size. Reactions were incubated at 95°C for 5 minutes, and cycled 28 or 36 times as follows: 94°C for 3 minutes, annealing temperature for 30 seconds, 72°C for 2 minutes. After the last cycle, reactions were incubated at 72°C for 7 minutes. Annealing temperatures and cycle numbers were optimized and are shown in Additional data file 5. A quantity of 10 μl of each PCR reaction was analyzed by gel electrophoresis, and the relative levels of PCR products were recorded.

### DNA sequencing

PCR products were excised from the gel, cleaned using a Qiagen Gel extraction kit (Qiagen, Valencia, CA, USA) and sequenced directly as described below. Cycle sequencing reactions with a thermostable DNA polymerase and fluorescently labeled dideoxy terminators (BIG DYE Applied Biosystems, Foster, CA, USA) were carried out on each purified product or subcloned fragment. At2g43870 was subcloned into the PCR 4-TOPO vector (Invitrogen, Carlsbad, CA, USA) before sequencing. All reactions were outsourced to the UW-Madison Biotechnology Center and run on an ABI automated DNA sequencer.

### Expression tags of PGs and analysis

The cDNA sequences released by the SIGnAL database [49] were retrieved from GenBank. The predicted protein sequences of PGs were used to search against the cDNA sequences. The cDNAs for PGs are listed in part I of Additional data file6. The *Arabidopsis *ESTs were retrieved from GenBank (part II of Additional data file 6), and a BLAST search was conducted using the predicted coding sequences of PGs. All matches with more than 80% identity were inspected. After eliminating gaps longer than three from the alignments, cognate ESTs were defined as those that were top matches to the gene in question with at least 97% identity. The accessions, source tissue information for the matching ESTs, can be found in part II of Additional data file 6. The MPSS tags matching the PG genes were retrieved using a batch query script from the *Arabidopsis *MPSS database [50]. Only tags matching exons in the crick strand with levels significantly different from 0 were regarded as evidence of expression.

The PG expression levels as determined by RT-PCR were converted into 5 categories: high (4), medium (3), low (2), trace (1), and none (0). For each gene, the median (M) of the converted expression levels was used for all subsequent analyses. For each gene pair, the synonymous and non-synonymous substitution rates were determined using the yn00 phylogenetic analysis by maximum likelihood program PAML [51]. The Pearson correlation coefficient (*r*) was determined for each gene pair and transformed into ln [(1+R)/(1-R)] for linear repression analyses [35,36]. For determining the differences in expression patterns between tandemly duplicated and block-duplicated genes, we conducted *t*-tests for 18 PG pairs. For each tissue, the expression levels were considered if both or either one of the genes in a pair were expressed.

## Additional data files

The following additional data are available with the online version of this paper. Additional data file 1 lists PGs identified from Genbank protein records. Additional data file 2 is the BHW and AGI assignment of PGs to duplicated blocks in *Arabidopsis*. Additional data file 3 shows a phylogeny generated with all the PGs from fungi, bacteria, metazoa, and plants. Additional data file 4 shows a figure with the matching areas for duplicated blocks containing PGs in both regions. Additional data file 5 lists the primers used for the RT-PCR analysis. Additional data file 6 is summary of expression tags including a list of the PG cDNAs from *Arabidopsis *and a list of the PG cognate ESTs from *Arabidopsis*. Additional data file 7 lists PGs that are expressed in the floral organ abscission zones of *Arabidopsis *with their patterns of expression. Additional data file 8 shows GH28 domain sequence alignments of rice and *Arabidopsis *PGs analyzed.

## Supplementary Material

Additional data file 1 Table containing PGs (except *Arabidopsis *proteins) sorted according to their taxa. Click here for file

Additional data file 2 Table of assignment of PGs to duplicated blocks in *Arabidopsis*. Click here for file

Additional data file 3 Figure showing a phylogeny generated with all the PGs from fungi, bacteria, metazoa, and plants. Click here for file

Additional data file 4 Figure with the matching areas for duplicated blocks containing PGs in both regions. Click here for file

Additional data file 5 Primer pairs and conditions used to amplify PG genes. Click here for file

Additional data file 6 Table showing summary of expression tags and list of cDNAs for *Arabidopsis *PGs. Click here for file

Additional data file 7 A list of PGs that are expressed in the floral organ abscission zones of *Arabidopsis *with their patterns of expression. Click here for file

Additional data file 8 GH28 domain sequence alignments of rice and *Arabidopsis *PGs analyzed. Click here for file

## Figures and Tables

**Table 1:** Distribution and expression of *Arabidopsis *PG genes in duplicated regions

	Out of duplicated regions*		Within duplicated regions*			
			
			With match^†^		Without match^†^	
				
	Number of genes	Expression^‡^	Number of genes	Expression^‡^	Number of genes	Expression^‡^
Singular	4	3	11	9	27	21
Tandem	3	0	10	8	11	4
Total	7	3	21	17	38	25

*Duplicated regions are the regions that are covered by the AGI and BHW blocks. ^†^The presence (with match) or absence (without match) of PGs in collinear regions of each duplicated block pair as shown in Figure 4 and Additional data file 4. ^‡^Expression detected in at least two out of three RT-PCR reactions or supported by the presence of cDNA or EST tags.

**Table 2:** Expression (RT-PCR) of *Arabidopsis *PG genes in different clades

Set*	Gene1	Gene2	*Ks*^†^	t^‡^	*p *< 0.05^‡^
B1	At1g02460	At4g01890	1.0564	3.09	n
B2	At1g10640	At1g60590	1.252	-0.32	n
B3	At1g23460	At1g70500	0.8011	-0.73	n
B3	At1g23470	At1g70500	1.877	-14.70	y
B4	At2g41850	At3g57510	0.6805	-1.43	n
B5	At2g43860	At3g59850	2.1371	-3.00	n
B5	At2g43870	At3g59850	0.9534	2.13	n
B5	At2g43880	At3g59850	1.8279	1.00	n
B5	At2g43890	At3g59850	1.8308	-1.41	n
T1	At1g05650	At1g05660	0.2385	ND^§^	y
T2	At1g23460	At1g23470	0.878	6.53	y
T3	At2g43860	At2g43870	1.4013	-6.53	y
T4	At2g43880	At2g43890	4.2072	2.83	n
T5	At3g07820	At3g07830	0.5342	ND^§^	y
T5	At3g07820	At3g07840	0.4923	ND^§^	y
T5	At3g07830	At3g07840	0.457	ND	ND
T6	At4g32370	At4g32380	2.6336	0.73	n
T7	At5g44830	At5g44840	0.1626	ND	ND

*Each set contains genes that were duplicated through either local-scale block duplication (B) or tandem duplication (T). In duplicated blocks where a PG is collinear with a cluster, the one-to-many relationships are shown. For tandem clusters, all pairwise combinations are shown. ^†^*Ks*, synonymous substitution rate. ^‡^Differences in expression patterns significant (y) or not (n) for *t*-test with *df *= 2, *p *< 0.05 [52]. ND, not determined since both genes do not have detectable RT-PCR product or ^§^expression was documented for only one gene in the pair.
