# Supplementary material for: Patterns of expansion and expression divergence in the plant polygalacturonase gene family
Source: Genome Biol. 2006 Sep 29;7(9):R87. doi: 10.1186/gb-2006-7-9-r87 (PMC1794546; doi:10.1186/gb-2006-7-9-r87)
Supplement: Additional data file 1 — Table containing PGs (except Arabidopsis proteins) sorted according to their taxa. [file gb-2006-7-9-r87-S1.pdf]

SUPPLEMENT A. List of polygalacturonases identified from GenBank protein records except *Arabidopsis* proteins

These entries are sorted according to their taxa: BA, eubacteria; F, fungi; ME, metazoans; PL, plants. Number of PGases refers to the number of glycosyl hydrolase family 28 domains predicted by Pfam and SMART. Note column: *, redundant entries (also colored yellow); D, deprecated entries that GenBank deleted in current release; P, partial sequence that is not included in the alignments.

Accession	GI	Taxa	Note	Species name	Gene name	PGase #	Annotation
P27644	129937	BA		*Agrobacterium_tumefaciens*	N.A.	1	POLYGALACTURONASE
AAG60962	12620686	BA		*Bradyrhizobium_japonicum*	id636	1	ID636
AAB46984	1839180	BA		*Burkholderia_cepacia*	pehA	2	polygalacturonase
P18192	129933	BA		*Erwinia_carotovora*	Peh1	1	ENDO-POLYGALACTURONASE PRECURSOR
P26509	129936	BA		*Erwinia_carotovora*	PehA	1	ENDO-POLYGALACTURONASE PRECURSOR
3891426	3891426	BA		*Erwinia_carotovora_subsp._carotovora*	N.A.	1	N.A.
P15922	129749	BA		*Erwinia_chrysanthemi*	N.A.	1	EXO-POLY-ALPHA-D-GALACTURONOSIDASE PRECURSOR
AAF70169	7804881	BA		*Leptosphaeria_maculans*	pg1	1	endopolygalacturonase PG1
BAA74431	4185608	BA		*Pectobacterium_carotovorum*	peh	1	Peh
AAA03624	148472	BA	*	*Pectobacterium_carotovorum*	peh1	1	poly galacturonase
AAA57139	476012	BA	*	*Pectobacterium_carotovorum*	peh-1	1	endopolygalacturonase
JC1219	95574	BA		*Pectobacterium_carotovorum*	N.A.	1	polygalacturonase precursor
CAB99318	9437305	BA		*Pectobacterium_chrysanthemi*	pehV	1	polygalacturonase
CAB99319	9437306	BA		*Pectobacterium_chrysanthemi*	pehW	1	polygalacturonase
CAB99320	9437307	BA		*Pectobacterium_chrysanthemi*	pehX	1	exo-poly-a-D-galacturonosidase
P20041	129942	BA		*Ralstonia_solanacearum*	N.A.	1	POLYGALACTURONASE PRECURSOR
AAB51699	1621469	BA		*Rhizobium_vitis*	pehA	1	endo-polygalacturonase
S72635	7435342	BA		*Thermoanaerobacterium_thermosulfurigenes*	N.A.	1	exo-poly-alpha-galacturonosidase precursor
H72376	7462768	BA		*Thermotoga_maritima*	N.A.	1	hypothetical protein TM0437
AAC15064	3089553	BA		*Yersinia_enterocolitica*	N.A.	1	exopolygalacturonase
BAB32924	13160919	F		*Alternaria_alternata*	aapg-1	1	endopolygalacturonase
BAB32923	13160913	F		*Alternaria_citri*	acpg-1	1	endopolygalacturonase
AAC23565	3220207	F		*Aspergillus_aculeatus*	pga1	1	polygalacturonase
A55415	1078617	F		*Aspergillus_aculeatus*	rhgA	1	rhamnogalacturonase A precursor
Q00001	2499718	F	*	*Aspergillus_aculeatus*	rhgA	1	RHAMNOGALACTURONASE A PRECURSOR
2981975	2981975	F	*	*Aspergillus_aculeatus*	N.A.	1	N.A.
P41749	1172461	F		*Aspergillus_flavus*	N.A.	1	POLYGALACTURONASE A PRECURSOR
P41750	1172462	F		*Aspergillus_flavus*	N.A.	1	POLYGALACTURONASE B PRECURSOR
P26213	129932	F		*Aspergillus_niger*	PG1	1	POLYGALACTURONASE I PRECURSOR
P26214	129934	F		*Aspergillus_niger*	PG2	1	POLYGALACTURONASE II PRECURSOR
Q12554	3024382	F		*Aspergillus_niger*	PG3	1	POLYGALACTURONASE III PRECURSOR
CAB72125	6911543	F		*Aspergillus_niger*	pgaA	1	endo-polygalacturonase A
CAB72126	6911545	F		*Aspergillus_niger*	pgaB	1	endo-polygalacturonase B
CAB72931	6967121	F		*Aspergillus_niger*	pgaD	1	endo-polygalacturonase D
CAA74744	2909341	F		*Aspergillus_niger*	pgaE	1	endopolygalacturonase
CAA63911	2117031	F		*Aspergillus_niger*	rhgA	2	rhamnogalacturonase
CAA63912	2117033	F		*Aspergillus_niger*	rhgB	1	rhamnogalacturonase
BAA95407	7707787	F		*Aspergillus_niger_awamorii*	pgx2	1	polygalacturonase-X2
BAA95408	7707789	F		*Aspergillus_niger_awamorii*	ppas	1	protopectinase-AS
BAA34782	3970835	F		*Aspergillus_oryzae*	pgaB	1	polygalacturonase B
P35335	548489	F		*Aspergillus_oryzae*	N.A.	1	POLYGALACTURONASE PRECURSOR
P49575	1346703	F		*Aspergillus_parasiticus*	N.A.	1	POLYGALACTURONASE PRECURSOR

P19805	129935	F	*Aspergillus_tubingensis*	N.A.	1	POLYGALACTURONASE II PRECURSOR
Q00293	2499716	F	*Aspergillus_tubingensis*	N.A.	1	EXOPOLYGALACTURONASE PRECURSOR
S74208	7514890	F	*Aspergillus_tubingensis*	N.A.	1	galacturan 1,4-alpha-galacturonidase precursor
CAB65657	6686870	F	*Aspergillus_tubingensis*	xghA	1	endo-xylogalacturonan hydrolase
AAC64374	3282213	F	*Botryotinia_fuckeliana*	BcPGA1	1	endopolygalacturonase 1
AAC24950	3282215	F	*Botryotinia_fuckeliana*	BcPGA2	1	endopolygalacturonase 2
AAC24952	3282218	F	*Botryotinia_fuckeliana*	BcPGA3	1	endopolygalacturonase 3
AAC24953	3282222	F	*Botryotinia_fuckeliana*	BcPGA4	1	endopolygalacturonase 4
AAC24955	3282226	F	*Botryotinia_fuckeliana*	BcPGA5	1	endopolygalacturonase 5
AAC24956	3282228	F	*Botryotinia_fuckeliana*	BcPGA6	1	endopolygalacturonase 6
AAF05088	6175595	F	*Botryotinia_fuckeliana*	PGX	1	exo-polygalacturonase
AAB61358	2199504	F	*Botryotinia_fuckeliana*	N.A.	1	rhamnogalacturonan hydrolase
AAF68401	7716491	F	*Chondrostereum_purpureum*	epgA	1	endopolygalacturonase
AAF68402	7716493	F	*Chondrostereum_purpureum*	epgB	1	endopolygalacturonase
AAK29433	13540363	F	*Chondrostereum_purpureum*	epgB2	1	endopolygalacturonase
AAF68404	7716497	F	*Chondrostereum_purpureum*	epgD	1	endopolygalacturonase
JC5462	7514829	F	*Chondrostereum_purpureum*	N.A.	1	endopolygalacturonase I precursor
CAA71246	1752653	F	*Claviceps_purpurea*	pg1	1	polygalacturonase
CAA71247	1752654	F	*Claviceps_purpurea*	pg2	1	polygalacturonase
P26215	129938	F	*Cochliobolus_carbonum*	N.A.	1	POLYGALACTURONASE PRECURSOR
Q00359	2499717	F	*Cochliobolus_carbonum*	N.A.	1	EXOPOLYGALACTURONASE PRECURSOR
CAC14022	10933620	F	*Colletotrichum_gloeosporioides_f._sp._malvae*	PG2	1	endopolygalacturonase
Q00446	2499715	F	*Colletotrichum_lindemuthianum*	pg1	1	ENDOPOLYGALACTURONASE 1 PRECURSOR
CAA64727	2230787	F	*Colletotrichum_lindemuthianum*	pg2	1	endopolygalacturonase
AAB36616	1208810	F	*Cryphonectria_parasitica*	enpg-1	1	endopolygalacturonase
JC2498	1076242	F	*Cryptomeria_japonica*	cry_j_2	1	second major allergen Cry j II precursor
P43212	1171004	F	*Cryptomeria_japonica*	N.A.	1	POSSIBLE POLYGALACTURONASE PRECURSOR
BAA20555	2217913	F	*Fusarium_oxysporum*	N.A.	1	endopolygalacturonase
AAC05015	2078555	F	*Fusarium_oxysporum_f._sp._lycopersici*	PG1	1	endopolygalacturonase
AAC32604	3450879	F	*Fusarium_oxysporum_f._sp._lycopersici*	pgx1	1	exopolygalacturonase
AAC27792	3348099	F	*Fusarium_oxysporum_f._sp._lycopersici*	N.A.	1	endopolygalacturonase
AAF12737	13378332	F	*Fusarium_oxysporum_f._sp._radicis-lycopersici*	pg1	1	exopolygalacturonase
BAA20427	2196457	F	*Geotrichum_klebahnii*	PSE3	1	protopectinases SE3
AAG34307	11323212	F	*Gibberella_circinata*	fcpg	1	endopolygalacturonase
Q07181	585668	F	*Gibberella_fujikuroi*	N.A.	1	POLYGALACTURONASE PRECURSOR
CAA03900	2597957	F	*Kluyveromyces_marxianus*	epg1	1	endopolygalacturonase
AAC05833	2967835	F	*Ophiostoma_novo-ulmi*	pg	1	polygalacturonase
Q9Y718	7388011	F	*Penicillium_digitatum*	N.A.	1	POLYGALACTURONASE PRECURSOR
O59925	7388004	F	*Penicillium_expansum*	N.A.	1	POLYGALACTURONASE PRECURSOR
AAF06810	6289088	F	*Penicillium_griseoroseum*	pggI	1	polygalacturonase
AAF03895	6110608	F	*Penicillium_griseoroseum*	pggII	1	polygalacturonase
O93883	7388007	F	*Penicillium_griseoroseum*	N.A.	1	POLYGALACTURONASE PRECURSOR
O42824	7388002	F	*Penicillium_janthinellum*	N.A.	1	POLYGALACTURONASE PRECURSOR
Q9Y833	7388012	F	*Penicillium_olsonii*	N.A.	1	POLYGALACTURONASE 2 PRECURSOR
Q9Y834	7388013	F	*Penicillium_olsonii*	N.A.	1	POLYGALACTURONASE 1 PRECURSOR
NP_012687	6322613	F	*Saccharomyces_cerevisiae*	PGU1	1	Endo-polygalacturonase
S62742	2133329	F	*Sclerotinia_sclerotiorum*	PG2	1	endopolygalacturonase 2 precursor
S62743	2133330	F	*Sclerotinia_sclerotiorum*	PG3	1	endopolygalacturonase 3 precursor
CAA74019	2196886	F	*Sclerotinia_sclerotiorum*	pg5	1	endopolygalacturonase
Q12708	3024383	F	*Sclerotinia_sclerotiorum*	N.A.	1	ENDO-POLYGALACTURONASE PRECURSOR

AAG35693	11493894	ME		*Sitophilus_oryzae*	N.A.	1	endopolygalacturonase
AAM28240	21628922	ME		*Meloidogyne incognita*	MiPG1	1	polygalacturonase
CAA76930	4210806	ME		Phaedon cochleariae	N.A.	1	polygalacturonase
AAF71154	7959971	PL		*Actinidia_chinensis*	PGA	1	polygalacturonase A
AAF71155	7959973	PL		*Actinidia_chinensis*	PGA	1	polygalacturonase A
AAF71160	7959983	PL		*Actinidia_chinensis*	PGA	1	polygalacturonase A
AAF71156	7959975	PL		*Actinidia_chinensis*	PGB	1	polygalacturonase B
AAF71158	7959979	PL		*Actinidia_chinensis*	PGC	1	polygalacturonase C
AAF71159	7959981	PL	*	*Actinidia_chinensis*	PGC	1	polygalacturonase C
P35336	548488	PL		*Actinidia_chinensis*	N.A.	1	POLYGALACTURONASE PRECURSOR
CAA90272	1419408	PL		*Brassica_napus*	PGA/SAC66	1	Polygalacturonase
CAC05657	9967518	PL		*Brassica_napus*	PGAZ	1	endopolygalacturonase
P35337	548490	PL		*Brassica_napus*	N.A.	1	POLYGALACTURONASE PRECURSOR
CAA65072	1212786	PL		*Brassica_napus*	RDPG1	1	polygalacturonase
X98373	1403141	PL	*	*Brassica_napus*	RDPG1	1	endo-polygalacturonidase
JC7100	7520823	PL		*Chamaecyparis_obtusa*	Cha_o_2	1	polygalacturonase Cha o 2
T08203	7435389	PL		*Cucumis_melo*	MPG1?	1	polygalacturonase precursor
T08213	7435390	PL		*Cucumis_melo*	MPG2?	1	polygalacturonase precursor
T08215	7435383	PL		*Cucumis_melo*	MPG3	1	polygalacturonase 3 precursor
BAA88472	6624205	PL		*Cucumis_sativus*	CUPG1	1	poly galacturonase
AAD46483	5669846	PL		*Glycine_max*	PG1	1	polygalacturonase PG1
AAD46484	5669848	PL		*Glycine_max*	PG2	1	polygalacturonase PG2
Q39766	3024385	PL		*Gossypium_barbadense*	N.A.	1	POLYGALACTURONASE PRECURSOR
Q39786	3024386	PL		*Gossypium_hirsutum*	N.A.	1	POLYGALACTURONASE PRECURSOR
CAC05582	9955725	PL		*Juniperus_ashei*	jna2	1	pollen major allergen 2 protein
P05117	129939	PL		*Lycopersicon_esculentum*	PG2A	1	POLYGALACTURONASE 2A PRECURSOR
AAC70951	3834451	PL		*Lycopersicon_esculentum*	PG7	1	polygalacturonase 7
AAC28903	2459811	PL		*Lycopersicon_esculentum*	TAPG1	1	polygalacturonase 1
AAB09575	1575705	PL		*Lycopersicon_esculentum*	TAPG2	1	abscission polygalacturonase
T04319	7435391	PL		*Lycopersicon_esculentum*	TAPG3	1	polygalacturonase TAPG3
T04320	7435392	PL	*	*Lycopersicon_esculentum*	TAPG4	1	polygalacturonase TAPG4
T07591	7435388	PL		*Lycopersicon_esculentum*	TAPG4	1	polygalacturonase TAPG4 precursor
T04322	7435393	PL		*Lycopersicon_esculentum*	TAPG5	1	polygalacturonase TAPG5
225933	225933	PL	*	*Lycopersicon_esculentum*	PG2A	1	N.A.
AAD17250	4325090	PL		*Lycopersicon_esculentum*	N.A.	1	polygalacturonase
S57806	1362096	PL	*	*Lycopersicon_esculentum*	TAPG1	1	polygalacturonase precursor
T05906	7435394	PL		*Lycopersicon_esculentum*	N.A.	1	probable polygalacturonase
AAF61444	7381227	PL		*Lycopersicon_esculentum*	XOPG1	1	polygalacturonase
P48978	1346704	PL		*Malus_domestica*	N.A.	1	POLYGALACTURONASE PRECURSOR
T09654	7435398	PL		*Medicago_sativa*	PG3	1	polygalacturonase PG3 precursor
Q40312	3024387	PL		*Medicago_sativa*	N.A.	1	POLYGALACTURONASE PRECURSOR
AAG14416	10185719	PL		*Nicotiana_tabacum*	NTS1	1	NTS1 protein
S32006	322765	PL	D	*Nicotiana_tabacum*	N.A.	1	polygalacturonase
S32008	322767	PL	*	*Nicotiana_tabacum*	N.A.	1	polygalacturonase
S32010	322769	PL	*	*Nicotiana_tabacum*	N.A.	1	polygalacturonase
Q05967	548491	PL	*	*Nicotiana_tabacum*	N.A.	1	POLYGALACTURONASE PRECURSOR
S46533	1084423	PL	*	*Nicotiana_tabacum*	N.A.	1	polygalacturonase precursor (clone G27.1,| G27.2)
P24548	129941	PL		*Oenothera_organensis*	N.A.	1	EXOPOLYGALACTURONASE
AAA32914	166951	PL		*Persea_americana*	N.A.	1	polygalacturonase
Q02096	400758	PL	*	*Persea_americana*	N.A.	1	POLYGALACTURONASE PRECURSOR
S31195	419745	PL	*	*Persea_americana*	N.A.	1	polygalacturonase

AAD02559	4105806	PL	P	*Petunia_x_hybrida*	PGPS/NH19	1	PGPS/NH19
CAB42886	4826572	PL		*Phleum_pratense*	pg	1	polygalacturonase
AAK50769	13958032	PL		*Pisum_sativum*	Ppg1	2	polygalacturonase
P48979	1346705	PL		*Prunus_persica*	N.A.	1	POLYGALACTURONASE PRECURSOR
S71524	2147957	PL	D	*Prunus_persica*	N.A.	1	polygalacturonase
AAC64184	3747093	PL		*Prunus_persica*	N.A.	1	endopolygalacturonase
S71523	2147956	PL		*Prunus_persica*	N.A.	1	polygalacturonase [similarity]
CAA11846	3164119	PL		*Rubus_idaeus*	RAS3	1	polygalacturonase
BAA89476	6714524	PL		*Salix_gilgiana*	SgPG1	1	polygalacturonase
BAA89477	6714526	PL		*Salix_gilgiana*	SgPG2	1	polygalacturonase
BAA89478	6714528	PL		*Salix_gilgiana*	SgPG3	1	polygalacturonase
BAA89479	6714530	PL		*Salix_gilgiana*	SgPG4	1	polygalacturonase
CAA40910	22422	PL	*	*Zea_mays*	PG1	1	polygalacturonase
CAA40803	22424	PL	*	*Zea_mays*	PG1	1	polygalacturonase
P26216	129940	PL		*Zea_mays*	N.A.	1	EXOPOLYGALACTURONASE PRECURSOR
P35338	548492	PL	*	*Zea_mays*	N.A.	1	EXOPOLYGALACTURONASE PRECURSOR
P35339	548493	PL		*Zea_mays*	N.A.	1	EXOPOLYGALACTURONASE PRECURSOR
S16998	100912	PL	*	*Zea_mays*	N.A.	1	polygalacturonase
CAA40851	1360705	PL	*	*Zea_mays*	N.A.	1	polygalacturonase
S25824	283049	PL	*	*Zea_mays*	N.A.	1	polygalacturonase precursor (clone W2247)
S25825	283050	PL	*	*Zea_mays*	N.A.	1	polygalacturonase precursor (clone W2265)
S30064	629852	PL	*	*Zea_mays*	N.A.	1	polygalacturonase
BAB20805	12082296	PL		*Zinnia_elegans*	ZePG1	1	polygalacturonase
