# Supplementary material for: Patterns of expansion and expression divergence in the plant polygalacturonase gene family
Source: Genome Biol. 2006 Sep 29;7(9):R87. doi: 10.1186/gb-2006-7-9-r87 (PMC1794546; doi:10.1186/gb-2006-7-9-r87)
Supplement: Additional data file 2 — Table of assignment of PGs to duplicated blocks in Arabidopsis. [file gb-2006-7-9-r87-S2.pdf]

SUPPLEMENT B. Assignment of PGs to duplicated blocks

I. BWH blocks assignment – BWH block names used in Figure 3 correspond to the original names (BWH names) obtained from http://wolfe.gen.tcd.ie/athal/all_results [45]. Among the Arabidopsis PGs, 12 (shaded) are assigned as duplicated genes by Blanc et al. (2003). The rest of the PGs are assigned to BWH blocks if they are flanked by assigned duplicates.

Gene name	AGI block	BWH block	BWH names
At2g15450		24a	R_204067704020
At2g15470		24a	R_204067704020
At2g15460		24a	R_204067704020
At2g26620		24e	R_204153002470
At4g13760	34a2	34a	R_304185103260
At2g40310	23a2	23a	R_203257711080
At1g43080			
At1g43090			
At1g43100			
At1g17150		11b	R_101123907580
At1g78400			
At2g33160	12a2	12a	R_102031203980
At4g18180	45a2	45a	R_405128403640
At1g02790	14a1	14a	R_104000102440
At3g14040	13b2	13b	R_103352501880
At3g07850		35w	R_305052001580
At5g48140		35v	R_305052001580
At3g07830		35w	R_305052001580
At3g07820		35w	R_305052001580
At3g07840		35w	R_305052001580
At2g43870	23a2	23a	R_203257711080
At3g59850	23a1	23a	R_203257711080
At2g43860	23a2	23a	R_203257711080
At1g65570			
At2g43880	23a2	23a	R_203257711080
At2g43890	23a2	23a	R_203257711080
At1g05650	12a1		
At1g05660	12a1	12a	R_102031203980
At2g41850	23a2	23a	R_203257711080
At3g57510	23a1	23a	R_203257711080
At3g07970			
At1g80170	11b2	11b	R_101123907580
At1g02460	14a1	14a	R_104000102440
At4g01890	14a2	14a	R_104000102440
At1g48100	13a1	13a	R_103319703610
At1g56710		11a	R_101071703180
At3g26610		13a	R_103103800590
At5g14650	35b1	35b	R_305000103160
At1g10640	11a1	11a	R_101071703180
At1g60590	11a2	11a	R_101071703180
At5g39910		35x	R_305230002200
At3g15720	13b2	13b	R_103352501880
At5g17200	35b1	35b	R_305000103160
At4g35670	24a2	24a	R_204067704020
At5g27530		35w	R_305033201380
At5g44830	45a1	45a	R_405128403640
At5g44840	45a1	45a	R_405128403640
At1g80140	11b2	11b	R_101123907580
At4g32380	24x2	24e	R_204153002470
At4g32370	24x2	24e	R_204153002470
At1g23470	11d1	11b	R_101123907580
At1g23460	11d1	11b	R_101123907580
At1g70500	11d2	11b	R_101123907580
At1g19170	11c1	11b	R_101123907580
At3g42950		35y	R_305275700510
At4g33440		24a	R_204067704020
At2g23900		24e	R_204153002470
At3g48950		35z	R_305323600240
At3g61490	23a1	23a	R_203257711080
At4g23500	44a1	44a	R_404077901550
At4g23820	44a1	44a	R_404077901550
At5g41870	15a2	15a	R_105428301090
At3g62110	23a1	23a	R_203257711080
At3g16850			
At3g06770		35w	R_305052001580
At3g57790	23a1	23a	R_203257711080
