# Supplementary material for: Patterns of expansion and expression divergence in the plant polygalacturonase gene family
Source: Genome Biol. 2006 Sep 29;7(9):R87. doi: 10.1186/gb-2006-7-9-r87 (PMC1794546; doi:10.1186/gb-2006-7-9-r87)

SUPPLEMENT C

Phylogeny of fungal, bacterial, metazoan, and plant PGs

For all analyses in this study, the phylogenies were based on amino acid sequence alignments of GH28 domains. A simplified phylogenetic tree for eubacterial, and eukaryotic PGs is shown below (for lists of genes used in this analysis, see Supplement A, B). Interestingly, the PGs from eubacteria, plants, and fungi almost all clustered into their taxon-specific clades. The relationships between eubacterial, fungal, and the plant PGs suggest that the expansion of plant PGs occurred after the divergence of plants from the animal and fungal lineages. The plant PGs fall into three distinct groups: I, II, and III. Group I is likely more closely related to the Fungal Group II if this tree is rooted at mid-point. Group II is clustered with a eubacterial PG from *Thermotoga*, suggesting that either they shared a common ancestry or a horizontal transfer event occurred. Group III genes are embeded among several eubacterial genes. Interestingly, the polygalacturonases from the rice weevil (*Sitophilus oryza*) and the leaf-eating beetle (*Phaedon cochleariae*) share common ancestry with Fungal Group A; while the metazoan PG from *Meloidogyne incognita* (MI-PG-1) is more closely related to the eubacterial PGs.

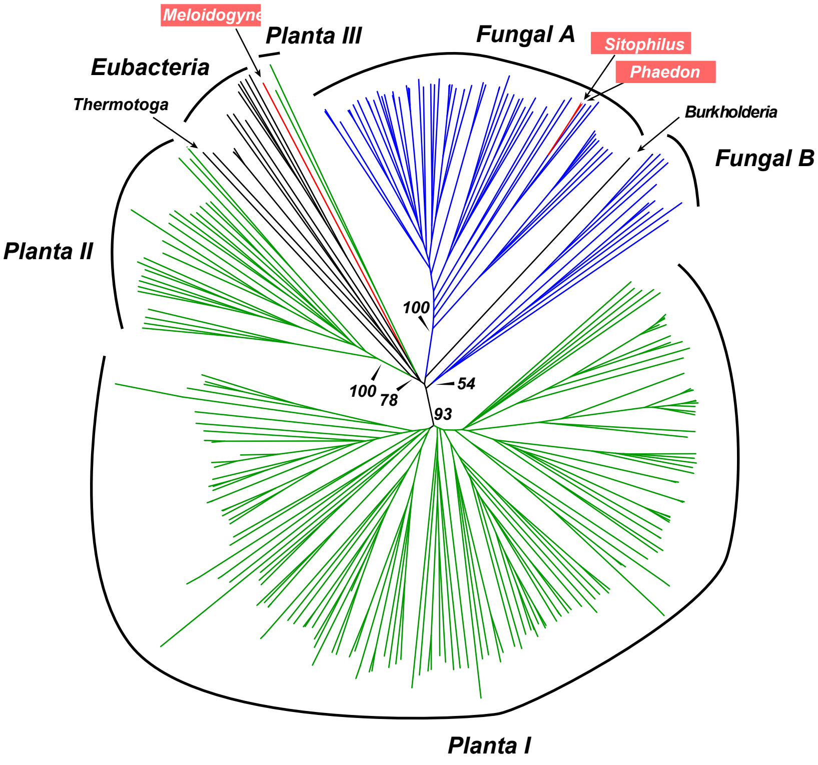

Supplement: Additional data file 3 — Figure showing a phylogeny generated with all the PGs from fungi, bacteria, metazoa, and plants. [file gb-2006-7-9-r87-S3.pdf]
