# Supplementary material for: Patterns of expansion and expression divergence in the plant polygalacturonase gene family
Source: Genome Biol. 2006 Sep 29;7(9):R87. doi: 10.1186/gb-2006-7-9-r87 (PMC1794546; doi:10.1186/gb-2006-7-9-r87)

# Supplement D. Matching areas for duplicated blocks containing PGs in both regions

The blocks that contain polygalacturonases (PGs) in both duplicated regions were further analyzed with BLAST to determine if the regions containing PGs were collinear. The sequence and annotation used is the 111203 version from MIPS.

The matching areas were identified by querying the nucleotide sequences of one of the duplicated regions against the translated database of the other region (tBLASTx). To increase the number of High Scoring Pairs (HSPs) recovered, the query sequences were splitted into 5kb windows to conduct the search. The default BLAST parameters were used. Two threshold cutoff were applied when parsing the BLAST output:

matching length - at least 50 amino acids
matching identity - at least 60%

There are 10 duplicated blocks and their names are shown on top either derived from AGI or BHW (with block name followed by "bhw"). For each block, the qualified matching areas are linked by a line with the color reflecting the identity level. The starting and ending positions of each region are shown at the edge of the chromosome regions. The genes located in the regions examined are drawn to-scale flanking the chromosomes. PG names are italicized and are indicated by arrows. A vertical bar is shown to delineate a 100kb region flanking each PG. The gene names in red that flank the PG containing regions were analyzed further.

Matching area and orientation (this example, 3'-5')

>= 90% identity

>= 80% identity

>= 70% identity

>= 60% identity

100 kb

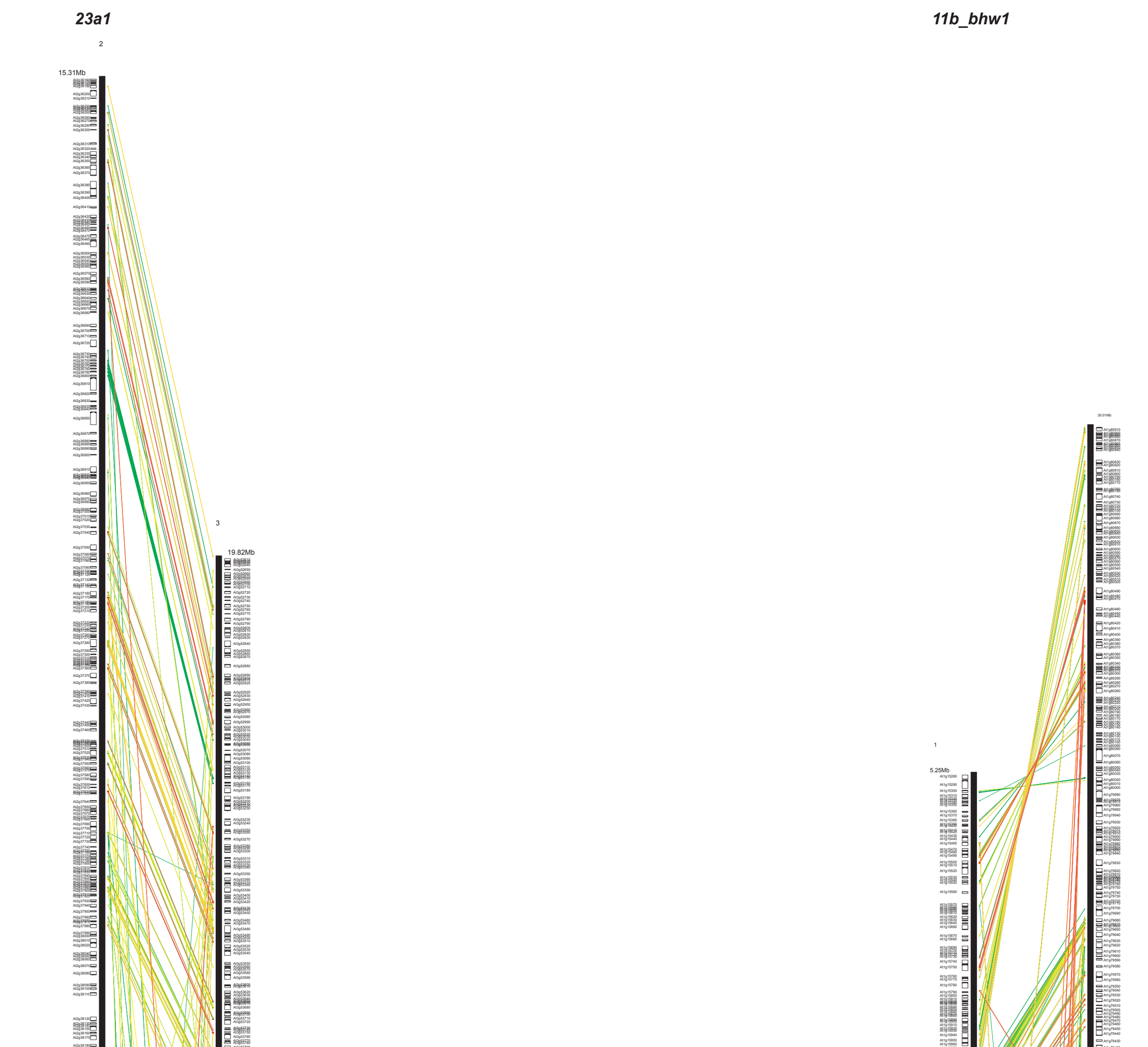


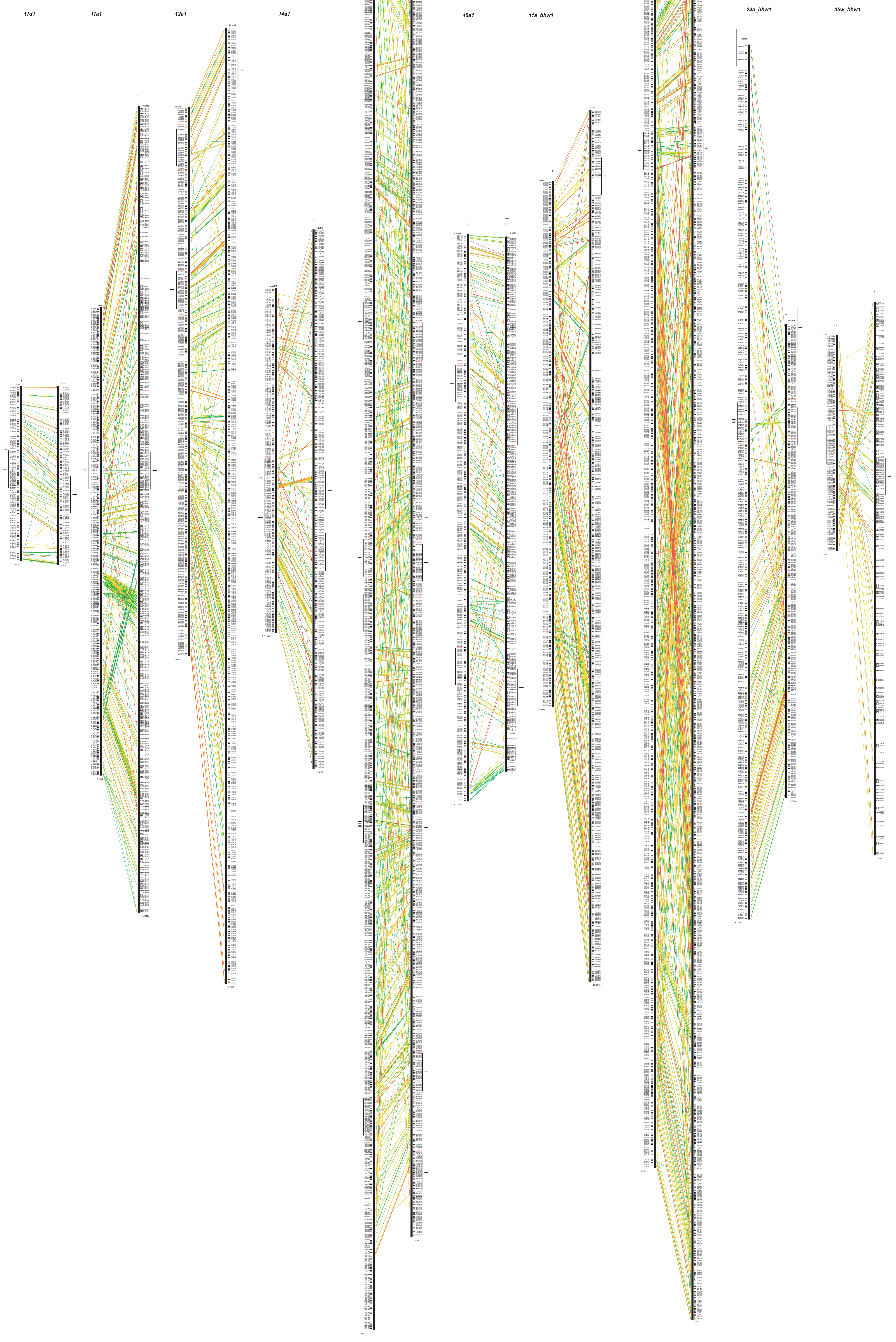

Supplement: Additional data file 4 — Figure with the matching areas for duplicated blocks containing PGs in both regions. [file gb-2006-7-9-r87-S4.pdf]
