# Supplementary material for: Patterns of expansion and expression divergence in the plant polygalacturonase gene family
Source: Genome Biol. 2006 Sep 29;7(9):R87. doi: 10.1186/gb-2006-7-9-r87 (PMC1794546; doi:10.1186/gb-2006-7-9-r87)
Supplement: Additional data file 5 — Primer pairs and conditions used to amplify PG genes. [file gb-2006-7-9-r87-S5.pdf]

Supplement E. Primer pairs and conditions used to amplify PG genes

Gene ID		Primer Pair Sequence	Annealing Temp. (℃)	# of Cycles
At1g02460	PG1F<br>PG1R	TGATTGTGTATCGATTGGCTCC<br>CATTCCAACAAAACGGATCCA	62	36
At1g02790	PG2F<br>PG2R	AAGATTGGGTCAACAATCCG<br>CAGACAGCTTCACCTTTCCCG	62	36
	PG2F-A<br>PG2R-A	GCTCCAACAACGTAACCGTCGAA<br>CGAAGATAACATTGGCGTTATCGCA	64	36
At1g05650	PG3F<br>PG3R	AACTCCGCAAAAGACGTGAT<br>GTTGGACGGACAATAGTTTTGG	62	36
	PG3AF<br>PG3AR	CAAAACGGCGTGAGGATAA<br>TCCAGTTAAAGCTTCTTTTCTCT	59	36
At1g05660	PG4F<br>PG4R	GAAGGAACTGAAAGAAGACGGAGTA<br>TCGGTTTAGACAACTAGTAGGTTGA	62	36
At1g10640	PG5F<br>PG5R	TGGGAGTTTTGGTGTGGAAGT<br>TGCTATGGTCACAGTAGAATTGG	55 (62)	36
At1g17150	PG6F<br>PG6R	GACCGGGACATGGAATTAGTA<br>ACAATGCGGAGGAACCATCTT	62	36
	PG6F-A<br>PG6R-A	TGTGTTCATGGCTTTTGGTTTGC<br>GGGACAATTATTGTCCTTCTGGCA	64	36
At1g19170	PG7F<br>PG7R	CGAGGATATGGTAATTGAGGA<br>TAGTCAATGTCCGCCACATT	62	28
At1g23450	PG8F<br>PG8R	GGTCTGGATGCCAAGGCTTTGTAT<br>AGTCTGGCAAAAACATCGAACA	62	36
At1g23460	PG9F<br>PG9R	GGTTTTGGAGTAAAGCAAAGA<br>CAACTTTTGTTCACATGGACC	62	36
	PG9F-A<br>PG9R-A	CCAGCAATTGGGACTCAAAGTTC<br>TCCATGTCCAGGTCCACAATAGAT	64	36
At1g23470	PG10F<br>PG10R	GCGAAAGCCATCAAATTTGC<br>TGTTGTTACATGAAGAAAGCA	62	36
	PG10F-A<br>PG10R-A	TCAGCCATATAATGTACGGAACA<br>CACACGTGACAGACATATCAAACAA	64	36
At1g43080	PG11F<br>PG11R	ATTGACGTTGGAAAACCG<br>CAATGAAAGTTGAGATTAGGA	62	36
At1g43090<br>At1g43100	PG12-13F<br>PG12-13R	TTGATCAGAAGTATTGCCCTCACC<br>GGGTAATGGAGGAAGCTCGA	62	36
At1g43100	PG13F-A<br>PG13R-A	CGAAAGGAAAATTCTACCTCGGTG<br>ATGTCCAGGACCACACTTGACGTT	64	36
At1g43080<br>At1g43090<br>At1g43100	PG11-13F<br>PG11-13R	CGTGAACAACTCAAGAATCAAAGAC<br>CCTTTCTCGAGTCCAGTATGCTTT	64	36
At1g48100	PG14F<br>PG14R	CCAGACGGATCATCATAGT<br>CGACAGAATTGTAAATGCCAA	62	36
At1g56710	PG15F<br>PG15R	TTGTATCGCCCGATGGAC<br>TGCGAATCTGAACATTGTGTG	62	36
At1g60590	PG16F<br>PG16R	AAACCGGTTGCTCCAATGTGT<br>TCCATTTCGCGCTGGCTTT	62	27
At1g65570	PG17F<br>PG17R	ATAGCAACGGGTTCGTGGAG<br>CGTGAAATTGTTGGCCCTTT	62	36
	PG17F-A<br>PG17R-A	CGCCGTTTGCTTAATACTTTGATTC<br>CAAGAACCGTAACATTGTGAACACC	64	36
At1g70500	PG18F<br>PG18R	CTCTGTTTGCTCTGTTGATGG<br>CATCGAATTTCGTCGCATTT	62	36
At1g78400	PG19F<br>PG19R	TCCGGGTTTTAGCTTAATTTTG<br>TCGCCAGTTGCTATGTCAA	62	36
	PG19F-A<br>PG19R-A	TCCAGATGATTGATGTCCAAAGCC<br>GGCGAGTGGAGATATCAACTTTTAC	64	36
At1g80140	PG20F<br>PG20R	GGAGGAACAGAGGTAACAGTGGA<br>AGCAGTGACCGGAAAAGGGA	62	36
	PG20F-A<br>PG20F-A	GGAATTGTCGATGATACATCTGTAA<br>ATTTGCCTTGGCCGTCCAA	61	36
At1g80170	PG21F<br>PG21R	TGAACAATGTGTCAAACCCCA<br>ATGCAGATGTGATCCCGGACT	62	27
At2g15450<br>At2g15460	PG22F<br>PG22R	CAATCGCTAATGATCTTTCATGTT<br>ACTTATCCTTTTGCAAGCACGG	62	36

Gene ID		Primer Pair Sequence	Annealing Temp. (^{o}C)	# of Cycles
At2g15470	PG22F-A<br>PG22R-A	ACCGTTCATAGAGACAAGCTAACTC<br>TAGGACAATTGGGGTTTGTGTG	62	36
At2g23900	PG25F<br>PG25R	TGAAGTATCGCAGTGGTGGATTT<br>TGAAAGGAATCGTTCTTGAACCC	62	36
At2g26620	PG26F<br>PG26R	TTGGTATATGACAGAATGTGATTG<br>GTTAGGATTTGCAGGCGCTAAT	62	36
	PG26F-A<br>PG26R-A	CTCTCTGGAACCACTAAACTCAATA<br>TTCTTAGCTCTAAATGTTCAACTGC	61	36
At2g33160	PG27F<br>PG27R	AGATAGCCCTAATACCGACGGAAT<br>CATGAGAAGCATACTTTCCAGGAC	62	36
At2g40310	PG28F<br>PG28R	CAGCTTCAAAAATCCTCGTTTCG<br>TCTTCAGATGAACCGGATTTATTG	62	36
	PG28F-A<br>PG28R-A	GAGATGCTAACTCTATATAAGCTGA<br>GTCATTGAGTGGCCAAGATTGT	56	36
At2g41850	PG29F<br>PG29R	CCAAAAACGACAGCATTCCT<br>CAGCTCCATTTGAAGAACATGC	62	36
At2g43860	PG30F<br>PG30R	CTACTGTCTTCATAAACCTTGCCCT<br>AGTCATGGAGCAATCGGGAC	60 (62)	36
	PG30F-A<br>PG30R-A	CCCATCTCATGTACCTTCTTCTACA<br>CTGGCTGTTGATCGACGTTAAA	61	36
At2g43870	PG31F<br>PG31R	ACTACTGTCCTGATCACGATTGTC<br>CTCCCTCCATGATTTACGAAA	62	36
At2g43880	PG32F<br>PG32R	TAATGGAAAAGGTTGTCCTCGC<br>CGGATGAATCTAAGGTTATAAAAAC	62	36
At2g43890	PG33F<br>PG33R	ACTGCCCTAGTAATCAAGGTTGTC<br>AACTCGATCACAGAAGGCAA	62	36
At3g06770	PG34F<br>PG34R	TTGGCGCGAAAGTTCTTCA<br>GCTTGTTACCGTTGATCAAACTCT	62	36
At3g07820	PG35F<br>PG35R	CCTCATCGACCAAGAGTACTGCCC<br>TGCGGATTTGCCAAACATACATG	62	36
	PG35F-A<br>PG35R-A	CATGCCAATCTTCGTCACCAA<br>CTTGAAGAGTGCAGTTGATGACCTT	64	36
At3g07830	PG36F<br>PG36R	GTTCAAAATACGAGACGTGTAATT<br>CTTCCTCGGTGTCGGAGATT	60 (55)	36
	PG36AF<br>PG36AR	CATTTTGAGAATATCATCCTTAGA<br>CAAGAAATTGATTGGAAAAAGA	62 (55)	36
At3g07840	PG37F<br>PG37R	ACGGCTAAAAATTATACGAATGT<br>ACCAAGCTTGAACTCACCCTTT	60 (55)	36
	PG37AF<br>PG37AR	CCGCCTCTGGTATTCATTTTGA<br>GATTGAAACATTCTTCATTGCCAA	62 (55)	36
At3g07850	PG38F<br>PG38R	TCACGTATAGGCCCAATTAGATAA<br>CACAAGCTTCTTTCCATGCAG	62	36
	PG38F-A<br>PG38R-A	ACAATGTTAGCCTTCCCGTTCTCA<br>GCGAATCCACACAACAATTACTACC	64	36
At3g07970	PG39F<br>PG39R	AAAGCTTGGGAAGCAGCTTGT<br>TTTGCAAGATTTTGGCCACC	62	36
At3g14040	PG40F<br>PG40R	TAGCCTTCCCGTTCTCATCGACCA<br>TGCAATGAATGTTGGGTGCAACAA	62	36
	PG40F-A<br>PG40R-A	ACCCAAAACTATTGAGACTTAAACTTC<br>ACCAAATACTCACCTTTTGGCAC	60	36
At3g15720	PG41F<br>PG41R	GGGAAATGGAACCAAAAACATC<br>ACAAAAACAGGGGTGGATTTGC	65	36
	PG41F-A<br>PG41R-A	TTCTTCACATTGTCTTCCCCTG<br>AACGACAAACTGCCCATCTCCT	62	36
At3g16850	PG42F<br>PG42R	GCAATAATGCCATCTGCAAGGA<br>TCAAACTTCTGTACCGCTTTCCA	62	36
At3g26610	PG43F<br>PG43R	TGGAAGGCAGCTTGTAAAGTG<br>TTGGTTTCATTGGTGGGAT	62	36
At3g42950	PG44F<br>PG44R	GACGTTTCCTCTCTTCTCACAAA<br>CAACATCTCTAAGATTCTGTCCA	62	36
At3g48950	PG45F-A<br>PG45R-A	CCGTACTTGATCGAGATCTTGTTC<br>TCGAGACTCGCTGACATTGTAACA	62	36
At3g57510	PG46F<br>PG46R	AGGACAAATGCGAAGACCAA<br>AAGAAGCAATGCATGTAGTACAA	62	27
At3g59850	PG47F<br>PG47R	TGGCAACGAAAATTGCCCTA<br>CCGTAATATATATGAAAATACGACG	60(62)	36

Gene ID		Primer Pair Sequence	Annealing Temp. (^{o}C)	# of Cycles
At3g61490	PG48F-A<br>PG48R-A	ACGGCAAGACGCTAAACACAAA<br>ACGGGCGAGTGTATTTAGCTT	62	36
At3g62110	PG49F<br>PG49R	TCCTGGTACAGGCCTTCTATG<br>TCTTCTACCGGGCAATTCTCTT	62	36
At4g01890	PG50F<br>PG50R	TAGGACCCGGAGCATACGATACGATA<br>GGATTCGATTTGAGACACGAG	62	36
At4g13760	PG51F<br>PG51R	TTAGTGTCGGAAGCTTAGGGAA<br>TCTATGCTCGAACTATTTTCTAG	62	36
	PG51F-A<br>PG51R-A	GCATCCCAATTTCAGTTGTT<br>ATCAAGTGTACCGGAACCAGA	60	36
At4g18180	PG52F<br>PG52R	AGGGAGATATCCAAATGAGAAAG<br>CGTTAAACACGAACCAAATTTAA	62	36
	PG52F-A<br>PG52R-A	GGCGGTAAAAGAAACGGTTAACT<br>CAAGGACCAGAGAATCTGAGAGAA	62	36
At4g23500	PG53F<br>PG53R	TTTCTCCTGATAGTGCCGTGA<br>TCCCGATATTCCAGCTAACTGA	62	36
At4g23820	PG54F<br>PG54R	GTCATCCATCACGTTACGATCTTAG<br>TAGTTCTCAGCTCCGGACACG	62	36
At4g32370	PG55F<br>PG55R	TCGGGGTCGCCAAAAATATA<br>CGCTGGAGGTTTAGCATAGTATACA	62	36
At4g32380	PG56F	TTCCCCATCATCATCGATCA<br>GCTCACATCAGGAAACAATATTAC	62	36
At4g33440	PG57F<br>PG57R	AATGTGGTGGGAGTTATGGTGGAA<br>TCGTCTGAGTGATCATTTGAGCCA	62	36
At4g35670	PG58F<br>PG58R	TACGGACATCACACTCGTTAACAC<br>GGCAGCAAATTCTGTGACTGTA	62	36
At5g14650	PG59F<br>PG59R	TCAATTCAAACGGGATGCTCG[1]<br>TGTCTTCAGTTCACCGTGTGC[1]	62	36
	PG59F-A<br>PG59R-A	TGCTCTGTTCTAAACGAAAGTAAT<br>AGGAAGGAAACAGGTCCAACAA	58	36
	PG59F-B<br>PG59R-B	CGTAAGTAAAGATGTTGATGTCAATT<br>CGATTTTCTCTTTACATTCTTTTCCC	60	36
At5g17200	PG60F<br>PG60R	TCGTTTGCGATTTGTTAAATACG<br>AGACTTGCATGGACCGATGAAT	62	36
At5g27530	PG61F<br>PG61R	TGGACAAGGTTATGCAAGAAACA<br>CCGCCACTGAAAGACTATTACTAT	62	36
At5g39910	PG62F<br>PG62R	GATGCTCCAGTTTATTCTCCT<br>ACAAGGGCCATGAAACTCAA	58	36
	PG62F-A<br>PG62R-A	CCAATCCGTTACATACAGTCATACA<br>GTGTCTTCCCATGCATCTTTGA	62	36
At5g41870	PG63F<br>PG63R	GACCGTTTTAAGCTAAGAGAAGG<br>GCTGATATATCTTCGTCCAGGA	61	36
	PG63F-A<br>PG63R-A	CAAACTGTAGTTATGGGCTTGC<br>ATCTTCGTCCAGGATGCTCC	63	36
	PG63F-B<br>PG63R-B	TTTCTTCGTCCGTGCCATTACT<br>TGAGTCCGTCTCCGTGAATAAA	62	36
At5g44830	PG64F<br>PG64R	CTCCATTCTGTTCTTGTAAATGGC<br>TTGTTCCCGAACCATTGACT	62	36
	PG64F-A<br>PG64R-A	CATCAGCACCCAATGGTACAT<br>CGACCAAGCTGCTTTGTTAA	60	36
At5g44840	PG65F<br>PG65R	GGGATACGCAAAAAATATAC<br>CTATCGGTACAACTCTTCAGT	53	36
	PG65F-A<br>PG65R-A	CATTTTAGTTTTCAATGTCAATGGC<br>GTAATACCATTTCCATCGGCAC	62	36
At5g48140	PG66F<br>PG66R	ATCGTGATGATGGGTCCATGCAA<br>ACAAGCAGCAGAAGGCCATGTCTT	67	36
**At3g57790**	**PG67F**<br>**PG67R**	**TGATGCAATCTGTCCCAAGA**<br>**TCCGTTTGGTGAACCTGATA**	**62**	**36**
	Oligo(T) 18	TTTTTTTTTTTTTTTTTT		

[1]amplifies two bands from cDNAs

Unless indicated otherwise, annealing temperature was 62°C and 36 cycles of PCR was carried out.
