# Supplementary material for: Patterns of expansion and expression divergence in the plant polygalacturonase gene family
Source: Genome Biol. 2006 Sep 29;7(9):R87. doi: 10.1186/gb-2006-7-9-r87 (PMC1794546; doi:10.1186/gb-2006-7-9-r87)
Supplement: Additional data file 6 — Table showing summary of expression tags and list of cDNAs for Arabidopsis PGs. [file gb-2006-7-9-r87-S6.pdf]

SUPPLEMENT F. Summary of expression tags

I. List of cDNAs for Arabidopsis PGs

A list of cDNAs accession numbers were the SIGnAL database [49]. The accessions were used to retrieve cDNA sequences from GenBank. A BLAST search was conducted with Arabidopsis PG predicted cDNA sequences as queries and SIGnAL cDNA sequences as subjects. The top matches were inspected and assigned to Arabidopsis PGs.

Gene name	Count	Accession
At4g33440	2	BT002785, BT004354
At3g62110	2	AF324992, BT000724
At3g06770	2	AY093030, AY128935
At1g02790	3	AY065210, AY133812, AF428425
At4g23500	1	AY142497
At5g14650	1	BT005780
At1g10640	2	AY039852, AY143959
At1g80170	2	AY046002, AY142668
At2g41850	1	AY078936
At4g23820	2	AY128793, AY062697
At1g19170	2	BT000031, AY081277
At5g48140	1	BT009687
At3g42950	2	AY050383, AY143967
At3g14040	2	AY114635, AY062447
At3g57510	1	BT005376
At3g07820	2	AY096534, AY065239
At3g07830	1	BT005245
At3g16850	2	AY035074, AY113876
At1g60590	2	AY060568, BT000611
At1g48100	3	AY096360, AY050798, AF410319
At3g57790	4	AY065080, BT003366, AY136461, BT001251
