# Supplementary material for: Patterns of expansion and expression divergence in the plant polygalacturonase gene family
Source: Genome Biol. 2006 Sep 29;7(9):R87. doi: 10.1186/gb-2006-7-9-r87 (PMC1794546; doi:10.1186/gb-2006-7-9-r87)
Supplement: Additional data file 7 — A list of PGs that are expressed in the floral organ abscission zones of Arabidopsis with their patterns of expression. [file gb-2006-7-9-r87-S7.pdf]

Supplement G. Patterns of gene expression during abscission

Gene	Clades	Cluster	Block
7B			
At2g 43870	A3	2b	23a
At1g19170	B8		11c
At2g23900	B6		24e’
At3g06770	B1		
At3g42950	B8		35y
At3g48950	B6		35z
At4g33440	B4		24a’
At5g48140	A1d		35v
7C			
At2g43860	A3	2b	23a
At1g70500	A14		11d
At2g 33160	A1a		12a
At3g07820	A1d	3a	35v
At3g26610	A9		13w
At4g32370	A2	4a	24e
At5g27530	A5		35w
7D			
At1g02460	A11		14a
At1g56710	A10		11a’
At3g61490	B6		23a
7E			
At1g10640	A6		11a/11a’
At1g23460	A14	1b	11d
7F			
At1g80170	A4	1d	11b
At3g16850	B1		
At4g23820	B3		44a
At1g02790	A1d		14a
At1g48100	A13		13a
At1g60590	A6		11a
At3g07850	A1d	3a	35v
At3g62110	B2		23a
At4g23500	B6		44a
At3g57510	A15		23a
7G			
At1g05650	A3	1a	12a

At1g05660	A3	1a	12a
At1g65570	A3		
At4g35670	A5		24a
At5g17200	A5		35b
At5g41870	B3		15a

7H

At3g59850	A3		23a

7I

At2g41850	A15		23a
At2g43890	A3	2b	23a
At2g43880	A3	2b	23a
At3g07970	A15		

7J

At4g01890	A11		14a
At4g32380	A2	4a	24e
