# Supplementary material for: Patterns of expansion and expression divergence in the plant polygalacturonase gene family
Source: Genome Biol. 2006 Sep 29;7(9):R87. doi: 10.1186/gb-2006-7-9-r87 (PMC1794546; doi:10.1186/gb-2006-7-9-r87)
Supplement: Additional data file 8 — GH28 domain sequence alignments of rice and Arabidopsis PGs analyzed. [file gb-2006-7-9-r87-S8.doc]

**Supplement H. Arabidopsis and rice sequences used for alignment**

>At1g02460

-------NNTDSVLLVPYGY-------TFMIQSTIFTG-PCRSYQFF-------------

----------------------------------QVDGTIVTPDGPESWP-SNISKRQWL

VFYRVNGMALKGE---GVIDGRG--------QKWWDLPCKPHRSVNKSAI----------

------------VTGPCDSPIALRFFMSSN-----------------------------L

RVEGLQIKNSPQFHFRFDGCQGVHVES-LHITAPPLSPN-TDGIHIENSNSVTIYNSIIS

NGDDCVS-------------------------------------IGSGSYDVDIRNLTCG

PGGHGISIG------SLGNHN--SRACVSNITVRDSVIKYSDNGVRIKTWQGGSGSVSGV

T---------FNNIHVDSVRNPIIIDQYYCMTKD-------------------CANKTS-

-----------------AVFVSDIAYQGIKGTYDIRS---PPMHFGCSDAVP-CTNLTLS

DIELLPA---------------------KGEIVLDPFC----------------------

>At4g01890

---SNNKNNTASVLHVPYGF-------TFMIRSTIFTG-PCRSYQYF-------------

----------------------------------QVDGTIVPRDGPKSWP-SSLNKRQWL

AFYRINGMALQGA---GVIDGRG--------QNWWDLPCKPHQ-----------------

---------------------ALRFFMSSN-----------------------------V

IVKGLSIKNSPQVHLKLDGCHVVHINS-LRIISPPASPN-TDGIHIENSNSVEIYNSVIS

NGDDCVS-------------------------------------IGPGAYDIDIRNITCG

PGGHGISIG------SLGEKN--SHACVSNVTVRDSFIKFSENGVRIKTWQGGSGSVSGV

T---------FDNIHVDTVRNPIIIDQYYCTTKS-------------------CANKTS-

-----------------AVFVNDIVYQSIKGTYDIRS---PPMHFGCSNNVP-CTNLTLS

NIELLPS---------------------KEDIVVGPFC----------------------

>Osi000190.10

------ADDGAGVVLAAAGR-------SFLIHTTVFTG-PCQGSVTL-------------

----------------------------------QVDGTIVAPSEPATWP-AN-NKRNWL

VFYRADGVSLVGA---GLIDGKG--------QKWWDLPCKPHKGGN--------------

------------THGPCDSPVAMRFAISNN-----------------------------V

TVRGLKVQNSPEFHFRFDNCNGVRVDG-LSISSPALSPN-TDGIHVENTQDVLITNTVVS

NGDDCVS-------------------------------------IGAGTLNVHIENVTCG

PG-HGISIG------SLGKAG--TKACVANVTVRNAVIRHSDNGVRIKTWQGGSGSVSAV

A---------FENVRMDAVRNPIIIDQYYCLSKS-------------------CENETT-

-----------------AVFVNGVSYSGIRGTYDVRG---PPIHFGCSDAVP-CTNITLS

DVELLPA---------------------SGDTVDEPFCWNVYGNAATPTVPPVS------

>Osi007050.2

-------AVESAVISVPSDG-------TFTITTTTFTG-PCKPGLVF-------------

----------------------------------QVDGVLMPPDGPDCWP-PSDNRRQWL

VFSNLDGLTLRGA---GTIEGNG--------EGWWNLPCKPHRGPNGST-----------

------------LRGPCDSPTLVRFFMSRN-----------------------------L

VVEGLRVENSPEFHFRFDGCSDVRVDG-LSIRSPANSPN-TDGIHVENTQRVAIYNSMIS

NGDDCIS-------------------------------------IGTGSYDVDIQNVSCG

PG-HGISIG------SLGVHN--SQACVANVTVRNAVIRNSDNGLRIKTWQGGMGSVSGI

N---------FDTVSMENVRNCIIIDQYYCLDKR-------------------CMNQST-

-----------------AVHVTDVSYANVRGSYDVRA---APIHFACSDTVP-CTNITMS

EVELLPF---------------------SGELVDDPFC----------------------

>Osi031779.1

-------AVESAVISVPSDG-------TFTITTTTFTG-PCKPGLVF-------------

----------------------------------QVDGVLMPPDGPDCWP-PSDNRRQWL

VFSNLDGLTLRGA---GTIEGNG--------EGWWNLPCKPHRGPNGST-----------

------------LRGPCDSPTLVRFFMSRN-----------------------------L

VVEGLRVENSPEFHFRFDGCSDVRVDG-LSIRSPANSPN-TDGIHVENTQRVAIYNSMIS

NGDDCIS-------------------------------------IGTGSYDVDIQNVSCG

PG-HGISIS------LFL------------------------------------------

------------------------------------------------------------

------------------------------------------------------------

------------------------------------------------------------

>At1g48100

-------AVESGVVLAPEGG-------VFKITSTIFSG-PCKPGLVF-------------

----------------------------------QLDGVLMPPDGPEEWP-EKDNKNQWL

VFYRLDGFTFSGK---GTVEGNG--------QKWWDLPCKPHRGPDGSS-----------

------------SSGPCASPTMIRFFMSNN-----------------------------I

EVKGLRIQNSPQFHMKFDGCQGVLINE-IQISSPKLSPN-TDGIHLGNTRSVGIYNSVVS

NGDDCIS-------------------------------------IGTGCSDVDIQGVTCG

PS-HGISIG------SLGVHN--SQACVSNITVRNTVIRDSDNGLRVKTWQGGTGSVSNL

L---------FENIQMENVLNCIIVDQYYCQSKD-------------------CRNETS-

-----------------AVKVFDVEYRNIKGTYDVRS---PPIHFACSDTVA-CTNITMS

EVELLPE---------------------EGELVDDPFCWNAYGKQETLTI----------

>Osi010090.2

-------SAESATVLVPSDG-------VFTITSTIFAG-PCKPGLTF-------------

----------------------------------QIDGVLMPPDGPASWP-AADGRRQWI

VFYRADGMTLSGK---GTIEGNG--------EEWWNLPCKPHRGPNGST-----------

------------LPGPCESPALIKFVASSD-----------------------------V

SVQGLRMENSPQFHLKFDGCSRVLVDG-LVVSSPASSPN-TDGVHVENTSSVRILNSRIS

NGDDCVS-------------------------------------IGGGCSGVRVENVTCV

HG-HGISIG------GLGARG--ARACVSNVTVRGARVVDSDNGVRIKTWQGGAGSVSGV

V---------FDAVQMVNVRGCIVIDQYYCDAHGGA--------------GAGCANQTA-

-----------------AVRVDGVAYRGIRGTYNPRGGGGAPVRFACSDTVA-CTGITMT

DVELLPAGGG-----------DEGGGASAGAKLADPYC----------------------

>At1g56710

----MSTGPKSALLLVPYTF-------CFLVKPTTFNG-PCRTNLVL-------------

----------------------------------QIDGFIVSPDGPRSWP-SN-YQRQWM

MFYRVNGLSIQGS---GVINGRG--------QKWWNLPCKPHKGLNGTT-----------

------------QTGPCDSPVAIRLFQSSK-----------------------------V

RIQGINFMNSAQFHVRFDNCSDVVVDS-VIIKAPASSPN-TDGIHIENTHNVQIRNSMIS

NGDDCIS-------------------------------------IGAGCFNVDIKNVTCG

PS-HGISIG------SLGVHN--SQAYVSNITVTNSTIWNSDNGVRIKTWQGGSGSVSRI

V---------FSNILMVNVRNPIMIDQYYCQTNN-------------------CANQTS-

-----------------AVIISDVLYANIKGTYDLRS---PPIHFGCSDSVP-CTNLTLT

EVDLFPS---------------------KGQHLENPFCWNAYGSMKITVPPVYC------

>At1g10640

-------KIEASVMLVPPEY-------TYLVGPISFSGPYCQANIVF-------------

----------------------------------QLDGTIIAPTDSKTWG-KG--LMWWI

DFTKLKGIKVQGK---GVIDGRG--------SGWWQQDSP---FIDSDTKLIVPLNNSAN

QNPPMPIRSELDERMPSIKPTALRFSGSFG-----------------------------V

EVTGITIQNSPQCHLKFDDCVGVVVHD-IAVSSPGDSPN-TDGIHLQNTKDVLIHSTTLA

CGDDCIS-------------------------------------IQTGCSNVFVHNVNCG

PG-HGISIG------SLGKEG--TKACVSNITVRDVAMHNTMTGVRIKTWQGGVGSVKGI

I---------FSNIQLNQVQIPITINQFYCDH-------------------SKCKNQTS-

-----------------AVAVEGVTYERIKGTYTVKP-----VHFACSDNFP-CVDVQLS

SIELKPVQE--------------------KYRMYDAYC----------------------

>At1g60590

-------KVEASTMIIPPDY-------IFLVGPISFSGPYCQANIVF-------------

----------------------------------QLEGMIVAPTDTESWG-GG--LMWWI

EFTKLSGITIQGN---GVIDGRG--------TVWWQQDYLSDYPIDDDFKLIVPLNNSVQ

ERPPMPIRSELNWRMPSIKPTALRFYGSID-----------------------------V

TVTGITIQNSPQCHLKFDNCVKVLVHD-VNVSSPGDSPN-TDGIHLQNTRDVMIHTTTLA

CGDDCIS-------------------------------------IQTGCSNVYVHNVNCG

PG-HGISIG------SLGKDS--TKACVSNITVRDVVMHNTMTGVRIKTWQGGIGSVKGI

L---------FSNIQLTEVQLPIVIDQFYCDH-------------------SKCMNHTS-

-----------------AVSVEGVTYEKIRGTYTVKP-----VHFACSDSFP-CIDVQLS

GIELKPVQL--------------------QYHMYDPFC----------------------

>Osi001448.1

-------KQGASTVVVPSEL-------EFLVGPISFSGPYCKPNILF-------------

----------------------------------QLDGTIVAPTSAKAWG-SG--LLQWI

EFTKLNGVSIQGN---GIINGRG--------QQWWTYSDIDDDEDDDT-QYDVEF-----

------------ERMPQVKPTALRFYGSFN-----------------------------V

VVAGITIVNSSQCHLKFDSCQGVMVHD-VTISSPENSLN-TDGIHLQNSKDVSIHHTNLA

CGTTSITKLKQN----------------IEPI----------------------------

------------------------------------------------------------

------------------------------------------------------------

------------------------------------------------------------

------------------------------------------------------------

>Osi004292.2

-------KVEASTVLVPSEL-------EFVVGPISFSGPYCKPNILF-------------

----------------------------------QLDGTILAQTSTRVWG-SG--LLQWL

EFTKLSGISIQGS---GVINGRG--------QEWWTYSDPNDDDNDDVDAYNVEL-----

------------EKMPQIKPTALRFYGSSN-----------------------------V

TVTGITIVNSSQCHLKFDSCQGVMVHD-LTISSPENSPN-TDGIHLQNSKQVSIHHTNLA

CGNALINSIKAKPTGFRTKGKLKTLVQVSEVIFALCDAGDDCVSIQTGCSDINIHNVNCG

PG-HGISIG------GLGRYN--TKACVSNVTVRDVNMFKTMTGVRIKTWQGGSGLVQGI

R---------FSNIQVSEVQTPIIIDQFYCDR-------------------TTCRNQTS-

-----------------AVAVSGVQYENIRGTFTIKP-----AHFACSDSSP-CSEITLT

GIQLKPLIVP-------------------QYHLYNPFC----------------------

>Osi013606.1

---------TGPCLGLPTG----------MLGRPGMTDPARHDG----------------

----------------------------------SLDGTIVAPTSAKAWD-SG--LLQWI

EFTKLNGVSIQGN---GIINGRG--------QQWWTYSDTDDDENDDT-QYDVEF-----

------------ERMPQVKPTALRFYGSFN-----------------------------V

VVAGITIVNSSQCHLKFDSCQGVMVHD-VTISSPENSLN-TDGIHLQNSKDVSIHHTNLA

CGDDCVS-------------------------------------IQTGCSNINIHNVNCG

PG-HGISIG------GLGRDN--TKACVSNVTVRDVNMFRTMTGVRIKTWQGGLGLVQDV

R---------FSNIQVSEVQTPIIIDQFYCDE-------------------RTCSNQTS-

-----------------AVAVSGVQYENIRGTFTIKP-----VHFACSDSSP-CSGITLT

GVQLRPVQIS-------------------HYRLNNPFC----------------------

>At5g14650

-------KVAASTLLVPSGS-------TFLVGPVSFLGKECKEKIVF-------------

----------------------------------QLEGKIIAPTSASAWG-SG--LLQWI

EFKALQGITIKGK---GIIDGRG--------SVWWN-------DMMGT------------

-------------KMPRTKPTALRFYGSNG-----------------------------V

TVSGITIQNSPQTHLKFDNCISIQVSD-FTTSSPGDSPN-TDGIHLQNSQDAVIYRSTLA

CGDDCIS-------------------------------------IQTGCSNINIHDVDCG

PG-HGISIG------GLGKDN--TKACVSNITVRDVTMHETTNGVRIKSWQGGSGSVKQV

M---------FSNIQVSNVANPIIIDQYYCDG-------------------GGCHNETS-

-----------------AVAVSNINYINIKGTYTKEP-----VRFACSDSLP-CTGISLS

TIELKPATG--------------------KASSLDPFCWKAHGELKTKTLPPIQ------

>Osi010408.2

-------AVRSSTVVVPAGY-------RFVVGPVTFTGDSCQPNTVF-------------

----------------------------------QLDGTIVANTDSGAWC-SGNAVQQWL

EFRSCTGLTIQGS---GTVDGQG--------SHWWSGGAPATDIDADR------------

-------------VGTNNRPTALRVYESTN-----------------------------V

AVTGITIQNSARFHLTFDTCRAVEVRG-VAIRSPGDSPN-TDGIHLAGSVGVSIQNATVA

CGDDCVS-------------------------------------IQDGCSRVLVRGVTCG

PG-HGISIG------GLGKGG--AMAFVSDVTVQDVFLVGTSAGVRIKTWQGGSGSVRGV

L---------FSGVRVSAVKTPIVIDQYYCDH-------------------ATCANQTA-

-----------------AVAVSGVAYSGITGTYTQRP-----VYLACSDAAP-CAGLRLE

DIKLAPVKDG-----------G-------YGRLYGPFC----------------------

>Osi004161.2

---------------------------DGMLQCSHEEH-QLQSRTWN-------------

----------------------------------QIDGDVLAPPGMGYWP-KARRPLQWL

NFKWLDGFTIQGT---GTVDGQS--------TLLRSVSP-ANVSQHWY------------

--------------VSGVKPTLIRFYSSFN-----------------------------V

SVRNIRITNSPQCHLKFDSSGGIKVKN-ITISSPGDSLN-TDGIHLQNTRDVDIRSSSIG

CGDDCIS-------------------------------------IQTGCSNVHMKNINCN

PG-HGISLG------GLGKDN--SLACVSDVFAEHINVENALYGVRIKTWQGGKGTVRNV

T---------FSNVRVANVATPIAIDQFYCDAGGG---------------GARCGNRSD-

-----------------AVGITGVAYRRVAGTYTYQP-----VRLACSDARP-CTGVSMA

DVRLSPASAT-----------G-------AGGLRQPLCW---------------------

>At3g26610

-------KVVGGKVEIPAGT-------QFLVKAVTLQG-PCKEETVV-------------

----------------------------------QIEGILVAPEKIGSWP-NSS-LFQWL

NFKWVSHLTIQGS---GTLNGRG--------YNWWNLDTYQTQTRNKY------------

--------------IPPMKPTALRFYSSNN-----------------------------V

TVRDISIVNSPLCHLKFDDSDGVKVNN-ITISSPENSPN-TDGIHLQNTRNVEIQHSNIA

CGDDCVS-------------------------------------IQTGSSNVHIHHINCG

PG-HGISIG------GLGKDK--SVACVSDIIVEDISIQNTLAGVRIKTWQGGLGVVKNL

T---------FSNIQVKDVKVPIVIDQYYCDK-------------------SKCKNQTR-

-----------------AVSISGVKYNNIVGSFTVQP-----VRIACSNNVP-CMDVDLM

DIRLRPSGGI-----------RG----LQTHQQQQALCWNSYGKTQGPLVPSSI------

>At1g23460

-------STSKSVFLVPEGR-------RYLVNATKFNG-PCEQKLIIQVK----------

-------------------L-------------SSIDGTIVAPDEPSNWD--SKFQRIWL

EFSKLKGVVFQGK---GVIDGSG--------SKWWAASCKKNKSN---------------

---------------------ALTIESSSG-----------------------------V

KVSGLTIQNSQQMNFIIARSDSVRVSK-VMVSSPGDSPN-TDGIHITGSTNVILQDCKIG

TGDDCVS-------------------------------------IVNASSNIKMKNIYCG

PG-HGISIG------SLGKDN--TTGIVTQVVLDTALLRETTNGLRIKTYQGGSGYVQGI

R---------FTNVEMQDVANPILIDQFYCDSPTT------------------CQNQTS-

-----------------AVKISQIMYRNITGTTKSAKA----IKFACSDTVP-CSHIVLN

NVNLEGNDG-------------QVEAYCNSAEGFGYGVIHPS------------------

>At1g70500

-------SAPNSVLLVPQGR-------SYLVNATKFDG-PCQEKLIIQ------------

-----------------------------------IDGTIIAPDEPSQWD--PKFPRNWL

QFSKLQGVVFQGN---GVIDGSG--------TKWWAASCKKNKSNPCVGAPT--------

---------------------ALTIYSSSN-----------------------------V

YVRGLTIRNSQQMHLIIQRSTTVRISR-VMVTSPGDSPN-TDGIHITASTDVVVQDSKIS

TGDDCVS-------------------------------------IVNGSAKIKMKRIYCG

PG-HGISIG------SLGQGH--SKGTVTAVVLETAFLKNTTNGLRIKTWQGGNGYVKGV

R---------FENVVMQDVANPIIIDQFYCDSPST------------------CQNQTS-

-----------------AVHISEIMYRNITGTTKSSKA----INFKCSDAVP-CSHIVLN

NINLEGNDG-------------KVEAYCNSAEGFGYGVVHPS------------------

>At1g23470

--------------FVKFLF-------SNLVSFDTFEE-IGNDVAVIVF-----------

-----------------------------------LEHSFTRSHEKR------HVCRESL

LLSSCNNKLSRIH---GLSERIS--------KRYQKPSR---------------------

---------------------------GDI-----------------------------V

VIRSPENPNKTPIKRVVG-VEGDCIS---FVIDPVKSDE-SQTIVVPKG-HVFVQG----

----------------------------------------------DYTHNSRDSRNFGP

VP-YGLIQGR-----VLWRDN--TTGIVTQVVLDTALLRETTNGLRIKTYQGGSGYVQGI

R---------FTNVEMQD---------------------------------------TS-

-----------------AVKISHIMYRNIT---KSAKA----IKFACSDTVP-CSHIVLN

NVNLEGKDG-------------QVEAYCNSAEGFGYGVYS--------------------

>Osi002228.5

-------SLNNAVFLVPGGR-------RYKVGAARFIG-PCKNRMIIQ------------

-----------------------------------IQGTIVAPDEPSEWD--PASPRLWL

LFSGLAGARIQGG---GLIDGSG--------SKWWANSCKIDRSKPCKGAPTPLK-----

--------------ILPLFHNALTIDSCRG-----------------------------V

SVRNLRLQNAQQMHLTVSRSRDVRLAS-VRVDSPEDSPN-TDGIHVADSTAVTIQSCRIA

TGDDCIS-------------------------------------ISNGSFAVRMRDIDCG

PG-HGISIG------SLGQGG--AFAAVDGVSLDGARVARAQNGVRIKTWQGGAGYVRNV

R---------FAGVRVDGVDHPIVIDQFYCDATRP------------------CRNRTS-

-----------------NVRVSGVVFRNITGTARRAEA----IRLACSDAVP-CVGIVLS

DIDLRREDGG-----------GEVQTVCNCAMGFDDGRVSPAAD----------------

>Osi000010.17

------ATGDHTFLHVPAAK-------SYLVWPVTLAG-PCREEIKLF------------

-----------------------------------ISGNIVAPESPDEWPEGGGGGGEWL

HFVGVSDLTLSGG---GVIDGRG--------HRWWARSCKAKHNATENCTTQ--------

-----------------AAPKALHFEDCQG-----------------------------I

SVMGITLQNSQESHLTFTRCSHVKANY-LRITSPEDSPD-TTGVHVVSSRNVHIMDDSIS

TGHDCVS-------------------------------------IVGNSTDVRLRAISCG

PG-HGISIG------GLGENR--SYHRVEKIKMDTLFISNTENGVRVKTFQGGCG-----

--------------------------------------------------------TAR-

-----------------KMKFGDILMKNVKNPIVIDQ------QNSSSNEIP-CGSKVQY

FSTYPLTLT------------NHNHPPFNLSTSFQPITIIPYSIPPTFLISRKNLKVAIF

>Osi000010.18

------ASTEPAVLAVPAGK-------TYQIWPVRLAG-PCKKKLKLM------------

-----------------------------------ISGTIAAPASPDEWA--GRDPTKWL

FVFRVDDLSVSGG---GTIDGMG--------AEWWARSCKRKK--TKPCSTV--------

-----------------SAPKALQFEECRR-----------------------------V

SVQGITMQNGPQFHLMFTRCTDVKASF-LRVVAPESSPN-TDGIHLNDTTHAQIMDNLIS

TGDDCVS-------------------------------------MVGNCSDVRVKDISCG

PG-HGISIG------SLGKNR--TTDRIENVRVDTCLLTNTTNGVRIKSWQSTSSSSLKL

G---------NFSSLWM---------------------------------------QTQ-

-----------------AVEVRKIEFAGIRGTSATEQA----IKLACSDAVP-CRDLELR

NVNLTMVGG------------GAASAFCHRASGKAAGAVVPASC----------------

>Osi044901.2

------ASTEPAVLAVPAGK-------TYQIWPVRLAG-PCKKKLKLM------------

-----------------------------------ISGTIAAPASPDEWA--GRDPTKWL

FVFRVDDLSVSGG---GTIDGMG--------AEWWARSCKRKK--TKPCSTV--------

-----------------SAPKALQFEECRR-----------------------------V

SVQGITMQNGPQFHLMFTRCTDVKASF-LRVVAPESSPN-TDGIHLNDTTHAQIMDNLIS

TGDDCVS-------------------------------------MVGNCSDVRVKDISCG

PG-HGISIG------SLGKNR--TTDRIENVRVDTCLLTNTTNGVRIKSWQ---------

---------------------------------------------------------TQ-

-----------------AVEVRKIEFAGIRGTSATEQA----IKLACSDAVP-CRDLELR

NVNLTMVGG------------GAASAFCHRASGKAAGAVVPASC----------------

>At1g80170

------SSKVKTRILVPENY-------TCLLRPIDLSG-PCKARLTLQ------------

-----------------------------------ISGTIIAPNDPDVWE--GLNRRKWL

YFHGLSRLTVEGG---GTVNGMG--------QEWWERSCKHNH--SNPCR----------

-----------------GAPTALTFHKCKN-----------------------------M

RVENLNVIDSQQMHIALTSCRRVTISG-LKVIAPATSPN-TDGIHISVSRGIVIDNTTVS

TGDDCIS-------------------------------------IVKNSTQISISNIICG

PG-HGISLG---------KSK--SWEEVRDITVDTAIISDTANGVRIKTWQGGSGLVSKI

I---------FRNIKMNNVSNPIIIDQYYCDSRKP------------------CANQTS-

-----------------AISIENISFVHVRGTSASKEA----IKISCSDSSP-CRNILLQ

DIDLEPSNGD-----------GFTESFCWEAYGSSSGQVYPPPC----------------

>At2g41850

------SSNGAVNLLVPKGN-------TYLLKSIQLTG-PCNSILTVQ------------

-----------------------------------IFGTLSASQKRSDYK--D--ISKWI

MFDGVNNLSVDGGD-TGVVDGNG--------ETWWQNSCKRNKAK---------------

---------------------ALTFYNSKS-----------------------------L

IVKNLKVRNAQQIQISIEKCSNVQVSN-VVVTAPADSPN-TDGIHITNTQNIRVSESIIG

TGDDCIS-------------------------------------IESGSQNVQINDITCG

PG-HGISIG------SLGDDN--SKAFVSGVTVDGAKLSGTDNGVRIKTYQGGSGTASNI

I---------FQNIQMDNVKNPIIIDQDYCDK-SK------------------CTTEKS-

-----------------AVQVKNVVYRDISGTSASENA----ITFNCSKNYP-CQGIVLD

RVNIKGG-----------------KATCTNANVVDKGAVLPQCN----------------

>At3g57510

------STNGVTTFLVPKGK-------TYLLKSTRFRG-PCKSLRNFQ------------

-----------------------------------ILGTLSASTKRSDYK--D--KNHWL

ILEDVNNLSIDGGS-TGIINGNG--------KTWWQNSCKIDKSKPCTKAPT--------

---------------------ALTLYNLKN-----------------------------L

NVKNLRVKNAQQIQISIEKCNKVEVSN-VEITAPGDSPN-TDGIHITNTQNIRVSNSDIG

TGDDCIS-------------------------------------IEDGTQNLQIFDLTCG

PG-HGISIG------SLGDDN--SKAYVSGINVDGAKFSESDNGVRIKTYQGGSGTAKNI

K---------FQNIRMENVKNPIIIDQDYCDK-DK------------------CEDQES-

-----------------AVQVKNVVYKNISGTSATDVA----ITLNCSEKYP-CQGIVLE

NVKIKGG-----------------TASCKNANVKNQGTVSPKCS----------------

>At3g07970

------SSTGIVYIVAPKNR-------DYMLKAVTFSG-PCKSSLIIF------------

-----------------------------------IYGRIEAWENPSDYK--E--RRHWI

VFENVNNLRVEGG---GRIDGNG--------HIWWPKSCKINPQLPCLGAPT--------

---------------------AVTFVECNN-----------------------------L

RVSNIRLENAQQMHLTFQDCKNVKALN-LMVTSPADSPN-TDGIHVSGTQNILIQDSIVR

TGDDCIS-------------------------------------IVSGSENVRATGITCG

PG-HGISIG------SLGEDN--SEAYVSNVVVNKATLIGTTNGVRIKTWQGGHGMAKNI

I---------FQDIIMKNVTNPIIINQDYCDRVEA------------------CPEQKS-

-----------------AVQVSNVLYKNIQGTSSRPIA----VKFVCSKNIP-CRGISMQ

NVKLVDQTQQ-----------DVSKASCSNVKLDTRGNVSPLCT----------------

>Osi000256.3

------SSLQPSIVLIPKGK-------RYLTKHITLSG-PCKSSITFM------------

-----------------------------------IEGTLVAPPKRSDWS--KETIRHWI

MFNGVSGLTVAGG---GTVDENG--------KIWWQNSCKTNAKLPCTEAPT--------

---------------------ALTFYSCSN-----------------------------L

KVENLKLLNSQQIHMSVEDCTDVRISS-LTITAPGTSPN-TDGIHITRSKNVQVAGCIIK

TGDDCMS-------------------------------------IEDGTENLHVKNMVCG

PG-HGISIG------SLGDHN--SEAHVNNVTVDPVRLYGTTNGARIKTWQGGWGYAKNI

V---------FQNMIMENVWNPIIIDQNYCDSATP------------------CKEQV--

---------------------SNVVFKNIRGTSASKEA----IKLDCSRNVP-CQGITLK

DV--TIKGGG-----------SDAKSTCGNAKWKKSG-----------------------

>Osi000256.9

------SSLQPSIVLVPKGK-------RYLTKHITLSG-PCKSSITFM------------

-----------------------------------IEGTLVAPPTRSDWS--KETIRHWI

MFNGVIGLTVDGG---GTVDGNG--------KIWWQNSCKTNAKLACTESPT--------

---------------------ALTFYSCSN-----------------------------L

KVENLKLLNSQQIHMSVEDCTNVRISG-LTITAPGTSPN-TDGIHITRSKNVQVTGCTIK

TGDDCMS-------------------------------------IEDGTENLHVKNMVCG

PG-HGISIG------SLGDHN--SEAHVNNVTIGTVRLYGTTNGARIKTWQGGRGYAKYI

V---------FQNMIMENVWNPVIIDQNYCDSATP------------------CKKQLSI

CVNNMEAYNLVFLSQTSAVQISNVVFKNIRGTSASKEA----IKLDCSRNVP-CQGITLN

DVKLTVKGGG-----------GDAKSTCRNAKWKKSGTVVPQPC----------------

>Osi000256.5

------SSAKPAVLLIPKGK-------KYLIKHTTLSG-PCKSSISLM------------

-----------------------------------VKGTLVASPERSDWS--KETIRHWI

LISGVTGLTVTGG---GTIDGNG--------KIWWQNSCKTNSKLPCTEAPT--------

---------------------ALTFYSCKN-----------------------------L

KVEYLKVVNSQQIQISVEDCTDVMVSR-LSITAPETAPN-TDGIHITRSRDVEVTDCMIK

TGDDCMS-------------------------------------IEDGTENLHVKNMVCG

PG-HGISIG------SLGDHN--SEAHVNNVTVDNVRLYGTTNGARIKTWQGGKGSAKNI

V---------FQNMVMDNVWNPIIIDQNYCDSSTP------------------CKQQKS-

-----------------AVEVSNVLFKNIRGTSASEEA----IMLHCSSSVP-CHGITLE

NVNLTVKGGI-----------DLKHEHPWT-LEDVE------------------------

>Osi000256.8

------------------------------------------------------------

------------------------------------------------------------

-------------------------------------TNKEN---------T--------

---------------------GKHFI---------------------------------L

KVENLKVVNSQQIQISVEDCTDVKMSR-LSITAPETAPN-TDGIHITRSRDVQVTDCTIK

TGDDCMS-------------------------------------IEDGTKNLHVKNMVCG

PG-HGISIG------SLGDHN--SEAHVNNVTVDNVRLYGTTNGARIKTWQGGKGSAKNI

V---------FQNMVMDNVWNPIIIDQNYCDSSTP------------------CKQQ---

----------------------IILIAEIC-SGGEQFA----VQEHQGHKCI-RGGH---

------RAAL-----------QQQCALPWHNLESISL-----------------------

>Osi002763.1

------ASAAPAVVLVPESR-------SYLLRQVTLSG-PCESTIKLM------------

-----------------------------------VKGTLVASPDMSNWN--ESNRRYWI

VVRGVDGLAVGGG---GTIDGNG--------EGWWENSCKINRALPCKGAPT--------

---------------------ALSFHTCDN-----------------------------L

SVNGLKMVNSQQIHMSVEDCTGVELAH-LSISAPGTSPN-TDGIHITHSKNVQVSDCTIK

T----------------------------------------------------VLNA---

------------------------------------------------------------

------------------------------------------------------------

------------------------------------------------------------

------------------------------------------------------------

>Osi001716.1

-----NSSDYPSVLLVPEGK-------TYLLMPVSFNG-PCRAT----------------

--------------------------------------TITAT--------------EWI

TFDNIDHLRVLGG---GTLNGNG--------HQWWINSCKTNRS-------M--------

---------------------ALYFRRCNH-----------------------------L

VVDGLQIRDSMQMHVVIAYSWRVLVSR-LLITAPGWSPN-TDGIHVSNSREVLMSGCIIS

TGDDCIS-------------------------------------IVTGSMFIRATGIFCG

PG-HGISIG------SLGANK--SWAHVSDVLVEKATLVGTTNGVRIKTWQGGDGHAERI

T---------FQDITMHNVTNPVIIDQNYCDSMTP------------------CHEQGS-

-----------------AVAINNIRYRNIRGTSSSKVA----INFVCSNSVH-CDGIVMQ

DVSLVGEG-------------SYVSCSYMNARVVELGYNFPY------------------

>At4g18180

-----KSSSSSVNLIIPR--------GEFSVG-SLRFSGPCTNV--SNL-----------

----------------------------------TVR--VKASTDLSKYRS----GGGWI

QFGWINGLTLTG---GGTFDGQG--------ALAWPFNNCTSDSNCKLLPTS--------

----------------------LKFVGMNR-----------------------------T

VVRRISSVNSKFFHIALVECRDFKGTR-LNITAPSDSPN-TDGIHIERSSNVYFSRSHIA

TGDDCVS-------------------------------------IGQGNSQITITSIKCG

PG-HGISVG------SLGRYP--NEKDVNGLVVKDCKISGTTNGIRIKTWANSPGLSAAT

N-------MTFENIIMNNVTNPIIIDQSYCPF-------------------SSCIS----

-------------NVPSKVELSEIYFKNIRGTSSSLVA----VQLHCSRGMP-CKKVYLE

NVHLDLSSSDGGRKQSSNRGNEAVSSSCRNVRANYIGTQIPPPC----------------

>Osi002260.2

------AATGAVTLVLPP--------GTYYIG-------PGT------------------

---------------------------------------LKAATDLKRF------GNDWV

EFGWVNHLIVSGQ-NGAAFDGQG--------AASWPFNKCPIRKDCKVLPTS--------

----------------------VLFVNNKN-----------------------------M

VVQNVASVNSKFFHMALLQCSGAKISG-VKISAPESSPN-TDGIHIERSNGVSIADTTIA

TGDDCIS-------------------------------------IGQGNDNIDVARVHCG

PG-HGMSVG------SLGRYV--GEGDVTRIHVRDMTFHGTMNGVRIKTWENSPTKSNAA

H-------MLFENLVMNDVQNPIIIDQKYCPY-------------------YNCEH----

-------------KFVSGVTIKDVQFKNIKGTATTQVA----VLLKC--GVP-CQGVVLQ

DVDLRY------------KGNGVSSSKCENVRAKYAGFQNPKPC----------------

>At1g43090

-----TRISGSSKIYVPK--------GTFYLG-GVEFVGPCKNP--IEF-----------

----------------------------------VIDGTLLAPANPRDIK-----QDTWI

NFRYINNLSISG---SGTLDGQG--------KYSWPLNDCHKNTNCPKLAMT--------

----------------------MGFAFVNN-----------------------------S

RIKDITSLNSKMGHFNFFSVHRFNITG-VTITAPGDSPN-TDGIKMGSCSNIHISNTNIG

TGDDCIA-------------------------------------ILSGTTNLDISNVKCG

PG-HGISVG------SLGKNK--DEKDVKHLTVRDTVFNGTSDGIRIKTWESSASKLVVS

N-------FIYENIQMIDVGKPINIDQKYCPH-------------------PPCEH----

-----------EKKGKSHVQIQDIKLKNIYGTSNNIVA----VNLQCSKSFP-CKNVELI

DINLKH----------TGLEKGHSTAMCENVDGSVRGKNGSSTLS---------------

>At1g43100

-----TRISGSSKIYVPK--------GKFYLG-GVEFVGPYKNP--IEF-----------

----------------------------------VIDGTLLAPANPRDIK-----QDTWI

NFRYINNLSISG---SGTLDGQG--------KYSWPLNDCHKNTNCPELAMT--------

----------------------MGFAFVNN-----------------------------S

RIKDITSLNSKMGHFNFFSVHRFNITG-VTITAPGDSPN-TDGIKMGSCSNIHISNTNIG

TGDDCIA-------------------------------------ILSGTTNLDISNVKCG

PG-HGISVG------SLGKNK--DEKDVKHLTVRDTVFNGTSDGIRIKTWESSASKLVVS

N-------FIYENIQMIDVGKPINIDQKYCPH-------------------PPCEH----

-----------EKKGKSHVQIQDIKLKNIYGTSNNIVA----VNLQCSKSFP-CKNVELI

DINLKH----------TGLEKGHSTAMCENVDGSVRGKNGSSTLS---------------

>At1g43080

-----TRISGSSKIYVPK--------GTFYLG-GVEFVGPCKNP--IEF-----------

----------------------------------VIDGTLLAPANPRDIK-----QDTWI

NFRYINNLSISG---SGTLDGQG--------KYSWPLNDCHKNTNCPKLAMT--------

----------------------MGFAFVNN-----------------------------S

RIKDITSLNSKMGHFNFFSVHRFNITG-VTITAPGDSPN-TDGIKMGSCSNIHISNTNIG

TGDDCIA-------------------------------------ILSGTTNLDISNIKCG

PG-HGISVG------SLGKNK--DEKDVKHLTVRDTVFNGTSDGIRIKTWESSASKIVVS

N-------FIYENIQMIDVGKPINIDQKYCPH-------------------PPCEH----

-----------EKKGESHVQIQDIKLKNIYGTSNNIVA----VNLQCSKSFP-CKNVELI

DINLKH----------TGLEKGHSTAMCENVDGSVRSKMVPQHC----------------

>At2g15450

-----KRISGSSKIYVPK--------GTFYLG-GVEFVGPCKNP--IEF-----------

----------------------------------IIDGTLLAPANPSDIK-----QDTWI

NFRYINNLSISG---SGTLDGQG--------KQSWPHNDCHTNPNCPKLAMT--------

----------------------MGFAFVNN-----------------------------S

NIKDITSLNSKMGHFNFFSVHHFNITG-VTITAPGDSPN-TDGIKMGSCSNIQISDTNIG

TGDDCIA-------------------------------------ILSGTTNLNISNVNCG

PG-HGISVG------SLGKNK--DEKDVKDLIVRDVIFNGTSDGIRIKNWESSASKILVS

N-------FVYENIQMIDVGKPINIDQKYCPH-------------------PPCEH----

-----------ERKGESHVQIQNLKLKNIYGTSKNKVA----VNLQCSKIFP-CKNVELI

DINIKQ----------NGVKDGSSTSVCENVDGFARGKMFPPHC----------------

>At2g15470

-----KRISGSSKIYVPK--------GTFYLG-GVEFVGLCKNP--IEF-----------

----------------------------------IIDGTLLAPANPSDIK-----QDTWI

NFRYINNLSISG---SGTLDGQG--------KQSWPHNDCHTNPNCPKLAMT--------

----------------------MGFAFVNN-----------------------------S

NIKDITSLNSKMGHFNFFSVHHFNITG-VTITAPGDSPN-TDGIKMGSCSNIQISDTNIG

TGDDCIA-------------------------------------ILSGTTNLNISNVNCG

PG-HGISVG------SLGKNK--DEKDAKDLIVRDVIFNGTSDGIRIKTWESSASKILVS

N-------FVYENIQMIDVGKPINIDQKYCPH-------------------PPCEH----

-----------ERK--SHVQIQNLKLKNIYGTSKNKVA----MNLQCSKIFP-CKNVELI

DINIKQ----------NGVKDGSSTSVCENVDGFARGKMFPQHC----------------

>At2g15460

-----KRISGSSKIYVPK--------GTFYLG-GVEFVGPCKNP--IEF-----------

----------------------------------IIDGTLLAPANPSDIK-----QDTWI

NFRYINNLSISG---SGTLDGQG--------KQSWPHNDCHTNPNCPKLAMT--------

----------------------MGFAFVNN-----------------------------S

NIKDITSLNSKMGHFNFFSVHHFNITG-VTITAPGDSPN-TDGIKMGSCSNIQISDTNIG

TGDDCIA-------------------------------------ILSGTTNLNISNVNCG

PG-HGISVG------SLGKSK--DEKDVKDLIVRDVIFNGTSDGIRIKTWESSASKILVS

N-------FVYENIQMIDVGKPINIDQKYCPH-------------------PPCEH----

-----------ERKGESHVQIQNLKLKNIYGTSKNKVA----VNLQCSKIFP-CKNVELI

DINIKQ----------NGVKDGSSTSVCENVDGFARGKMFPRIV----------------

>At2g26620

-----KRISGSSKIYVPK--------GTFYLG-GVEFVGPCKNP--IEF-----------

----------------------------------IIDGTLLAPANPNDIK-----QDTWI

NFRYINNLSISG---SGTLDGQG--------KQSWPLNDCHKNPNCPKLAIS--------

----------------------MGFAFVNN-----------------------------S

NIKDITSLNSKMGHFNFFFVHHFNITG-VTITAPSDSPN-TDGIKMGSCSNIQISNTNIG

TGDDCIA-------------------------------------ILSGTTKLNISNINCG

PG-HGISVG------SLGKNK--DEKDVKDLFVRDVIFNGTSDGIRIKTWESSASKILVS

N-------FVYENIQMIDVGKPINIDQKYCPH-------------------PPCEH----

-----------ERK--SHVQIQDLKLKNIYGTSKNKVA----VNLQCSKSFP-CKNVELI

DINIKQ----------NGLEDGSSITVCENVDGFARGKMFPQHC----------------

>At2g40310

-----KRKSGSSKIYVPK--------GIFYLG-GVEFVGPCKNP--IEF-----------

----------------------------------VIDGTLLAPANPSDIK-----QDTWI

NFRYINNLSISG---SGTLDGQG--------KQSWPLNDCHKNLNCPKLAMT--------

----------------------MGFAFVNN-----------------------------S

NIKDITSLNSKMGHFNFFSVHHFNITG-VTITASGDSPN-TDGIKMGSCSNMHISNTNIG

TGDDCIA-------------------------------------ILSGTTNLDISNVKCG

PG-HGISVG------SLGKNK--DEKDVKNLTVRDVIFNGTSDGIRIKTWESSASKILVS

N-------FVYENIQMIDVGKPINIDQKYCPH-------------------PPCEH----

-----------EQKGESHVQIQDLKLKNIYGTSKNKVA----MNLQCSKSFP-CKNIELI

DINIKS----------NGLENSSSIAVCENVDGSMSGKMVPQHC----------------

>At4g13760

--------SGSSKIYVPK--------GTFYLG-GVEFVGPCKNP--IEF-----------

----------------------------------LIDGTLLAPANPNNIK-----QDTWI

KFKYINDLSISG---SGTLDGQG--------KQSWPLNDCHKNPNCPKLAMT--------

----------------------MGFAFVNN-----------------------------S

NIKDITSLNSKMGHFNFFSVHHFNITG-VTITAPGNSPN-TDGIKMGSCSNIHISNTNIG

TGDDCIA-------------------------------------ILSGTTNLDISNVNCG

PG-HGISVG------SLGKNK--DEKDVKDLTIRDVIFNGTSDGIRIKTWESSASKILVS

N-------FLYENIQMIDVGKPINIDQKYCPH-------------------PPCEH----

-----------EQKGESHVQIQNLKLKNIYGTSKNKVA----VNLQCSKRFP-CKNIELI

DINITN----------NGLVDSFSTLVCENVDGSVSGKMVPQHC----------------

>At1g17150

------RWNGPSKMYIPL--------GTFYLG-GVTFVGPCDGK--ISF-----------

----------------------------------VIDGTLLAPPNNDDIK-----KEIWI

NFRYINYLTVFG---DGTLDGQG--------KKSWSLIDCQKDNNCPKLAIN--------

----------------------MGFDFVKN-----------------------------S

SMNGITSLNSKAGHFNFLSVDHFSITR-VNIIAPSNSPN-TDGIKIALSSNMQISNTHIS

TGDDCIA-------------------------------------MLSGNTNFDIYNVKCG

PG-HGISIG------SLGKNK--DEKNVNGLMVRNSVFTGTTNGIRIKTWESSASTIRII

N-------LVYENLQMINVENPIGIDQKYCPY-------------------PPCS-----

------------NMGDSHIQIRNVTLKNIWGTSKNKVA----VKFQCSKTFP-CKDVQLI

DINLTH----------HGV-DGPASALCENVDGSATGKMVPPHC----------------

>At1g78400

-----QWK-GLPRVYIPF--------GTFYLG-AVAFTGPCKSR--ISF-----------

----------------------------------IIKGTLLAPKDPNAIK-----QDSWI

IFRYVDYLTVSG---GGILDGQG--------SYSWPLNNCRQTHNCRALPMN--------

----------------------MGFQFVRF-----------------------------S

RLTRIKSINSKMGHLNFFSVQHFDITR-VNIKAPGDSPN-TDGIKIGSSNHMKIHHVDIA

TGDDCIA-------------------------------------ILSGTFNLDINKVNCG

PG-HGISVG------SLGKFK--GEKSVQGLIVRNSIFNGTSNGVRIKTWPSPGEPNLVS

N-------FLFKNLQMIDVQSPINIDQRYCPN-------------------PPCSFQVTS

L---------TRNKSFSKIQIRDVKFQNIWGTSTAKEA----VKLQCSKNVP-CKNVQLF

NINIVH----------RGR-DGPATSVCENVGGWIGGKISPPSC----------------

>At2g33160

-----QWSYGRSTVYIPS--------GIFYLR-QVTFSGPCKSS--ITF-----------

----------------------------------FIRGTLLAPRNPYAIN-----QEEWI

LFKYVDNLTVTG---GGLLDGQG--------SYSWPLNDCNKNTNCRTLAMN--------

----------------------IGFAFVKS-----------------------------S

KINGLRSINSKMGHFNLFSVEDFNITG-VTITAPGDSPN-TDGIKIGKSSHMQIYNVTIG

TGDDCIA-------------------------------------ILDGTSNLDISDVRCG

PG-HGISVG------SLGRYK--EEKNVQGLTVRNSIINGTTDGLRIKTWAKSVSQISVS

N-------FLYENIQMINVGNPIVIDQQYCPH-------------------GQCD-----

----------SPGKYASHVQIKDVKYNNIWGTSTSKEA----LKMQCSKTFP-CQDVELS

NINLHY----------VGR-DGLVTALCENVGGSIRGKIVPANC----------------

>Osi006459.1

------AGTGKQTIVFPK--------GDFVTG-PLNFTGPCNGD--IVI-----------

----------------------------------QLDGNLLGSTDLALFK------VNWM

EIKRVDNLEISG---KGKIDGQG--------AAVWSKNTWGKKYDCKILPNI--------

----------------------LVLDFVNNGLVSGISLVNPKFFHYEHVQIVVLDFVNNG

LVSGISLVNPKFFHMNMFKCKNITIKD-LTITAPEDSPN-TDDIHMGDSSKISIIDTVIG

TGDDCIS-------------------------------------IGPGTEGVNISSVTCG

PG-YGISVG------SLGRYK--DEKDVTDVTVKNCVLKKSTNGVRIKSYEDAASVLTAS

K-------FTYENIKMEDVANPIIIDTKYCPN-------------------KICTA----

------------NGNSK-VTIKDITFKNITGTSSTPEA----VSLFCSDKLP-CTGVTLN

DINVEY----------AGK-NNKTMAVCKNAKGTATGCLKELSC----------------

>Osi006459.3

------AGTGKQTIVVPK--------GDFLTG-PLNFTGPCKGD--IVI-----------

----------------------------------QLDGNLLGSTDLALFK------SNWI

EIMRLESLEISG---KGKLDGQG--------AAVWSKNSCAKKYDCKILPNT--------

----------------------LVLDFVNN-----------------------------G

LISGISLVNPKFFHMNVFKSKNITIKD-VTITAPGDSPN-TDGIHMGDSSKISIIDTVIG

TGDDCIS-------------------------------------IGPGTEGVNISGVTCG

PG-HGISVG------SLGRYK--DEKDVTDVTVKNCVLKKSTNGVRIKSYEDAASVLTAS

K-------FTYENIKMEDVANPIIIDMKYCPN-------------------KICTA----

------------NGNSK-VTIKDITFKNITGTSSTPEA----VSLLCSDKLP-CTGVTLN

DIKVEY----------SGT-NNKTMAVCKNAKGTATGCLKELSC----------------

>Osi000907.5

------AGAGKQTILIPK--------GDFMTG-AMELRGPCNGA--VTI-----------

----------------------------------QLDGNLLGSNDLSKYPGK--KMPNWV

EVRHVDNFVISG---KGKLDGQG--------PGVWSKNSCAKNYNCKLLPNT--------

----------------------LVLNTVND-----------------------------G

VVSGITLLNAKFFHMNIYRCKDIKISG-VTINAPGDSPN-TDGIHMGDSSKITIAATTIG

TGDDCIS-------------------------------------IGPGTDGVNITGVTCG

PG-HGISIG------SLGRYK--DERDVRDVSVTRCVLRKTTNGLRIKSYEDSVSPVTVS

K-------VSYDGVVMDHVDNPIIIDQKYCPN-------------------SICTS----

------------KGDSK-VSVRDVTFRNITGSSNTPAV----VQLLCSGKLP-CSGVAMQ

DVRVLY----------GGS-DKKTTRRLRPRAGEVHRVPQ--------------------

>Osi018831.1

------GLAGSQKVVIPK--------GEFMTG-PLNFSGPCKGY--VTV-----------

----------------------------------QIDGTMFGSNDIPKYN-----KGNWI

EILHIDNVLING---SGTLDGQG--------AAVWKD-------ECKILPNT--------

----------------------LVLDYVKN-----------------------------G

TVSGLKLVNAKFFHINVYMSKGVTIKN-VTITAVANSPN-TDGVHIGDSSEISVSDSTIA

TGDDCIS-------------------------------------VGPGSSRISIQGITCG

PG-QGISVG------CLGRFK--DEKDVTDVTVRDCVLRNTSNGVRIKSYEDVLSPITVS

R-------LTFENIRMDGVANPVIVDQKYWPE-------------------KDWPE----

------------KKGSKTVTIKNVTFRNITGTSNTPEA----VSLLCSDQLP-CSGMELL

DVNLKY----------DGK-DNKTMAVCTNAKGISKGSLQALAC----------------

>At3g07820

------QSSSPSKVVIPK--------GEFKLG-EIEMRGPCKAP--IEV-----------

----------------------------------TLQGTVKADG--NAIQ----GKEKWV

VFGNIDGFKLNGG---GAFDGEG--------NAAWRVNNCHKTFECKKLPIS--------

----------------------IRFDFILN-----------------------------S

EIRDISSIDAKNFHINVLGAKNMTMNN-IKIVAPEDSPN-TDGIHLGRSDGVKILNSFIS

TGDDCIS-------------------------------------VGDGMKNLHVEKVTCG

PG-HGISVG------SLGRYG--HEQDVSGIKVINCTLQETDNGLRIKTWPSAACSTTAS

D-------IHFEDIILKDVSNPILIDQEYCPW-------------------NQCNK----

-------------QKASTIKLVNISFKNIRGTSGNKDA----VKLLCSKGYP-CQNVEIG

DIDIKY----------NGA-DGPATFHCSNVSPKILGSQSPKAC----------------

>At3g07840

------QAPTASKVVITK--------GEFKLG-EIEMTGPCKAP--VEI-----------

----------------------------------NLQGTLKADG--KAIQ----GKERWV

VFLRINGFKLNGG---GIFDGEG--------NAAWRVNNCHKTFECKKLPIS--------

----------------------IRFDFVEN-----------------------------A

EIRDISSIDAKNFHINVLGAKNMTFDN-VKVIAPAESPN-TDGIHLGRSEGVKILNSKIA

TGDDCIS-------------------------------------VGDGMKNLHVENVMCG

PG-HGISVG------SLGRYV--HEQDVTGITVVNCTLQGTDNGLRIKTWPSAACATTAS

G-------IHFENIILNNVSNPILIDQEYCPW-------------------NQCNK----

-------------QKPSTIKLVDISFKNIRGTSGNKDA----VKLLCSKAHP-CANVEIG

NINLEY----------KGA-DGPPTFMCSNVSPKLVGTQNPKAC----------------

>At3g07830

------QSPTPRKVVIPK--------GQFKLG-EIMMSGPCKSP--VEI-----------

----------------------------------TLLGTVLADG--NSIH----GKEKWV

VFQRMDGFRLNGG---GTFDGEG--------NAAWRVNNCHKTFECKKLPIS--------

----------------------IRFDFVTN-----------------------------A

EIRDISSIDAKNFHINVIGAKNMTFDN-VKIMAPAESPN-TDGIHLGRSVGVSIINSRIS

TGDDCVS-------------------------------------VGDGMVNLLVKNVVCG

PG-HGISVG------SLGRYG--HEQDVSGIRVINCTLQETDNGLRIKTWPSAACSTTAS

N-------IHFENIILRNVSNPILIDQEYCPW-------------------NQCNK----

-------------QKSSSIKLANISFRRIRGTSGNKDA----VKLLCSKGYP-CENVQVG

DINIQY----------TGA-DGPATFMCSNVRPKLVGTQFPKAC----------------

>At5g48140

------QFPTKSTVMIPK--------GEYKLG-EIVMMGPCKAP--IRI-----------

----------------------------------ALLGTVKADG--NAN-----GKEKWV

AFRNINGFKLNGG---GVFDGEG--------NAAWRVNNCHKTFNCKKLPIS--------

----------------------IRFDFVTD-----------------------------A

KIRGITSLDAKHFHINVIGAKNVTFED-VKIIAPAESPN-TDGIHVGRSDGIKIINSFIS

TGDDCVS-------------------------------------VGDGMKNLLVERVTCG

PG-HGISIG------SLGRYS--HEENVSGIKIINCTLQETDNGLRIKTWPSAACTTTAS

D-------IHFENILLKNVSNPILIDQEYCPW-------------------NQCNK----

-------------QKPSTIKLANISFKKIRGTSGNKDA----VKLLCSKGYP-CQNVEVG

DVNIQY----------TGA-DGPATFQCSNVSPKLVGTQIPKAC----------------

>At3g07850

--------AAGSTITVPK--------GEYMVE-SLEFKGPCKGP--VTL-----------

----------------------------------ELNGNFKAPATVKTTK----PHAGWI

DFENIADFTLNGNK--AIFDGQG--------SLAWKANDCAKTGKCNSLPIN--------

----------------------IRFTGLTN-----------------------------S

KINSITSTNSKLFHMNILNCKNITLSD-IGIDAPPESLN-TDGIHIGRSNGVNLIGAKIK

TGDDCVS-------------------------------------IGDGTENLIVENVECG

PG-HGISIG------SLGRYP--NEQPVKGVTVRKCLIKNTDNGVRIKTWPGSP-PGIAS

N-------ILFEDITMDNVSLPVLIDQEYCPY-------------------GHCKA----

-------------GVPSQVKLSDVTIKGIKGTSATKVA----VKLMCSKGVP-CTNIALS

DINLVH----------NGK-EGPAVSACSNIKPILSGKLVPAAC----------------

>At3g14040

--------AAGSTITVPK--------GEYLVE-SLEFKGPCKGP--VTL-----------

----------------------------------ELNGNFKAPATVKTTK----PHAGWI

DFENLADFTLNGNK--AIFDGQG--------SLAWKANDCAKTGKCNSLPIN--------

----------------------IRFTGLTN-----------------------------S

KINSITSTNSKLFHMNILNCKNITLTD-IGIDAPPESLN-TDGIHIGRSNGVNLIGAKIK

TGDDCVS-------------------------------------IGDGTENLIVENVECG

PG-HGISIG------SLGRYP--NEQPVKGVTVRKCLIKNTDNGVRIKTWPGSP-PGIAS

N-------ILFEDITMDNVSLPVLIDQEYCPY-------------------GHCKA----

-------------GVPSKVKLSDVTIKNIKGTSATKVA----VKLMCSKGVP-CTNIALS

DINLVH----------NGK-EGPAVSACSNIKPILSGKLVPAAC----------------

>At1g02790

------DSPVPATLLVPK--------GTFLAG-PVIFAGPCKSK--VTV-----------

----------------------------------NVIGTIIATT---SGY----ATPEWF

LFERVDNLVLTGT---GTFHGKG--------EAVWKADGCGKKVQCNLPPTS--------

----------------------LKFRNMKN-----------------------------V

EINGISSVNAKAFHMFLVKTENVNIQN-IKLTAPAESPN-TDGIHLSNADNVSILDSTIA

TGDDCVS-------------------------------------VGRGSNNVTVERVICG

PG-HGLSVG------SLGKYK--NEEDVSGIHVNNCTMIETDNGLRIKTWGGSD-PSKAV

D-------IKFENIIMQSVKNPIIIDQNYG------------------------SR----

-------------GGDSQVAISDILFKNIRGTTITKDV----VQIMCSKSVP-CQGVNVV

DVNLDY----------VGK-TGGEKKSSS-------GGLVGALC----------------

>Osi000907.4

------AAPGRAAVVVPAAGGGGGGGGGYLLH-PVVFRGPCKGF--VEV-----------

----------------------------------RVAGVVRAPAGLDAFRG----YHEWI

NFAGIDGLLVTGG---GTFDGRG--------ASSWHLNDCPWKPDCVPPPSS--------

----------------------IKLGSVRN-----------------------------A

TITGVTSLDSKFFHVTIVGSHDVEVSH-VSIRAPRDSPN-TDGVHIQGSTGVRITDTAVA

TGDDCVS-------------------------------------VGPGSADVTVSGVSCG

PG-HGISVG------SLGRSP--GEADVRRLRVSNCTIAGTANGVRIKTWRGGQRSSAAA

AAAAAVSGLVFEDIVMRRVRNPIIIDQEYCPY-------------------LSCHHQSE-

-------------RRPSVVRISDVKFRNIRGVSATQVA----VKLSCSAASP-CRGVELR

DIDLRY-----------VRRGVATVSRCANVAGGVAGGTLVPPPC---------------

>Osi015814.1

------------------------------------------------------------

-------------------------------------------TILKVFT-----LHLFI

MLHGVHGHIYD-------------------------VTEYGAEPSNIDNKDT--------

----------------------LELDSVAN-----------------------------A

TVRGLRFLNSRGFHLNLHRSSHVAAER-LRIEAPAASRN-TDGIHVGLSSHVTVADSLVG

TGDDCVS-------------------------------------IGPGSSGVVVAGVACG

PG-HGISVG------SLGREE--GEGDVRGLVVRNCTVVGTTNGLRIKTWPGSPPSRAFN

--------ITFRDIVMSNVSNPIIIDQHYCPH-------------------AHCSDIA--

--------------KPSLVQISDVTYERIEGTSSSRVA----VQLLCSEDRP-CSGVRFD

RVNLSC-----------GRE------RCGSKFSNVEG---TKPT----------------

>At1g05650

------GSAASATVVVPT--------GTFLLK-VITFGG-PCKSK-ITF-----------

----------------------------------QVTGTVVAPEDYR-TFG---NSGSWI

LFNKVNRFSLVG----GTFDARG--------SGFWSCRKSG--QNCPPGVRS--------

----------------------ISFNSAKD-----------------------------V

IISGVKSMNSQVSHMTLNGCTNVAVRN-IRLVAPGDSPN-TDGFTVQFSTGVTLTGSTVQ

TGDDCVA-------------------------------------IGQGTRNFLISKLACG

PG-HGVSIG------SLAKQL--NEDGVENVTVSSSVFTGSQNGVRIKSWARPSTG--FV

RN------VFFQNLIMRNVQNPIIIDQNYCPS------------------NQGCPT----

-------------EHSG-VKITQVTYKNIQGTSATQEA----MKLVCSKSNP-CTGITLQ

DIKLTYNK------------GTPATSLCFNAVGKNLGVIQPTSC----------------

>At1g05660

------VSASSATVVVPK--------GTFLLK-VITFGG-PCKSK-ITF-----------

----------------------------------QVAGTVIAPEDYR-TFG---NSGFWI

LFNKVNRFSLVG----GTFDARA--------NGFWSCRKSG--QNCPPGVRS--------

----------------------ISFNSAKD-----------------------------V

IISGVKSMNSQVTHMTLNGCTNVVVRN-VKLVAPGNSPN-TDGFHVQHSTGVTFTGSTVQ

TGDDCVA-------------------------------------IGPGTRNLLITKLACG

PG-HGVSIG------SLAKEL--KEDGVENVTVSSSVFTGSQNGVRIKSWARPSNG--FV

RT------VFFQDLVMKNVENPIIIDQNYCPT------------------HEGCPN----

-------------EYSG-VKISQVTYKNIQGTSATQEA----MKLVCSKSSP-CTGITLQ

DIKLTYNK------------GTPATSFCFNAVGKSLGVIQPTSC----------------

>At2g43890

------RSAAAVTVTVPR--------GSFLLK-PVEFRG-PCRSR-ITF-----------

----------------------------------QIYGTIVAPSDYR-GLG---NSGYWI

LFVKVNRISIIG----GTLDARG--------ASFWACRKSG--KSCPVGARS--------

----------------------MTFNWAND-----------------------------V

VVSGLTSINSQTTHLVINSCNNVIVRK-VKLVAPDQSPN-TDGLHVQGSAGVTVTDGTFH

TGDDCIS-------------------------------------IGPGTRNLYMSKLNCG

PG-HGISIG------SLGRDA--NEAGVENITLINSVFSGSDNGVRIKTWARQSTG--FV

RN------VLFQNLIMKNVQNPIIVDQNYCPS------------------NQGCPK----

-------------QGSG-VKISQVVYRNIQGTSRTQQA----LTFDCSRSNP-CQAIRLH

DIKLTFN-------------GRSATSTCKNIKGVKAGVVMPQGC----------------

>At2g43880

------GSRARAMVYVPR--------GTYLVK-NLVFWG-PCKNI-ITF-----------

----------------------------------KNDGTLVAPANYW-DIG---NSGYWI

LFAKVNRISVYG----GTIDARG--------AGYWSCRKKG--SHCPQGARS--------

----------------------ISFSWCNN-----------------------------V

LLSGLSSFNSQNMHVTVHHSSNVRIEN-VRIRAPSGSPN-TDGIHVQSSSGVTISGGTIA

TGDDCIA-------------------------------------LSQGSRNIWIERVNCG

PG-HGISIG------SLGDYA--NEEGVQNVTVTSSVFTKTQNGVRIKTWARPSRG--FV

NN------VVFRNLIMNNVENPVIIDQNYCPN------------------GKGCPR----

-------------QSSG-VKISGVTFANIKGTSTTPIA----MKLDCSGSNH-CTGLRLQ

DIKLTYMR------------RSSAS-YCRNAHGRASGVMVPRNC----------------

>At2g43870

------ASSRPVTIVVPK--------GRFLLR-SVTFDGSKCKPKPVTF-----------

----------------------------------RIDGTLVAPADYR-VIG---NEDYWI

FFQHLDGITVYG----GVLDARG--------ASLWDCKKSG--KNCPSGATT--------

----------------------IGFQSSSN-----------------------------V

VVSGLTSLNSQMFHVVINGCNNVKLQG-VKVLAAGNSPN-TDGIHVQSSSSVSIFNTKIS

TGDDCVS-------------------------------------IGPGTNGLWIENVACG

PG-HGISIG------SLGKDS--VESGVQNVTVKTVTFTGTDNGVRIKSWARPSSG--FA

KN------IRFQHCVMNNVENPIIIDQNYCP-------------------DHDCPR----

-------------QVSG-IKISDVLFVDIHGTSATEVG----VKLDCSSKKP-CTGIRLE

DVKLTYQ-------------NKPAASACTHAGGIEAGFFQPNCL----------------

>At3g59850

------ASVKPVTILVPK--------GRFLLR-SIIFDGSKCKRKSVTF-----------

----------------------------------RIQGTLVAPSDYR-VIG---KENYWI

LFQHLDGISVYG----GVLDAQG--------ASLWSCKKSG--KNCPSGAT---------

--------------------------SSRN-----------------------------V

VISGLTSLNSQMFHVAINGCSNVKLDG-VKVSADGNSPN-TDGIHVQSSSTVSILNSKIS

TGDDCVS-------------------------------------IGPGTNGLWIENVACG

PG-HGISIG------SLGKES--VEVGVQNITVKTATFTGTENGVRIKSWARPSNG--FA

KN------IRFQHCVMNNVQNPIVIDQNYCPG------------------NENCPN----

-------------QVSG-IKISDVMFFDIHGTSATEVG----VKLDCSSKKP-CTGIRIQ

DVKLTYR-------------NKPATTDCSHAGGSEAGFQRPNSC----------------

>At2g43860

------ASANPTTIIVPK--------GRFLVG-NLVFHGNECKQAPISI-----------

----------------------------------RIAGSIVAPEDFR-IIA---SSKHWI

WFEDVTDVSIYG----GILDAQG--------TSLWKCKNNGG-HNCPTGAKS--------

----------------------LVFSGSNN-----------------------------I

KISGLTSINSQKFHIVIDNSNNVNIDG-VKVSADENSPN-TDGIHVESSHSVHITNSRIG

TGDDCIS-------------------------------------IGPGSTNVFIQTIRCG

PG-HGISIG------SLGRAE--EEQGVDNVTVSNVDFMGTNNGVRIKTWGKDSNS--FA

RN------IVFQHINMKMVKNPIIIDQHYCL-------------------HKPCPK----

-------------QESG-VKVSNVRYEDIHGTSNTEVA----VLLDCSKEKP-CTGIVMD

DVNLVSV-------------HRPAQASCDNANGSANDVVPFTPC----------------

>At1g65570

------GVEDSVVIYVPK--------GRYLVSGEVRFEGESCKSREITL-----------

----------------------------------RIDGTLIGPQDYS-LLG---KKENWF

SFSGVHNVTVLG----GSFDAKG--------STLWSCKANG--YNCPEGATT--------

----------------------LRFMDSNN-----------------------------V

KIKGVLSLNSQLFHIAINRCRNIKIED-VRIIAPDESPN-TDGIHIQLSTDIEVRNASIK

TGDDCIS-------------------------------------IGPGTKNLMVDGITCG

PG-HGISIG------SLAKSI--EEQGVENVTVKNAVFVRTDNGLRIKSWPRHSNG--FV

ER------VRFLGAIMVNVSYPILIDQNYCPG------------------DSSCPS----

-------------QESG-IKINDVIYSGIMGTSATEIA----IKMDCSEKVP-CTGIRMQ

AINLTSY-------------GEAAKTSCTNVSGKQLGLVTPSGC----------------

>Osi005342.2

------RSPEPATVYVPD--------GEFFVS-HSAFAGPC-SGGRMTV-----------

----------------------------------QIDGTLVAPFGYTGSAS---SGGEWI

VFDHVDGLTVSG----GTLDGRG--------ESLWACKAAG-HGGCPDGAT---------

---------------------SMKVLNSRD-----------------------------V

VISGVKSVNSELYHVVIDGCEGVAVQD-ARIVAPGSSPN-TDGIHVQSSSAVTITGASIQ

TGDDCIS-------------------------------------VGPGTSNLRVEHVSCG

PG-HGISIG------SLGKES--EEGGVENVTVSGAAFVGTENGLRIKTWGRAARSGAYV

RG------VVFEHALMRDVSNPIIIDQSYCPND----------------GGQGCPH----

-------------QSSD-VQISGVTYTDIQGSSASQVA----VKFDCSASKP-CSGLGLQ

DIKLTFDG------------GKPAEATCQHADGTASGVLMPPSC----------------

>Osi000068.9

------RSPRPATVYVPP--------GRYLLG-RATFVGPC-SSRAVAF-----------

----------------------------------SIAGTVVAPAGYAWDGA---TAGQWI

TFESVVGLTVSG----GTLDGRG--------DALWACKKQQPRGHCPTGAS---------

---------------------SLTISNARN-----------------------------V

VVEGVRSVSSELFHVVVLQSRGVTVRR-VTVEAPADSPN-TDGIHIHKSTNVAVYDAAIR

TGDDCVS-------------------------------------VGPGNSNLWIERVACG

PG-HGISIG------SLGKQQGMAVEAVQNVTVKTTWFTGTTNGLRIKTWGNSKRG--FV

RG------VTFSDSTMAGVGNPIIIDQHYCPDG----------------GCGGAAR----

-------------GSSSGIKISEVEYADVRGSSATPVA----VSFDCSRSNP-CSGIRLR

DVRLTYQGKSGRLQ------AAGAVSSCRNAQGTASGLVVPPSC----------------

>Osi011814.1

-----GDGGERPVMRVPA--------GTFLVG-RAYFRGPCRSAGGVVL-----------

----------------------------------AIDGTVVAPP-----AV---GNASWI

TFHYAHGLAIRG----GTLDGNG--------HAFWACKAAA-GRRCPPGTTVICFKYSKP

---------------------TLDISQSNN-----------------------------V

SVKRVTLVDSKNVHVSIFDCAGVTLQG-LRIAAPADSPN-TDGIHVALSRDVAVLGATVR

TGDDCVS-------------------------------------VGPGTSGVAIRNIRCG

PG-HGISIG------SLGGRAG--EGEVRNVTVESASLAGTQNGLRIKTWGKPFAG--RV

SG------VRFANVAMRDVQNPIVVDQNYCP------------------GNINCPG----

-------------QSSG-VKISDVEYEGITGTSATAVA----VRFDCSRSNP-CTGIRLR

NINLTYDGGGGGGG------KKPARSFCKNAGGSASGVVIPPSC----------------

>Osi013246.1

------RAHAAVAAHLLV--------SAFVAA-AAAAATYN-------------------

----------------------------------VIDYGAVGDDG---------------

------GVTDSA----RAF------------EAAWAAACAG--DAAAAAAT---------

------------------------------------------------------------

------------------ASAGVAIVD-TVVSAPGRSPN-TDGIHIKQSTGVTVRNAVIG

TGDDCVS-------------------------------------MVEGSSDVLIEAVTCG

PG-HGISIG------SLGDTP--EQVAVRNITVKGAALAGTTNGLRIKTWAKANAG--AV

TG------VSFSGVVMRNGSG---------------------------------------

------------------IEISGVSYTDVEGTSATATA----VRFDCSPSRP-CAGIAMR

DVRLRYQPPAAAAAA-----ERPAASFCRNAHGVAFGDVDPPSC----------------

>Osi006215.4

VSLQLSAPATAADDVAAGDDEEVTVVTTYRDIHPLTPPSPTTTTTPPTR-----------

----------------------------------LGSAAYSWDTASSHRSVS--SEEQFM

TMSREFTAMVAAG--TTMQTGPN--------DGNNGGDQLTSIGEDELEETN--------

---------------------PLAIVPDSHPIATP------------------------A

RSRASQLEVVPAAGPSPAPPVEARQVK-KEEVETKVSAW-QTAEVAKINNRFKREEVVIN

GWETEQG-------------------------------------MTDGTHGLHVTRLVCG

PG-HGISIG------SLGDDN--SRAEVSDIFIDTVHLYGTTNGARIKTWQGGS---GYA

KD------IVFQNMVMNSVKNPIIIDQNYCDS------------------AKKCET----

-------------QEGSAVEISNVVFKNIAGTTISKSA----ITLNCSKNYP-CYDISLQ

DINLEMVD-----------DNGATGSTCQNAKWRKSGTVVPQPC----------------

>At4g35670

-----GGDGDIKTLLIPS--------GKTFLLQPTVFQGPCKSSS-IKV-----------

----------------------------------QLDGTIVAPSDKFAWSD--PISRMWI

KFSTVSGLIIVGS---GTIDSRG--------SSFWEA-------------LH--------

------------------------ISKCDN-----------------------------L

RINGITSIDSPKNHISIKTCNTVAISN-INLFAPETSPN-TDGIDISDSTNINIFDSTIQ

TGDDCIA-------------------------------------INSGSSNINITGINCG

PG-HGISVG------SLGAGG--AEAKVSDVQVTHCTFNQTTNGARIKTWLGGQGYARNI

S---------FTDITLVNTKNPIIIDQHYI---------------------DKGRL----

-------------TEESAVAISNVKFVDFRGTSSNKNA----ITLKC-SETTHCVDVVMD

GIDITMAN------------GGKPKVNCQYVDGESS------------------------

>At5g27530

-----GGEENINTLFIPS--------GKTYLLQPIEFKGPCKSTS-IKL-----------

----------------------------------QLDGIIVAPSNITSWSN--PKSQTWI

SFSGVPGLMIDGS---GTINGRG--------SSFWE------------------------

------------------------------------------------------------

-----------------------------------NSPN-TDGIDISYSTNVNIFDSTIQ

TGDDCIA-------------------------------------INTGSSSINITQVNCG

PG-HGISVG------SLGADG--ENAAVSDVYVTQCTFNKTTNGARIKTWQGGQGYARNI

S---------FENITLINVQNPIIIDQQYT---------------------DKVLLDA--

-------------TKDSAVAISSVKYVGFQGTTLNEDA----IMLKC-SAITYCKDMVID

DIEVTMEN------------GEKPKVECENVEGESS------------------------

>At5g44830

-----GSGGNSKTFLIPS--------NQTFLLQPLTFQGPCKSPS-VQV-----------

----------------------------------KFDGKIVAPINKAAWSE--SKLFRWV

SFKEIIGLTVNGS---GTIHGRG--------SSFWKQ-------------LH--------

------------------------FQRCND-----------------------------L

KIIGITSFNSPRNHISISECKRVQLTK-IKLVAPEDSPN-TDGINISGSSDVDVYDTFIG

TGDDCVA-------------------------------------INNGSVNINITRMNCG

PG-HGISVG------SLGRDG--EESIVENVQVTNCTFFRTDNGVRIKTWPNGKGYARNI

L---------FKDLTFRESKNPIIIDQNYV---------------------DKGRLD---

-------------VEESAVAISNVTFTDIRGTSQRNEI----IKIDC-SEVTYCKDIVLD

KIDIATVD------------GNKPVVECSNVYGKSI------------------------

>At5g44840

-----GSGGNSKTFIIPS--------NKTFLLQPLTFQGPCKSPS-VQV-----------

----------------------------------KFDGKIVAPINKAAWSD--YKLFRWV

SFKEIIGLTVNGS---GTIHGRG--------SSFWKQ-------------LH--------

------------------------FQRCND-----------------------------L

MITGITSFNSPKNHISISECKRVKITK-IKLVAPHDSPN-TDGINISESSDVDIYDTVIG

TGDDCVA-------------------------------------INSGSMNINIARMNCG

PG-HGISVG------SVGRDG--EESIVENVQVTNCTFIRTDNGARIKTWPNGKGYAKNI

L---------FKSLTFRETKNPIIIDQNYV---------------------DKGRLD---

-------------VEESAVAISNVTFTDLRGTSKLDEI----IKIDC-SKVTYCKDIVLD

KIDIATVD------------GNKPIVECSNVYGKSI------------------------

>At3g15720

------SGTGDGQFVVPA--------GMTFMLQPLKFQGSCKSTP-VFV-----------

----------------------------------QMLGKLVAPS-KGNWKG--DKD-QWI

LFTDIEGLVIEGD---GEINGQG--------SSWWEHKGSRPTVSITLAALK--------

------------------------FRSCNN-----------------------------L

RLSGLTHLDSPMAHIHISECNYVTISS-LRINAPESSPN-TDGIDVGASSNVVIQDCIIA

TGDDCIA-------------------------------------INSGTSNIHISGIDCG

PG-HGISIG------SLGKDG--ETATVENVCVQNCNFRGTMNGARIKTWQGGSGYARMI

T---------FNGITLDNVENPIIIDQFYNGG-------------------DSDNAKD--

-------------RKSSAVEVSKVVFSNFIGTSKSEYG----VDFRC-SERVPCTEIFLR

DMKIETAS------------SGSGQCNQEQQHNRC-------------------------

>At5g17200

------KGGHNRKILVPQ--------GKTFMLKPLTFIGPCKSST-ISL-----------

----------------------------------SIRGNLVAPG--YTWYA--GRYTTWI

SFDSINGLVVTGG---GTIDGRG--------SLWWGN------VNNRPCAMH--------

------------------------FNNCNG-----------------------------L

RISNLRHLNSPRNHVGLSCSQNIEVRG-LRMTAPGDSPN-TDGIDISNCIGVHIHDSVIA

TGDDCIA-------------------------------------INSGSSHINITGIFCG

PG-HGISVG------SLGVTG--DFETVEEVRVKNCTFTKTQNGVRIKTYQNGSGYARKI

S---------FEDINMVASENPIIIDQTYHNGGTNGGISKSSSSYQNCHLTAKQRTPS--

-------------GNGKGVKVTDVRYARIRGSSASDQD----ITLNC-DADLGCSDIVMD

NVNMVSATF-----------GHKLFSSCKNAHGSLFAS----------------------

>At5g39910

-----NYIGSESIMEIPE--------GNTFLLQPIEFHGPCKSKK-IIL-----------

----------------------------------SISGNLIAPESPYEWKCNKDDCHQWI

EFAHINGLYIDGH---GLMACLK--------RPRGVV-----------------------

------------------------ISHSSN-----------------------------V

HISNIMVKDSPNFQMSLEDSKWVIVKQ-LTITADGDSPN-TDGIHIQRSQNVIVYDSNIR

TGDDCIS-------------------------------------IGDGSKYINISRISCG

PG-HGISIG------SLGRYG--TKETVENVVVRDCTFRETTNGVRIKTWQTKAVEIKNV

M---------FNHIHGTSIKKPFVQLLCSK------------------------------

-----------------SVPCRDIFMNDINIHDENEE-----------EEKKYHKSLSRH

DDHPSAECI-----------NVKGESNGVMKPKLA-------------------------

>At1g80140

-------------VNVPA--------GKVFLLNSLHFTGPCIPKP-LLF-----------

----------------------------------IIDGEMIAQSDPQKWGNGENGVIPWL

IFDQVDGLAIVGR---GLLDGQG--------KSWWDIHCR----DHPGPMMT--------

------------------------FSNCGN-----------------------------V

TLKSLRFRNSAQTHVLVMGSQNVYIDD-IKITSPEASPN-TDGIHITSSTAVSINHSDIA

TGDDCVS-------------------------------------IGDQVNNLNVTFMNCG

PG-HGVSIG------SLGRGG--TEVTVENIRVSHVNFTGTTNGARIKTWPGGTGYVRGI

E---------FFDIRFSSVQNPIIIDQFYG----------------------CAPTCV--

-------------ETMKGVHIEKVRYMKMSGTSATKVA----MKLECSGESVPCSNLFMR

DIDLSPADG-----------IDSVSSLCSFAHGSAQGIIRPLSC----------------

>At4g32370

------------------------------------FLFD--------------------

----------------------------------KIDGKLEGPRKPIYWK--NKENRSWL

GFKDVEGLVINGS---GVLNPHG--------EAWWKSVSLSKRPTGGRGLAKNILYENIT

LIDAGYPIIINQHYFDNKKKHYFDKSFLKESGVKVDNI-----------------TFRYF

EGTSSSEIPIKLDCDETENCHNITMEH-INITSPTPGKN-LTAYCKFADGLELLFSVILI

ASYHLQHGDAHVNLSIKD--------------------------FLSDAKNTTAAHSQAF

KEAWNALCEA-----NVNGTTTSLVINANETYIVQPQLFQGPCASRNIHIQGGLGVAKNI

L---------YENITLTDTKYPIIIDQHYCN------------------GGHNCTK----

-------------EAMTAVKVSNVTFRYFTGTCANDIA----IKLDCDEVTG-CKDIVME

HINITSSSTK-----------RPLTAYCQFADIISHFV----------------------

>At4g32380

--------------------------NGRMILTMKDFISNSNSTNVDHS-----------

----------------------------------QIDGTIEAPKMANDWG--RNKLDCWL

CFEKVTGLVLTGS---GVLNTHG--------ESWWSSVALQSRPVYSIGSLG--------

------------------------------------------------------------

EGGAS------------EVVQNVNVRH-CTFTGTQNG-----------------------

------------------------------------------------------------

--------------------------------------------ARIKTWPGGQGFVKNI

L---------YEDITLINANFPIIIDQQYRDN-----------------AGQYKQS----

-------------AGATAVKVSDVTFRSFTGTCAAPIA----IKLDCDPNTG-CDNIVME

QINIASSSPK-----------TPLTSYCKFAHVVSRFVS---------------------

>At1g19170

--------SGGGQLNVP---PGRWLTAPFNLTSHMTLFLAEDSEILGVE-----------

-----------------------------DEKYWPLMPPLPSYGYGRERPGP--RYGSLI

HGQNLKDIVITG--HNGTINGQG--------QSWWKKHQRRLLN-YTRGPLV--------

-----------------------QIMWSSD-----------------------------I

VIANITMRDSPFWTLHPYDCKNVTIRN-VTILAPVTGAPNTDGIDPDSCEDMVIEDCYIS

TGDDAIA-------------------------------------IKSGWDQFGIAYGRPS

TNILIRNLVVRSVISAGVSIGSEMSGGISNVTIENLLIWNSRRGIRIKTAPGRGGYIRNI

T---------YKNLTLDNVRVGIVIKTDYNEHADDN------------------------

------------YDRKAYPILSGFSFAGIHGQGVRVPV------RIHGSEQIPVRNVTFR

DMSVGLTYKK------------KHIFQCSFVKGRVFGSIFPRPC----------------

>At3g42950

---------GGGQLNVP---PGRWLTAPFNLTSYMTLFLSENAEILALQ-----------

-----------------------------DEKYWSLLPPLPSYGYGREHHGP--RYGSFI

HGQNLRDVVVTG--NNGSINGQG--------QTWWKKYRQKLLN-HTRGPLV--------

-----------------------QIMWSSD-----------------------------I

VFANITLRDSPFWTLHPYDCKNVTITN-MTILAPVFEAPNTDGIDPDSCEDMLIENSYIS

VGDDGIA-------------------------------------IKSGWDQYGTTYGKPS

KNILIRNLIIRSMVSAGISIGSEMSGGVSNITVENILIWSSRRGVRIKTAPGRGGYVRDI

T---------FRNVTLDELRVGIVIKTDYNEHPDGG------------------------

------------FNPQAFPILENINYTGIYGQGVRVPV------RIQGSKEIPVKNVTFR

DMSVGITYKK------------KHIFQCAYVEGRVIGTIFPAPC----------------

>Osi007221.3

-------ERGGGRLVVP---AGRWLTAPFNLTNRMTLFLAAGAEILGVQ-----------

-----------------------------DERYWPLMSPLPSYGYGREHRGP--RYGSLI

HGQDLKDVTITGG-QNGTINGQG--------QSWWSKFRKKVLN-HTRGPLV--------

-----------------------QLMRSSN-----------------------------I

TISNITLRDSPFWTLHIYDCKDVTISD-TTILAPIVGAPNTDGIDPDSCENVVIKNCYIS

VGDDGIA-------------------------------------IKSGWDQYGIAYGRPS

TNIIIHNVTIRSMVSAGVSIGSEMSGGVSNVLVENVHIWDSRRGVRIKTAPGRGAYVSNI

T---------YRNITLEHIRVGIVIKTDYNEHPDEG------------------------

------------FDPKAVPIIENISYSSIHGHGVRVPV------RIQGSAEIPVKNVTFH

DMSVGLVDRR------------NHVFQCSFVQGQVIGYVFPVPC----------------

>Osi004792.3

---------------IP---S-------FREVQKLVLLSGFD------R-----------

-----------------------------DERYWPLMPALPSYGYGRERKGP--RFGSLI

HGQNLKDVVITG--HNGSINGQG--------EVWWMKHRRRILN-NTRPPLL--------

-----------------------QLMWSKD-----------------------------I

IVANITLKNSPFWHFHPYDCTNITVSN-VTILAPISSAPNTDGIDPDSCQDVLIENCYIS

VGDDAIA-------------------------------------VKSGWDQYGIAYGRPS

RNIVIRNVMARSLVSAGISIGSEMSGGIANVTVEDVRIWESRRGLRIKTAIGRGGYIHDI

S---------YRNITFDNVRAGIVIKVDYNEHADDG------------------------

------------YDRDAFPDITNISFKEIHGRGVRVPV------RAHGSSDIPIKDISFQ

DMSIGISYKK------------KHIFQCSFIEGRVIGSVFPKPC----------------

>At2g23900

--------DGGVQLIVP---PGKWLTGSFNLTSHFTLFIQKGATILASQ-----------

-----------------------------DESEYPVVAPLPSYGQGRDAAGP--TFASLI

SGTNLTDVVITG--NNGTINGQG--------KYWWVKYRSGGFKGITRPYTI--------

-----------------------EIIFSQN-----------------------------V

QISNITIIDSPAWNIHPVYCNNVIVKG-VTILAPIDSPN-TDGINPDSCTNTLIEDCYVV

SGDDCIA-------------------------------------VKSGWDQFGIKVGMPT

QQLSIRRLTCISPDSAGIALGSEMSGGIKDVRIEDITLLQTQSAIRIKTAVGRGGYVKDI

F---------ARRFTMKTMKYVFWMSGAYNQHPASG------------------------

------------FDPKAMPVITNINYRDMTADNVTQPA------RLDGFKNDPFTKICMS

NIKIDLAAEPK-----------KLLWNCTSISGVSSK-VTPKPC----------------

>At3g48950

---------GGAQLVVP---PGKWLTGSFNLTSHFTLFIQRGATILASQ-----------

-----------------------------DESEWPVIAPLPSYGKGRDGTGTG-RFNSLI

SGTNLTDVVITG--NNGTINGQG--------QYWWDKFKKKQFK-ITRPYLI--------

-----------------------EILFSKN-----------------------------I

QISNITLIDSPSWNIHPVYCNSVIVKS-VTVLAPVTVPN-TDGINPDSCTNTLIEDCYIV

SGDDCIA-------------------------------------VKSGWDQYGIKFGMPT

QQLSIRRLTCISPKSAGVALGSEMSGGIKDVRIEDVTLTNTESAIRIKTAVGRGAYVKDI

Y---------ARRITMKTMKYVFWMSGNYGSHPDEG------------------------

------------FDPKALPEITNINYRDMTAENVTMSA------SLDGIDKDPFTGICIS

NVTIALAAKAK-----------KMQWNCTDVAGVTSR-VTPEPC----------------

>At3g61490

--------EGGAQLFVP---AGKWLTGSFNLTSHFTLFLHKDAILLAAQ-----------

-----------------------------DLNEYPILKALPSYGRGRDAAGG--RFASLI

FGTNLSDVIITG--NNGTIDGQG--------SFWWQKFHGGKLK-YTRPYLI--------

-----------------------ELMFSDT-----------------------------I

QISNLTFLDSPSWNIHPVYSSNIIVKG-VTIIAPVKSPN-TDGINPDSCTNTRIEDCYII

SGDDCIA-------------------------------------VKSGWDEYGISFGMPT

KHLVIRRLTCISPYSAAIALGSEMSGGIEDVRAEDITAYQTESGVRIKTAVGRGAFVKNI

Y---------VKGMNLHTMKWVFWMTGNYKAHADSH------------------------

------------YDPHALPEITGINYRDIVAENVSMAG------RLEGISGDPFTGICIS

NATISMAAKHK-----------KAIWMCSDVEGVTSG-VDPKPC----------------

>At4g23500

-------DYGGSLLFVP---AGRWLTGNFNLTSHFTLFLHRDAVILASQ-----------

-----------------------------EESDYEVIEPLPSYGRGRDTDGG--RFISLL

FGSNLTDVVITG--ENGTIDGQG--------EPWWGKFKRGELK-YTRPYLI--------

-----------------------EIMHSDG-----------------------------I

QISNLTFLNSPSWHIHPVYSSNIYIQG-LTILAPVTVPN-TDGINPDSCTNTRIEDCYIV

SGDDCIA-------------------------------------VKSGWDQYGINYGMPT

KQLLIRRLTCISPDSAVIALGSEMSGGIEDVRAEDIVAINSESGIRIKTAIGRGGYVKDV

Y---------VRGMTMMTMKYVFWMTGSYGSHPDDH------------------------

------------YDPKALPVIQNINYQDMVAENVTMPA------QLAGISGDQFTGICIS

NVTITLSKKPK-----------KVLWNCTDVSGYTSG-VTPQPC----------------

>Osi003614.4

--------GGGAMLYVP---AGKWLTGPFNLTSHFTLFLHSDAVILGSQ-----------

-----------------------------DMGEWPIIDPLPSYGRGRDKAGG--RYASLI

GGSNLTDVVITG--ANGTIDGQG--------AMWWSKFHSNKLK-YTRGYLI--------

-----------------------EVMHSDT-----------------------------V

VISNVTLVNSPAWNIHPVYSSNIVVQG-VTILAPTHSPN-TDGINPDSCSHVRIEDCYIV

SGDDCVA-------------------------------------IKSGWDEYGIAYGMPS

QHIVVRRLTCVSPTSAVIALGSEMSGGISDVRAEDITAVNSESPVRIKTPVGRGAYVRDV

F---------VRGMSLDTMKWVFWMTGNYKSHPDDG------------------------

------------YRPQRHPRRG--------QHQLPGRG------GHGGVQG---------

--------------------------------GRQAG-GHPG------------------

>Osi012001.3

-------RIKAALKYVPR--AVCWELEPGDVSLQLRWLPIN-------------------

------------------------------LEDWPLIAPLPSYGRGRDEPGP--RYSNFI

AGSNLTDVIITG--RNGTINGQG--------QVWWDKFHAKELT-YTRGYLL--------

-----------------------ELLYSNN-----------------------------I

IISNVTFVDSPSWNLHPTYCTNVTISG-ITILAPLNSPN-TDGIDPDSSSHVKIEDSYIV

SGDDCIA-------------------------------------VKSGWDQYGIKFNMPS

QHILIRRLTCISPTSAMIALGSEMSGGIRDVRAVDNVAIDTESAVRIKSGVGRGGYVKDV

F---------VRGLSLHTMKWVFWMTGNYGQHPDNS------------------------

------------SDPNALPEVTGINYSDVFAENVTMAG------RMEGIPNDPYTGICMS

NVTAQLAPDAK-----------KLQWNCTDVKGVASD-VSPVPC----------------

>At3g06770

---------GGAQLYVP---PGHWLTGSFSLTSHLTLFLENGAVIVAS------------

----------------------------QDPSHWEVVDPLPSYGRGIDLPGK--RYKSLI

NGNKLHDVVVTG--DNGTIDGQG--------LVWWDRFTSHSLK-YNRPHLI--------

-----------------------EFLSSEN-----------------------------V

IVSNLTFLNAPAYSIYSIYSSHVYIHK-ILAHSSPKSPY-TIGIVPDSSDYVCIQNSTIN

VGYDAIS-------------------------------------LKSGWDEYGIAYSRPT

ENVHIRNVYLRGASGSSISFGSEMSGGISDVVVDNAHIHYSLTGIAFRTTKGRGGYIKEI

D---------ISNIDMLRIGTAIVANGSFGSHPDDK------------------------

------------YDVNALPLVSHIRLSNISGENIGIAG------KLFGIKESPFSSVTLS

NVSLSMS-SGS-----------SVSWQCSYVYGSSES-VIPEPC----------------

>At3g16850

---------GGAQLYVP---PGKWLTGSFNLTSHLTLFLEKGATILAS------------

----------------------------PDPSHWDVVSPLPSYGRGIELPGK--RYRSLI

NGDNLIDVVITG--ENGTFDGQG--------AAWWEWLESGSLN-YSRPHII--------

-----------------------EFVSSKH-----------------------------I

LISNLTFLNAPAINIHPVYCSQIHIRK-VLIETSVDSPH-VLGVAPDSSDNVCIEDSTIN

VGHDAVS-------------------------------------LKSGWDQYGIHYGRPT

TAVHIRNLRLKSPTGAGISFGSEMSGGVSDVTVERLNIHSSLIGVAFRTTRGRGGYIRNI

T---------ISDVDLTSVDTAIVANGHTGSHPDDK------------------------

------------FDRDALPVVTHIVMRNFTGVDIGVAG------NLTGIGESPFTSICLA

DIHLQTR-SEE-------------SWICSNVSGFSDD-VSPEPC----------------

>Osi003986.1

---------GGAQLYVP---KGRWLTGSFNLTSHLTLFLEEEAVIIGTKLLWMDRRKNLV

KISRSVGFKLGLAFSCFVPKFMQTWSIWKDPSQWPIVEPLPSYGQGLDLPGP--RHRSLI

NGYNLSDVVITG--NNGVIDGQG--------SV---------------------------

------------------------------------------------------------

--------------------CNVKVHN-VTIKTSLDAPL-TDGIVPDSCSNVCIEDSSIS

VGHDAIS-------------------------------------LKSGWDNYGISFGRPT

SDIHISRVDLQASSGAALAIGSEMSGGISDIHVDHIRIGSSSKGISFRTTPGRGGYIAEV

V---------VADVVMDSVHLAIEFTGNWSSHPDDH------------------------

------------FDPSFLPVIDQITLKNMEGTNISVAG------VLSGIEGDPFSAICLS

NLNFSIADSAP-----------SSAWTCSNVHGYSEL-VFPKPC----------------

>Osi006048.1

---------GGAQLYVP---RGRWLTGSFNLTSHLTIFLEKDAVIIG-------------

---------------------------AKEVSEWPIVEPLPSYGQGIDLPGA--RHRSLI

NGHNVTDVVIT-------------------------------------------------

------------------------------------------------------------

----------------------------------------------DSCSNMCIEDSSIS

VAHDAIS-------------------------------------LKSGWDNYGITIGRPA

SDIHISRVDLQASLGAALAFGSEMSGGISDIHVDHLNIHGSSRGILFKTAPGRGGYIRDV

V---------ISDVQMEDVNVAIKFTGDWSTHPDNH------------------------

------------FDPSALPMINRITLKNMVGTNISVAG------VLSGINGDPFTNICLS

NISFSLADSTQ-----------SSSWSCSNISGYSEL-VFPEPC----------------

>Osi000386.5

---------GGAQLFVP---AGRWLTGSFSLISHLTLSLDKDAEIIGSP-----------

-----------------------------DSSDWPVIDPLPSYGRG--------------

--------------ANGTIDGQG--------AIWWDWFHSNTLN-YTRPHLV--------

-----------------------ELMYSTD-----------------------------V

VISNLTFKNSPFWNIHPVYCSQVLVQH-VTILAPLNSPN-TDGIDPDSSTNVCIDHCYVR

NGDDVIV-------------------------------------IKSGWDEYGISFARPS

TNISISNITGETRGGAGIAFGSEMSGGISEVRAEGLRIVNSMHGIRIKTAPGRGGYVKNV

Y---------ISDVSMDNVSMAIRITGNFGEHPDDK------------------------

------------YDRNALPMISNITIENVVGVNVGVAG------ILEGIEGDNFSSICLS

NVSLSVQS--------------MHPWNCSLIEGYSNS-VIPESC----------------

>Osi003045.1

---------GGAELFVP---AGRWLTGSFNLISHLTVSLDADAVIIGSQ-----------

-----------------------------DSSDWPVIDPLPSYGRGRELPG---------

--------------ANGTIDGQG--------ELWWNWFHNHTLN-YTRPPLL--------

-----------------------ELMYSDR-----------------------------V

VISNLTFMNAPFWNIHPVYCSQVLVQH-LTILAPISSPN-TDGIDPDSSSNVCIEDCYIR

NGDDIVV-------------------------------------IKSGWDEYGISFAHPS

SNISIRNITGQTRNSAGIAFGSEMSGGISDVRAEGLRFINSVHGIRIKTAPGRGGYVKNI

Y---------IADVSMDNVSIAIRITGNYGEHPDDN------------------------

------------YDKNVLPVISNITIKNVVGVNIGTAG------MLLGIQGDIFSNICLS

NVSLSSKS--------------ADPWNCSLVKGFSNS-VAPEIC----------------

>At3g62110

-------DKGGAKLFVP---AGQWLTGSFDLISHLTLWLDKGATILGS------------

-----------------------------TAKNWPVVDPLPSYGRGRELPGR--RHRSLI

YGQNLTDVVITG--ENGTIDGQG--------TVWWDWFRNGELN-YTRPHLV--------

-----------------------ELMNSTG-----------------------------L

IISNLTFLNSPFWNIHPVYCRDVVVKN-LTILAPLESPN-TDGVDPDSSTNVCIEDCYIV

TGDDLVS-------------------------------------IKSGWDEYGISYARPS

SKIKINRLTGQTTSSSGIAIGSEMSGGVSEIYIKDLHLFNSNTGIRIKTSAGRGGYVRNV

H---------ILNVKLDNVKKAIRFTGKYGEHPDEK------------------------

------------YDPKALPAIEKITFENVNGDGIGVAG------LLEGIEGDVFKNICFL

NVTLRVKKNSK-----------KSPWECSNVRGYSQ------------------------

>At4g23820

-------RRGGTLLYIP---PGVYLTESFNLTSHMTLYLAKGAVIRAVQ-----------

-----------------------------DTWNWPLIDPLPSYGRGRELPGG--RYMSFI

HGDGLRDVVITG--QNGTIDGQG--------EVWWNMWRSRTLK-YTRPNLI--------

-----------------------EFKDSKE-----------------------------I

IISNVIFQNSPFWNIHPVYCSNVVIHH-VTILAPQDSPN-TDGIDPDSSYNVCIEDSYIS

TGDDLVA-------------------------------------IKSGWDQYGIAYGRPS

SNITIRRITGSSP-FAGIAIGSETSGGIKNIIAEHITLSNMGVGVNIKTNIGRGGYIKNI

K---------ISDVYVDTAKYGIKIAGDTGDHPDEN------------------------

------------YNPNALPVVKGIHIKNVWGVNVRNAG------SIQGLKGSPFTGICLS

EINLHGSLNS------------YKTWKCSDVSGTSLK-VSPWPC----------------

>At5g41870

--------NEGTLLYVP---RGVYLTQSFNLTSHMTLYLADGAVIKAVQ-----------

-----------------------------DTEKWPLTDPLPSYGRGREHPGR--RYISFI

HGDGLNDVVITG--RNGTIDGQG--------EPWWNMWRHGTLK-FTRPGLI--------

-----------------------EFNNSTN-----------------------------I

LVSHVVLQNSPFWTLHPVYCSNVVVHH-VTILAPTDSYN-TDGIDPDSSSNVCIEDSYIS

TGDDLVA-------------------------------------VKSGWDEYGIAYNRPS

RDITIRRITGSSP-FAGIAIGSETSGGIQNVTVENITLYNSGIGIHIKTNIGRGGSIQGI

T---------ISGVYLEKVRTGIKISGDTGDHPDDK------------------------

------------FNTSALPIVRGITIKNVWGIKVERAG------MVQGLKDSPFTNLCFS

NVTLTGTKRS-------------PIWKCSDVVGAADK-VNPTPC----------------

>Osi001110.5

--------RGGALLYVP---AGVWLTGPFNLTSHMTLFLARGAVIRATQ-----------

-----------------------------DTSSWPLIDPLPSYGRGRELPGG--RYMSLI

HGDGLQDVFITG--ENGTIDGQG--------SVWWDMWRKRTLP-FTRPHLL--------

-----------------------ELISSTD-----------------------------V

IISNVVFQDSPFWNIHPVYCSNVVITN-VTVLAPHDSPN-TDGIDPDSSSNVCIEDSYIS

TGDDLIS-------------------------------------IKSGWDEYGIAFGRPS

SGITIRRITGSGP-FAGFAVGSETSGGVENVHVEHLNFFGMGVGIHVKTNSGRGGFIRNI

T---------VSEVTLNGARYGLRIAGDVGGHPDAS------------------------

------------YDPSKLPVVDGVTIKNVWGQNIRQAG------LVRGIRDSVFSRICLS

NVKLYG-GDS------------VGPWKCRAVSGGALD-VQPSPCA---------------

>Osi006881.1

--------PGGALLYVP---PGVWLTGPFNLTSHMTLFLARGAVIRATQ-----------

-----------------------------DTSSWPLIEPLLSYGRGRELPGG--RYMSLI

HGNGLQDVVITG--DNGTIDGQG--------SAWWDMWKKGTLP-FTRPHLL--------

-----------------------ELMNSSD-----------------------------V

VVSNVVFQDSPFWNIHPVYCR-------FQQQRLHRG-------------------RYIS

TGDDLIA-------------------------------------IKSGWDEYGMAYGRPS

SHITIRRITGSSP-FAGFAVGSETSGGVEHVLAEHLNFFSSGFGIHIKTNTGRGGFIRNV

T---------VSRRDAGLRPLRPEDRRRCRRAPRRP------------------------

------------LRPERAPRRGRPDDQERPGPEHQGGR------VDQGDRD---------

----------------------VGLLQDLPVQREAQ------------------------

>Osi004771.1

----------GTTLLVP---AGTWLTGSFNLTSHMTLFLARGAVLKATQ-----------

-----------------------------ETRSWPLAEPLPSYGRGRELPGA--RYASFI

HGDGLHDVVITG--DKGIIDGQG--------DVWWNMWRQRTLQ-HTRPNLL--------

-----------------------EFMHSSG-----------------------------I

HISNIVLKNSPFWNIHPVYCDNVVITN-MMIIAPHDSPN-TDGVDPDSSTNVCIEDSYIS

TGDDLVA-------------------------------------IKSGWDEYGIAYGRPS

SGITIRRVRGSSP-FSGIAIGSEASGGVSDVLVEDCSIFNSGYGIHIKTNIGRGGFIRNI

T---------VDNVRMNSVRNGLRIAGDVGDHPDEH------------------------

------------FSQLALPTVDGVSIKNVWGVNVQQPG------SIEGIRNSPFTRICLA

NVKLFG-WRN------------NAAWKCRDVHGAALG-VQPGPCA---------------

>At4g33440

--------EGGAQLNVP---EGTWLSGSFNLTSNFTLFLERGALILGSK-----------

-----------------------------DLDEWPIIEPLPSYGRGRERPGG--RHISLI

HGDNLTNVVITV--S--------------------VLRFSVMIK-EKMG-----------

-----------------------QLMDRGK-----------------------------C

GGSYGGTEHWCIREAILLNSRTLIISSSLTSLCSILLSGQSILFIADSSTNVCIEDCYIE

SGDDLVA-------------------------------------VKSGWDQYGMAVARPS

SNIVIRRISGTTRTCSGVGIGSEMSGGIFNITVEDIHVWDSAAGLRIKTDKGRGGYISNI

T---------FNNVLLEKVKVPIRFSSGSNDHSDDK------------------------

------------WDPKALPRVKGIYISNVVSLNSRKAP------MLLGVEGTSFQDVCLR

NVTLLGLPKT-------------EKWKCKDVSGYASD-VFPLSC----------------

>Osi004476.1

-------GGGGARLEVP---PGRWVTGSFNLTSRFTLFLHHGAIILGSQ-----------

-----------------------------DPEEWPLIAPLPSYGRGRERLGP--RHISLI

HGEGLDDVVITG--NNGTIDGQG--------RIWWDLWWNRTLN-HTRGHLI--------

-----------------------ELVDSTN-----------------------------I

MISNITLRNSPFWTVHPVYCRNVVIRN-LTVLAPLNAPN-TDGIDPDSSSEVCIEDCYIE

SGDDLVA-------------------------------------VKSGWDQYGISVGKPS

SNIIIQRVSGTTPTCSGVGFGSEMSGGISNVIIRDLHVWNSAQAVRIKTDVGRGGYITNI

T---------IENVRMEKVKVPIRFSRGADDHSDDK------------------------

------------YDRSALPKISDVRIRDVVGVDLQRAP------MLEAVHGAVYEGICFR

NVSLTVIKRQ-------------DRWHCESVYGEAHD-VLPAPC----------------

>At3g57790

---------SICRVVFPS---GNYLTAKLHLRSGVILDVTENAVLLGGPR----------

---------------------IEDYYPAETSSDWYVVVANNATDVGITGGGA-------I

DGQGSKFVVRFD------EKKNV--------MVSWNQTGACLGD-ECRPRLV--------

-----------------------GFVDSIN-----------------------------V

EIWNITLREPAYWCLHIVRCENTSVHD-VSILGDFNTPN-NDGIDIEDSNNTVITRCHID

TGDDAIC-------------------------------------PKT---YTGPLYNLTA

TDCWIRTKS------SAIKLGSASWFDFKGLVFDNITIFESHRGLGMQIR--DGGNVSDV

T---------FSNINISTRYYDPSWWGRAEPIYITT------------------------

------------CPRDSSAKEGSISNLLFVNITIDSENG----VFLSGSPNGLLSDIKFK

NMNLTFRRWSN-----------YSAGLVDYRPGCQGLVNHRAT-----------------

>Osi000936.3

------AAAGGGRVLLPA--PGDYLTATVHLRSRVVLDVAPGARLLGGTR----------

---------------------QADYPP--ESRRWYVVLAENTTGAGVTGGGE-------I

NGQGGAFVVTPN------PQKNI--------MVSWNATGDCEGD-ECRPRLV--------

-----------------------GFIDSKD-----------------------------V

TIHDITLNQPAYWC----------------------------------------------

------------------------------------------------------------

----------------------ASFFDFKKLVFDNITIVDSHRGLGMQIR--DGGNVSDV

V---------FSNIKMSTRYYHPLWWGRAEPIYITT------------------------

------------CPRHPDSKEGTISDIQFINISSVSENG----VFLAGSKHGLLRNLKFK

NVDLTYKRWTN-----------YSGGLYDYRPGCQ-------------------------

>Ec_peh1

--------GQGKAVKLSAGSSSVFLSGPLSLPSGVSLLIDKGVTLRAVN-----------

----------------------------------NAKSFENAPSSCGVVDTNGKGCDAFI

TATSTTNSGIYGP---GTIDGQGGVKLQDKKVSWWDLAADAKVKKLKQNTPR--------

---------------------LIQINKSKN-----------------------------F

TLYNVSLINSPNFHVVFSDGDGFTAWK-TTIKTPSTARN-TDGIDPMSSKNITIAHSNIS

TGDDNVAIKAYK--------------------------------GRSETRNISILHNEFG

TG-HGMSIG-------------SETMGVYNVTVDDLIMTGTTNGLRIKSDKSAAGVVNGV

R---------YSNVVMKNVAKPIVIDTVYEKK----------------------------

-------------EGSNVPDWSDITFKDITSQTKGVVV-------LNGENAKKPIEVTMK

NVKLTS----------------DSTWQIKNVTVKK-------------------------
